# From genes to Black Rust: genomic insights into corrosive methanogens

**DOI:** 10.1093/femsmc/xtaf018

**Published:** 2025-11-17

**Authors:** Sherin Kleinbub, Joseph J Braymer, Friedhelm Pfeiffer, Mike Dyall-Smith, Kristin Spirgath, Gabriela Alfaro-Espinoza, Andrea Koerdt

**Affiliations:** Division 4.1 Biodeterioration and Reference Organisms, Federal Institute for Materials Research and Testing (BAM), 12205, Berlin, Germany; Division 1.8 Environmental Analysis, Federal Institute for Materials Research and Testing (BAM), 12489, Berlin, Germany; Computational Systems Biochemistry, Max-Planck-Institute of Biochemistry, 82152, Martinsried, Germany; Computational Systems Biochemistry, Max-Planck-Institute of Biochemistry, 82152, Martinsried, Germany; Division 4.1 Biodeterioration and Reference Organisms, Federal Institute for Materials Research and Testing (BAM), 12205, Berlin, Germany; Division 4.1 Biodeterioration and Reference Organisms, Federal Institute for Materials Research and Testing (BAM), 12205, Berlin, Germany; Division 4.1 Biodeterioration and Reference Organisms, Federal Institute for Materials Research and Testing (BAM), 12205, Berlin, Germany

**Keywords:** methanogenic archaea, microbiologically influenced corrosion, MIC core, [NiFe]-hydrogenase, cell wall, mobile genetic elements

## Abstract

Within the past ten years, genetic evidence has been increasing for the direct role that microbes play in microbiologically influenced corrosion (MIC), also known as biocorrosion or biodeterioration. One prominent example is the correlation between the corrosion of metal and the presence of genes encoding an extracellular [NiFe]-hydrogenase (MIC hydrogenase) in the methanogenic archaeon, *Methanococcus maripaludis*. In this study, DNA sequencing and bioinformatic analysis were used to classify the MIC hydrogenase as belonging to a core set of genes, the MIC core, found so far in *Methanococci* and *Methanobacteria* classes of methanogens. Genetic evidence is provided for the mobilization of the MIC core via multiple mechanisms, including a horizontal gene transfer event from *Methanobacteria* to *Methanococci* and a newly described MIC-transposon. A detailed comparison of *M. maripaludis* genomes further pointed to the relevance that cell wall modifications involving N-glycosylation of S-layer proteins and the MIC hydrogenase likely play in methanogen-induced MIC (Mi-MIC). Microscopic analysis of corrosive methanogens encoding the MIC core indicated that *Methanobacterium*-affiliated strain IM1 can form extensive biofilms on the surface of corrosion products whereas individual cells of *M. maripaludis* Mic1c10 were only found localized to crevices in the corrosion layer. An updated model of Mi-MIC involving two modes of action is presented, which predicts that the propensity of cells to adhere to iron surfaces directly influences the rate of corrosion due to the localization of the MIC hydrogenase at the metal-microbe interface.

## Teaser

Genetic analysis indicated that a core set of genes correlated to biocorrosion has spread amongst certain archaea and the rate of corrosion is influenced by the composition of the cell wall.

## Introduction

Various microbes have the capacity to colonize and promote the deterioration of metal at metal-microbe interfaces, a process known as microbiologically influenced corrosion (MIC), also referred to as biocorrosion or biodeterioration (Qi et al. 2025). MIC plays a significant role in damages of steel and other metallic infrastructure across many economic sectors (Camara et al. 2022, Xu et al. 2023). Whereas MIC can occur in both aerobic and anaerobic environments, anaerobes facilitating the oxidation of elemental iron to the ferrous state (equation 1) have received the most attention. A variety of electron acceptors can be coupled to this redox reaction ranging from protons (equation 2), metals, inorganic ions, organic molecules, and proteins (Xu et al. 2023).


(1) \begin{eqnarray*}
F{e}^0 \rightleftharpoons F{e}^{2 + } + 2{e}^ -
\end{eqnarray*}

(2) \begin{eqnarray*}
F{e}^0 + 2{H}^ + \to F{e}^{2 + } + {H}_2
\end{eqnarray*}

The plethora of chemical species along with the complexity of biofilms makes studying the mechanisms of MIC a challenge in addition to the material and engineering aspects of metal that need to be considered (Knisz et al. 2023).

Sulfate-reducing bacteria (SRB) have long been thought to be a culprit of MIC as they can be found associated to metal surfaces, have been isolated on damaged metallic materials, and produce highly corrosive hydrogen sulfide (H_2_S) as a metabolic product (Enning and Garrelfs 2014). Corrosion by H_2_S is an indirect mechanism as cells are not required to be in direct contact with a metallic surface and corrosion may merely be a negative side effect of microbial growth (indirect MIC). It is now more widely recognized that in addition to SRB and other bacteria, many different microbes can participate in MIC including archaea, fungi, algae, and lichen, building on a hypothesis of corrosion influenced by microbes posited nearly a century ago (von Wolzogen Kühr and van der Vlugt 1934, Puentes-Cala et al. 2022, Khan et al. 2023). Some bacteria and archaea that depend on anaerobic H_2_ metabolism have been observed to consume electron equivalents from Fe° faster than the abiotic production of H_2_ from protons in an aqueous environment (reaction 2) (Daniels et al. 1987, Dinh et al. 2004, Mori et al. 2010, Uchiyama et al. 2010, Enning et al. 2012, Venzlaff et al. 2013, Beese-Vasbender et al. 2015, Deng et al. 2015, Deutzmann et al. 2015). These findings indicated a direct microbial contribution to corrosion, in which the microorganisms can harvest energy equivalents directly from metal (direct MIC), *e.g*. through direct electron uptake from iron surfaces or by producing molecular species, such as hydrogenases, that can catalyze the formation of H_2_ as an intermediary electron carrier.

Genetic and proteomic studies in the highly corrosive SRB *Desulfovibrio ferrophilus* IS5 and in *Geobacter sulfurreducens* have more recently provided evidence for the direct electron withdrawal hypothesis by identifying extracellular *c*-type cytochromes as proteinaceous species promoting direct electrical contacts at the metal-microbe interface (Tang et al. 2019, Chatterjee et al. 2021). Around the same time, the discovery of an unstable genetic island (MIC island) encoding an extracellular [NiFe]-hydrogenase (MIC hydrogenase) in the closely related methanogenic archaeal strains *Methanococcus maripaludis* OS7 and KA1 provided direct genetic evidence that microbes have evolved specific mechanisms to catalyze H_2_ production on elemental iron, in turn greatly accelerating corrosion (Tsurumaru et al. 2018). Only during growth on iron as the sole electron donor was the MIC island required. Providing H_2_ as the sole electron donor in laboratory conditions led to the loss of this genetic element in those strains. Detecting genes encoding the MIC hydrogenase or a *c*-type cytochrome, or immunodetection of the MIC hydrogenase itself, have successfully been used for identifying MIC in the field (Lahme et al. 2021, Lahme et al. 2022, Lahme and Aguas 2025, Lahme et al. 2025).

The MIC hydrogenase is a heterodimeric [NiFe]-hydrogenase that is hypothesized to be a causative agent of MIC acting to catalyze the reduction of protons (H^+^) to hydrogen (H_2_) via the abstraction of electrons from Fe^0^ and in turn solubilizing Fe^2+^ (reaction 2) (Shafaat et al. 2013, Tsurumaru et al. 2018). This enzymatic production of H_2_ for driving microbial metabolism via methanogenesis would explain the ability of corrosive *M. maripaludis* strains to thrive on metallic surfaces of oil-producing facilities from which they were isolated (Mori et al. 2010, Uchiyama et al. 2010, Tsurumaru et al. 2018, Lahme et al. 2021). A MIC island has also been found in the methanogenic strains *M. maripaludis* MIC098Bin5 and *Methanobacterium congolense* strain Buetzberg (Tsurumaru et al. 2018, Lahme et al. 2021). Along with the large and small subunits of the hydrogenase, the MIC island in *M. maripaludis* encodes a protease responsible for maturing the large subunit (Pinske et al. 2019). Also encoded in the MIC island is a twin arginine translocation (TAT) secretion system composed of TatA and TatC for the transport of the enzyme through the cell membrane. Additionally, the MIC island encodes other genes of so far unknown relevance to corrosion.

Our previous work has demonstrated the corrosivity of some methanogenic archaea, and that under dynamic conditions, corrosion rates on carbon steel caused by cultures of *Methanobacterium*-affiliated strain IM1 can surpass those of *D. ferrophilus* IS5 (An et al. 2020, An et al. 2021). Therefore, methanogen-induced MIC (Mi-MIC) is a highly relevant threat to metallic infrastructure. In those studies, the strains IM1 and *M. maripaludis* Mic1c10 (the genomes of which were not sequenced at the time), were more corrosive than the previously sequenced KA1 strain of *M. maripaludis*. To identify corrosive traits of Mic1c10 and IM1, we carried out a genomic comparison between corrosive and non-corrosive methanogens. The presence of the MIC hydrogenase in IM1 and Mic1c10 was confirmed and allowed for the identification of a MIC core responsible for promoting MIC. Bioinformatic analysis was performed to identify other MIC-related traits in *M. maripaludis* and to predict how *Methanococci* and *Methanobacteria* mobilize the MIC core. Electron microscopy was employed to confirm the presence of cells on corrosion products. The genomic elements and phenotypes of methanogens described here are used to develop a comparative model of biocorrosion involving the MIC hydrogenase.

## Materials and methods

### Medium for cell cultivation and stock cultures

Both *M. maripaludis* Mic1c10 and *Methanobacterium-*like IM1 cells were grown in anoxic artificial seawater medium (ASW; 26.37 g L^−1^ NaCl, 11.18 g L^−1^ MgCl_2_ x 6H_2_O, 1.47 g L^−1^ CaCl_2_ x 2H_2_O, 0.6 g L^−1^ KCl, 0.66 g L^−1^ KH_2_PO_4_ and 0.25 g L^−1^ NH_4_Cl) (Widdel and Bak 1992), supplemented with 1 mL L^−1^ selenite-tungstate solution (0.02 mM), 1 mL L^−1^ trace element solution (5.2 g L^−1^ Na-EDTA, 2.1 g L^−1^ FeSO_4_ x 7H_2_O, 0.03 g L^−1^ H_3_BO_3_, 0.1 g L^−1^ MnCl_2_ x 4H_2_O, 0.19 g L^−1^ CoCl_2_ x 6H_2_O, 0.024 g L^−1^ NiCl_2_ x 6H_2_O, 0.002 g L^−1^ CuCl_2_ x 2H_2_O, 0.144 g L^−1^ ZnSO_4_ x 7H_2_O and 0.036 g L^−1^ Na_2_MoO_4_ x 2H_2_O, pH adjusted to 6.0) (Widdel and Bak 1992), 1 mL L^−1^ vitamin solution (4 mg 4-aminobenzoic acid, 1 mg D(+)-biotin, 10 mg nicotinic acid, 5 mg calcium D(+)-pantothenate and 15 mg pyridoxine dihydrochloride in 100 mL 10 mM sodium phosphate buffer pH 7.0), riboflavin (0.664 μM), lipoic acid (0.727 μM) and folic acid (0.906 μM), as well as sodium acetate (1 mM). Sodium sulfide (1 mM) and cysteine (1 mM) were added as reducing agents (Dinh et al. 2004). The medium was buffered with NaHCO_3_ (60 mL of a 1 M stock solution were added per L) and with a N_2_/CO_2_ (80:20, v/v) gas atmosphere to maintain a pH of 7.2–7.3. The CO_2_ from the N_2_/CO_2_ gas mixture serves further as carbon source for methanogenesis. Medium prepared as such is from here on referred to as ASW medium. Stock cultures of *M. maripaludis* Mic1c10 and *Methanobacterium* IM1 were cultivated in 60 mL serum bottles filled with 20 mL ASW in the presence of 2 g of iron granules (1–2 mm, 99.98%; Alfa Aesar) under an N_2_/CO_2_ (80:20, v/v, 1 bar) gas atmosphere, under stationary conditions at 30°C (*Methanobacterium* IM1) or 37°C (*M. maripaludis* Mic1c10). Cultures of *M. maripaludis* Mic1c10 and *Methanobacterium* IM1 were transferred into freshly prepared ASW media every seven days or two weeks, respectively. Stock cultures of *M. maripaludis* S2 and JJ were cultivated similarly with DSMZ141 medium and ASW respectively, under an H_2_/CO_2_ (80:20, v/v, 1 bar) gas atmosphere under stationary conditions at 37°C and were transferred into fresh media every seven or 14 days, respectively.

### Cultivation of Mic1c10 for genome sequencing

*Growth on H_2_* (isolation of Mic1c10mut1): Cultivation of *M. maripaludis* Mic1c10 on H_2_ resulted in the loss of the MIC island, as revealed by subsequent genome sequencing (see below). The resulting mutant strain was named Mic1c10mut1, consistent with the spontaneous mutant obtained in strain OS7 (*M. maripaludis* OS7mut1) described by Tsurumaru *et al*. (Tsurumaru et al. 2018). For growth on H_2_, 10 mL of *M. maripaludis* Mic1c10 preculture, grown under the same conditions as the stock cultures, were added to several 2 L anaerobic bottles, each with 1 L fresh ASW medium under H_2_/CO_2_ gas atmosphere (80:20%, v/v, 1 bar). Cultures were incubated horizontally at 30°C for ten days under static conditions. To provide sufficient CO_2_ for methanogenesis, the headspace of each bottle was flushed with H_2_/CO_2_ (80:20%, v/v, 1 bar) for five minutes after five days of growth. After incubation, cells were pelleted by centrifugation for 10 min at 14 000 x g at 4 °C. The pellet was washed once with phosphate-buffered saline (PBS, 4 mM KH_2_PO_4_, 16 mM Na_2_HPO_4_, 115 mM NaCl), centrifuged again, and sent to the commercial sequencing provider Eurofins Genomics (Eurofins Genomics GmbH, Ebersberg, Germany) for DNA extraction, quantification, quality control and genome sequencing. For PacBio (PacBIO RS II) sequencing, the DNA library was prepared by Eurofins Genomics. The kits from PacBio were used according to the manufacturer’s instructions (DNA template preparation kit v3.0; DNA/polymerase binding kit P6; DNA sequencing kit; MagBead kit; SMRT cell 8pac). Sequencing resulted in a total of 1 297 668 283 bases for 171 228 reads with a mean read length of 6,492 nt (N50 9,428 nt). To resolve potential frameshift errors, which may be encountered upon PacBio sequencing, the same material that had been sent for PacBio sequencing was subjected to Illumina sequencing by Eurofins Genomics with a HiSeq instrument (sequencing mode: NovaSeq 6000 S1 PE150 XP), generating 22 526 200 high quality paired reads (3.38 Gbases).

*Growth on Fe* (cultivation of Mic1c10)*: M. maripaludis* Mic1c10 was precultured at 30 °C for one week in ASW medium in the presence of iron granules (1.5% (w/v); 1-2 mm, 99.98% purity; Alfa Aesar) under a N_2_/CO_2_ (80:20%, v/v, 1 bar) gas atmosphere. After incubation, 12 mL of the preculture were added to several 1 L anaerobic bottles each with 600 mL ASW medium under N_2_/CO_2_ (80:20%, v/v, 1 bar), supplemented with 24 g of iron granules. Cultures were incubated horizontally at 30°C for ten weeks under static conditions. To provide sufficient CO_2_ for methanogenesis, the headspace of each bottle was flushed weekly with N_2_/CO_2_ (80:20%, v/v, 1 bar) for five minutes. After incubation, cells were pelleted and washed with PBS as described above, then sent to Eurofins Genomics for Illumina sequencing with a HiSeq instrument (sequencing mode NovaSeq 6000 S1 PE150 XP), which yielded 9 163 192 high quality paired reads (1.38 Gbases).

During both growth experiments, methane (CH_4_) formation in the culture headspace was determined regularly with a gas chromatograph (8890 GC System; Agilent) to monitor the growth of the cultures.

### Genome assembly of Mic1c10mut1 and Mic1c10

An automated genome assembly of the PacBio sequences (originating from Mic1c10 grown on H_2_ as electron donor) was performed by Eurofins, using the SMRT^®^ Analysis software (Basemode tool) (Chin et al. 2013), which runs HGAP (DAGCON-based hierarchical genome assembly process, RS_HGAP_assembly.3 version 2.3.0) with the following three steps (Chin et al. 2013): pre-assembly, *de novo* assembly with the Celera assembler and final polishing with Quiver. The assembly resulted in a single 1.7 Mb contig with 559-fold coverage, which showed terminal redundancy but additionally contained a long terminal duplication of 31 kb. Reads at the junction of the duplication were evaluated in detail, which confirmed the biological origin of the duplication. In the initial assembly, the two copies of the duplication deviated in sequence. This was resolved by co-assembly of PacBio and Illumina sequences using a combination of Canu (Koren et al. 2017) and the Geneious (ver.10.2.6) mapper tool (Kearse et al. 2012), as has been used when assembling the genome of *Halobacterium salinarum* strain 91-R6 (Pfeiffer et al. 2019, Pfeiffer et al. 2020). Only a single point mutation persisted between the two copies of the 31 kb duplication, proving the validity of the dual sequencing technique approach.

The point of ring opening was adjusted to the chromosome of a closely related *M. maripaludis* strain (KA1; GenBank: AP011526), which is also consistent with other genomes from *M. maripaludis* (see also Suppl. Text S1). Unlike KA1, sequencing reads for a MIC island associated with corrosive behavior were not identified. This led to the hypothesis that the MIC island had been completely lost upon growth under H_2_, and the culture was assigned as Mic1c10mut1 (see Suppl. Text S2). A second round of sequencing of the sample was pursued using cultures grown under selection conditions. The sequencing of Mic1c10 grown only on iron granules revealed the MIC island, bounded by 221 bp direct repeats, consistent with results obtained for strain OS7 (Tsurumaru et al. 2018). After validation that several Illumina reads traverse the 221 bp direct repeat at both ends of the MIC island, its sequence was integrated manually into the Mic1c10mut1 chromosome sequence to result in the complete sequence of the Mic1c10 chromosome.

### Annotation of the *M. maripaludis* Mic1c10 genome

A detailed annotation of the *M. maripaludis* Mic1c10 genome was performed (see Suppl. Text S3). Initial gene prediction was performed using RAST (Overbeek et al. 2014). Open reading frame (ORF) calling, especially start codon assignment, was checked against the proteomes from eight completely sequenced strains from the genus *Methanococcus* (see Suppl. Table S1). Intergenic regions longer than 50 bp were analyzed for coding capacity (BLASTx to the NCBI nr database) to detect and resolve missing gene calls. Stable RNAs (rRNAs, tRNAs, 7S RNA, RNAseP RNA) were annotated according to RFAM (Kalvari et al. 2018, Kalvari et al. 2021) and stable RNAs annotated for *M. maripaludis* strain S2 (GenBank: BX950229) (see Suppl. Text S4). DNA methylation and tetramer analysis along with CRISPR-Cas identification were carried out as described in Supplement Texts S5-6. The annotation of protein-coding genes was enhanced using a simplified version of the Gold Standard Protein-based manual curation strategy (Pfeiffer and Oesterhelt 2015). The well-annotated genome of *M. maripaludis* S2 was used as reference (Hendrickson et al. 2004, Goyal et al. 2014, Goyal et al. 2016). Further information sources were the SwissProt section of UniProt (Uniprot 2023) and the proteomes from 12 haloarchaeal genomes under continuous annotation survey (Pfeiffer and Oesterhelt 2015, Pfeiffer et al. 2020).

### Comparison of the *M. maripaludis* Mic1c10 genome to those of *M. maripaludis* strains KA1 and OS7

The chromosome from *M. maripaludis* Mic1c10 (initially using that of Mic1c10mut1) was compared separately to those of *M. maripaludis* strains KA1 and OS7, using the same strategy that was previously applied to strains of *Halobacterium salinarum* (Pfeiffer et al. 2020). Briefly, sequence chunks of 400 kb were aligned using MAFFT (Katoh and Standley 2013) and matching segments (matchSEGs, typically 97–99% nucleotide sequence identity) or divergent sequences (divSEG) were identified (see Suppl. Text S2). The start of the last matchSEG was used as the starting point for the next chunk of 400 kb. By this approach, the complete chromosome was traversed (see Suppl. Text S2). Taxonomical analysis (Suppl. Table S2) was carried out as described in Supplemental Text S7. The complete genome of *M. maripaludis* Mic1c10 was also compared in detail to the draft genome which became publicly available at a late stage of the current study (See Suppl. Text S8) (Kawaichi et al. 2024).

### Genomic DNA extraction, amplification by Polymerase Chain Reaction (PCR) and sequencing of a MIC island from *Methanobacterium* IM1

Genomic DNA (gDNA) of *Methanobacterium* IM1 was extracted by the phenol-chloroform method. Cell cultures were grown under static conditions at 30°C, for two weeks in 25 mL anaerobic ASW medium (headspace N_2_/CO_2_ (80:20, v/v, 1 bar)), supplemented with 2 g of iron granules. After incubation, 5 mL of the culture were harvested and centrifuged at 8 000 x *g* for 2 min. The pellet was washed twice with 400 µL sodium-Tris-EDTA (STE) buffer (100 mM NaCl, 10 mM Tris/HCl, 1 mM EDTA, pH 8.0). Subsequently, the pellet was resuspended in 200 µL Tris-EDTA (TE) buffer (10 mM Tris/HCl, 1 mM EDTA, pH 8.0). Then 100 µL Tris saturated phenol (pH 8.0, Lab-Scan Analytical Sciences) was added and cells were lysed by vigorous vortexing for 60 sec. The sample was centrifuged for 5 min at 13 000 x *g* and 4°C to separate the aqueous from the organic phase. Then, 160 µL of the aqueous phase was mixed with 40 µL TE buffer and 100 µL chloroform (Sigma) and subsequently centrifuged (5 min, 13 000 x *g*, 4°C). The previous step was repeated. Then the sample was centrifuged again to separate the two phases. Purified gDNA was obtained in the upper phase after the last centrifugation step. The DNA was either used directly or stored in aliquots at -20°C until further use. The purity and yield of the DNA was assessed spectrophotometrically with NanoDrop 2000 (Thermo Scientific).

To sequence the MIC island of *Methanobacterium* IM1, oligonucleotides were designed based on a genome sequence (unpublished data) kindly provided by Dr. F. Musat (Aarhus University, Denmark) and Prof. F. Widdel (MPI for Marine Microbiology; Bremen, Germany). *In vitro* amplification of DNA fragments was done in a C1000 Touch^TM^ Thermal Cycler (Bio-Rad Laboratories, Inc.; Hercules, California, United States). For amplification, Q5^®^ High-Fidelity DNA Polymerase (NEB: New England Biolabs; Frankfurt a. M., Germany) was used according to the manufacturer’s instructions. Oligonucleotides used for PCR and sequencing are listed in Supplementary Table S3. All DNA fragments were purified using Monarch PCR & DNA cleanup kit from NEB and sent for Sanger sequencing to LGC Genomics (LGC Genomics GmbH, Berlin, Germany). DNA contigs were assembled using SnapGene software.

### Cultivation of methanogens on surfaces and microscopic analysis

Static cultures of methanogens were incubated at 30°C in ASW medium in the presence of pre-treated iron coupons (99.5% Fe^0^, 0.3% Mn, 0.08% C, 0.04% P; 10 mm x 10 mm x 1 mm; Goodfellow GmbH, Germany) according to the NACE protocol SP0775-2013 (NACE 2013). After 24 days under a N_2_/CO_2_ gas atmosphere (80:20%, v/v, 1 bar), the iron coupons were transferred to a PBS/dH_2_O (1:1) solution for 1 min. Then, the biofilm was fixed with 2.5% (w/v) glutaraldehyde at 4°C overnight. After fixation, the coupons were washed with PBS/dH_2_O (1:1) for 5 min, followed by a washing step with distilled water for another 5 min. Dehydration was performed by the following incubations: 30 min in 30% ethanol, 30 min in 50% ethanol, 30 min in 70% ethanol, 60 min in 80% ethanol, 60 min in 90% ethanol, 2×60 min in absolute ethanol. After drying under a flow of N_2_, the coupons were sputtered with a 4-5 nm thick gold layer and imaged via scanning electron microscopy (7.00 kV, 4.5 mm; SEM Evo MA10; Zeiss, Wetzlar, Germany or 5.00 kV, 5.0 mm, SEM Gemini Supra 40, Zeiss, Wetzlar, Germany).

Additionally, *M. maripaludis* Mic1c10 and *Methanobacterium* IM1 were grown on glass coupons for five days under static conditions in ASW medium under H_2_/CO_2_ gas atmosphere (80:20%, v/v, 1 bar). The glass coupons were carefully washed with 0.9% NaCl solution followed by incubation with SyproOrange^®^ Gel Stain (1:5000 dilution in 0.9% NaCl; Sigma-Adrich, St. Louis, United States) for 30 min, followed by a second wash in 0.9% NaCl to remove excess staining solution. Biofilms were analyzed with confocal laser scanning microscopy (CLSM) (Leica SP8; Leica Microsystems CMS GmbH, Mannheim, Germany).

### Additional bioinformatic tools

As general tools, MUMMER (Delcher et al. 2003) and the BLAST suite of programs (Altschul et al. 1997, Johnson et al. 2008) were used for genome comparisons. Gene clusters were searched for and aligned using cblaster and clinker, respectively (Gilchrist and Chooi 2021). Protein and DNA sequences were aligned with either CLUSTALW (https://www.genome.jp/tools-bin/clustalw) or Multalin (http://multalin.toulouse.inra.fr/multalin/). The CRISPR finder web server (http://crispr.i2bc.paris-saclay.fr) and the CRISPRCasFinder server (https://crisprcas.i2bc.paris-saclay.fr/) were used to search for CRISPR elements and to classify the CRISPR system (Grissa et al. 2008). The viral/spacer BLAST server (https://img.jgi.doe.gov/cgi-bin/vr/main.cgi) was used to find spacer matches to the *M. maripaludis* genome. The online tool PHASTEST (https://phastest.ca/) was used to search for prophages (Arndt et al. 2016, Arndt et al. 2019). ISfinder was used to search for transposons (https://isfinder.biotoul.fr/) (Siguier et al. 2006). *In silico* DNA-DNA hybridization (DDH) values were calculated using the Genome-to-Genome Distance Calculator (GGDC) 2.1 server (http://ggdc.dsmz.de/ggdc.php). ANIb (average nucleotide identity, BLASTn) values were determined using the JSpecies server (http://jspecies.ribohost.com/jspeciesws). Circular genome maps were created using the CGView (https://github.com/paulstothard/cgview). Phylogenetic tree reconstruction was carried out with either FastME 2.1.6.1 (Lefort et al. 2015) or MEGA11 (Tamura et al. 2021). Genomic island (GI) prediction used Island Viewer 4 (http://www.pathogenomics.sfu.ca/islandviewer/) described by (Bertelli et al. 2017). The prediction of Asn-Xaa-Ser/Thr sequons as glycosylation sites was carried out with NetNGlyc–1.0 (https://services.healthtech.dtu.dk/services/NetNGlyc-1.0/). Models of unknown protein structures were either accessed from the AlphaFold Protein Structure Database (https://alphafold.ebi.ac.uk/) (Jumper et al. 2021, Varadi et al. 2024) or generated with the AlphaFold Server (https://alphafoldserver.com/) (Abramson et al. 2024).

## Results

### MIC island identification and mobilization

**Figure 1.**
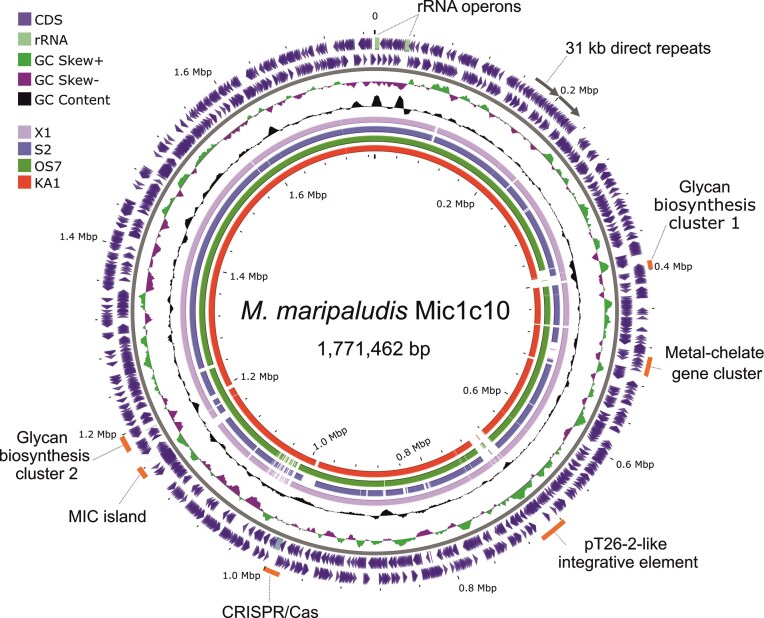
Genome map of *M. maripaludis* Mic1c10 compared to four other strains of *M. maripaludis* (X1, S2, OS7, and KA1). The color key at top left identifies various components and rings of the map, from outside to the center. Outermost and innermost are size scales, in Mbp. Rings 1 and 2 represent the *M. maripaludis* Mic1c10 gene map with CDS (blue) in the reverse (outer) and forward (inner) direction. Regions of interest are indicated (rRNA genes, light green boxes; 31 kb direct repeats, grey arrows; gene clusters, orange lines). Ring 3 indicates GC-skew, with green colored peaks representing higher values and purple peaks representing lower values. Ring 4 shows deviations from average %GC (33%), with higher values directed outwards and lower values directed towards the center. Rings 5 to 8 are BLASTn comparisons of Mic1c10 to the indicated strains, where colored lines represent significant nucleotide sequence similarity (E-value ≤ 10^−10^). The map and plots were made using the CGView tool.

**Figure 2.**
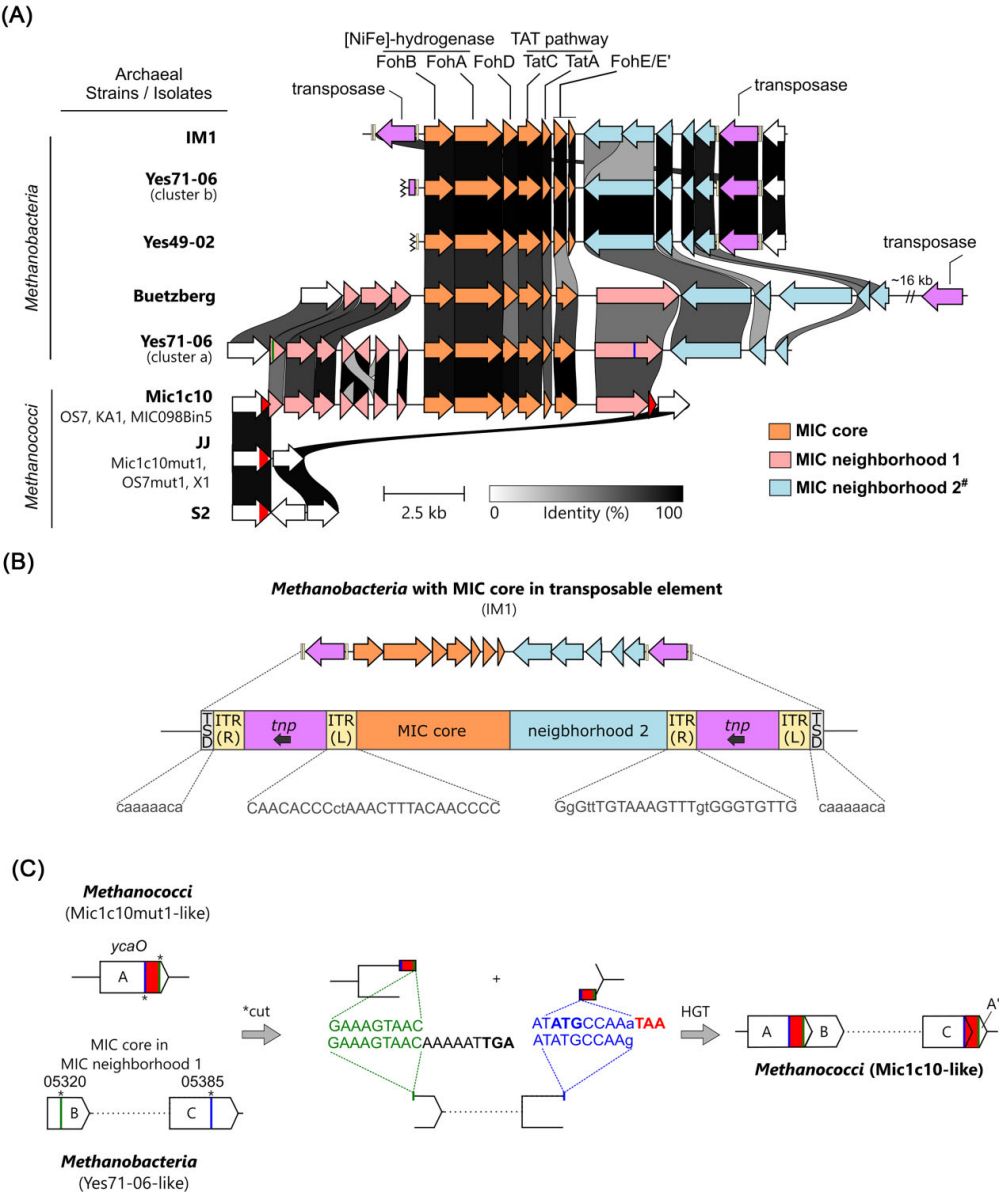
Gene cluster representation of MIC genetic elements in two classes of methanogens and three modes of mobilization. (**A**) The MIC core (orange, genes labelled by the respective encoded proteins) refers to a conserved set of 6–7 genes, including *fohAB* and associated downstream genes that are hypothesized to be essential for corrosion. Depending on strain or isolate, the MIC core is flanked by two different MIC gene neighborhoods (pink and blue). In *M. maripaludis* Mic1c10, the MIC core nestled in MIC neighborhood 1 (MIC island) can be mobilized due to two copies of a near-identical 221 bp direct repeat (red) (Tsurumaru et al. 2018). The MIC cores in the *M. maripaludis* strains listed are highly similar but not identical to Mic1c10. ^#^The extent of MIC neighborhood 2 is putative and defined here by the genetic region between the MIC core and a transposon (purple). (**B**) A second mobilization of the MIC core in some *Methanobacteria* (colors as in A), is predicted to occur as a transposable element, which is fully defined in IM1. The transposon pair is enclosed by a target sequence duplication (TSD, grey), which is an 8 bp long direct repeat. Both transposons have prominent inverted terminal repeats (ITRs, yellow) (matching bases uppercased, mismatches in lowercase). The ITRs of the two transposons are closely related. Both transposases are encoded in the reverse strand. The ITR and TSD sequences are shown for the forward strand. (**C**) A third mobilization can be described as a horizontal gene transfer event (HGT) involving the acquisition of the MIC core in MIC neighborhood 1 in a Mic1c10mut1-like organism from a Yes71-06-like organism. Two 9 bp motifs (green and blue) shared between the two organisms explain the genetic integration and generation of the MIC island in *Methanococci* flanked by the two 221 direct repeats stemming from *ycaO* (red boxes, see also Suppl. Figs S4-5). Two newly introduced stop codons for genes A and C are highlighted in bold as well as a newly introduced start codon for gene A’. Only locus tag numbers for genes B and C from Yes71-06 are shown (locus tag prefix: QMD61).

The genome of *M. maripaludis* Mic1c10 was successfully sequenced from cells grown on iron granules as the sole electron donor (Fig. 1 and Suppl. Fig. S1). Taxonomical assignment of the resulting genome placed Mic1c10 within a clade also containing KA1, OS7, X1, and S2 (Suppl. Fig. S2 and Suppl. Table S2). Like the other known corrosive strains KA1 and OS7, Mic1c10 includes the 12 kb MIC island (Fig. 1) (Tsurumaru et al. 2018). This island is bounded by near-identical 221 bp direct repeats resulting in genetic instability. Within the MIC island of Mic1c10 is encoded the heterodimeric Fe oxidizing [NiFe]-hydrogenase, FohAB (Fig. 2A and Suppl. Fig. S3) (Tsurumaru et al. 2018, Lahme et al. 2021, Kawaichi et al. 2024). The large subunit FohA (MMic1c10_05 900) is identical to OS7 and KA1 whereas the small subunit (SSU) FohB (MMic1c10_05 895) shows 91% sequence identity to that of the two other strains. Notably, the read coverage across the MIC island was only one-third that of the rest of the genome (Suppl. Fig. S3). This suggested that the majority of cells in the population lacked this element, even though the cells were grown under selection conditions (Fe^0^). Growth on hydrogen resulted in a genome devoid of the MIC island and the corresponding strain is assigned as Mic1c10mut1 (Fig. 2A and Suppl. Fig. S3), consistent with a spontaneous mutant obtained in strain OS7 (*M. maripaludis* OS7mut1) (Tsurumaru et al. 2018). Similar instability is expected in KA1 and MIC098Bin5. The complete genome of Mic1c10 reported here is nearly identical to a recently published draft genome (Suppl. Text S8) (Kawaichi et al. 2024). Contig1 of that genome corresponds to Mic1c10mut1 while, due to extremely low coverage across the MIC island (Suppl. Fig. S3), the island is provided as its own contig (Contig3). The MIC island in the draft genome is not complete and does not fully cover its 3’-end, thus lacking part of the gene encoding a PAS domain-containing protein and the second copy of the direct repeat (Suppl. Fig S3B and Suppl. Text S8). Additionally, the full genome of Mic1c10 contains a duplication of a 31 kb region as a direct repeat, which is of unknown biological impact (Fig. 1 and Suppl. Fig. S3) but is absent from the draft genome.

IM1 is to-date one of the most corrosive characterized methanogens (Dinh et al. 2004, An et al. 2020, Tamisier et al. 2022). Its genome remains unpublished but has been kindly provided to us by Dr. F. Musat (Aarhus University, Denmark) and Prof. F. Widdel (MPI for Marine Microbiology, Bremen, Germany). By searching the genome, it was found that IM1 also harbors a gene encoding FohA with 93% identity to FohA from *M. maripaludis* Mic1c10. To identify a possible MIC island, regions surrounding the gene *fohA* were amplified via PCR and sequenced via Sanger sequencing (Suppl. Table S3). Assembly of the sequenced DNA led to the identification of seven genes with high similarity to six genes in the MIC island of corrosive *M. maripaludis* strains, including Mic1c10 (Fig. 2A). The sixth gene of Mic1c10 showing similarity is split in two in IM1 (*fohE/E*’ in Fig. 2, see below). These results confirm that the MIC hydrogenase is genetically encoded in IM1 and indicate that FohAB is a viable molecular candidate for explaining the ability of IM1 to carry out electromethanogenesis on graphite (Beese-Vasbender et al. 2015). The enzyme has previously been observed in cultures of IM1 (Lahme and Aguas 2025).

The seven genes of the gene cluster containing *fohAB* in IM1 are flanked by genes not seen in the genetic neighborhood of the MIC island in *M. maripaludis* strains. Yet, five genes downstream are comparable to four genes downstream of the MIC island previously identified in *Methanobacterium congolense* strain Buetzberg, where again one gene is split in two in IM1 (Fig. 2A) (Tejerizo et al. 2017, Tsurumaru et al. 2018). A BLASTp analysis with the protein sequence of IM1 FohA against the non-redundant protein sequence database detected several homologs in contigs from metagenomic assembled genomes (MAGs). These proteins are encoded in the isolates Yes49-02 and Yes71-06, originating from groundwater in the Yessentuki mineral deposit in Russia (GenBank accessions JASEFG010000015, JASEIM010000008, and JASEIM010000036). Yes49-02 belongs to the thermophilic *Methanothermobacter* genus while Yes71-06 is assigned to the *Methanobacterium* genus. Strikingly, Yes71-06 has two annotated *fohA* genes. One is found in a Mic1c10-like gene cluster (cluster a, Yes71-06a) and a second in an IM1-like gene cluster (cluster b, Yes71-06b, Fig. 2A). Gene cluster analysis indicated that both Yes-isolates retain four or five genes downstream of *fohAB* which are also found in the corrosive methanogens. These six or seven conserved genes likely represent a core set of genes required for FohAB to function, hereafter referred to as the MIC core. The MIC core (Fig. 2A, orange-colored genes) is distinguished from the broader MIC island previously described by Tsurumaru and colleagues (Tsurumaru et al. 2018). Adopting the nomenclature similar to other hydrogenases, the NiFe-hydrogenase maturation protease, MMic1c10_05 905 is labeled as FohD (Pinske et al. 2019). In addition to TatAC, all MIC cores encode also a γ-carbonic anhydrase-like protein (MMic1c10_05 920), which is assigned as FohE (see below). The MIC core therefore occurs in at least two types of genetic neighborhoods, detected in *Methanococci* (neighborhood 1, Mic1c10-like) and in *Methanobacteria* (neighborhood 2, IM1-like, Fig. 2A).

In IM1, the MIC core together with MIC neighborhood 2 is flanked by transposons on both sides (Fig. 2B). These transposons are distantly related to transposon ISM1 in ISFinder (Siguier et al. 2006), with 44% to 46% protein sequence identity of the encoded transposases but only restricted similarity at the DNA level. BLASTn analysis against the NCBI nr database identified a closely related transposon from *Methanobacterium alkalithermotolerans* (96% and 85% DNA sequence identity; submitted to ISFinder May-2025, processing pending), which has a prominent inverted terminal repeat (ITR). All four transposon copies in *M. alkalithermotolerans* are enclosed by an eight-nucleotide target sequence duplication (TSD). The transposons in IM1 are not individually enclosed by a TSD, but the eight nucleotides upstream of the preceding transposon are identical to the eight nucleotides downstream of the subsequent transposon (Fig. 2B). This is indicative that the transposon copies have not been mobilized separately, but that the complete region, including the two transposons, the MIC core, and the MIC neighborhood 2 have been acquired as one single composite element (MIC transposon). Moreover, the same pair of transposons flanks MIC neighborhood 2 in the draft genomes of strains Yes71-06 (cluster b) and Yes49-02, but in both cases the sequence of one of the transposons marks the contig end and is only partial (Fig. 2A-B). Strain Buetzberg also has a transposase from the IS5 family that is 16 kb downstream of MIC neighborhood 2 and it remains unclear if this transposase acts on the MIC-related genes. Overall, genetic evidence indicates that the MIC core has spread in mesophilic and thermophilic methanogens within the *Methanobacteriaceae* family via a transposon-associated mobilization event (Fig. 2).

Yes71-06a shows high identity to all fourteen genes of the MIC island in Mic1c10 in addition to several genes of neighborhood 2 (Fig. 2A). Analysis of Yes71-06a led to the identification of two motifs nine nucleotides long in the first (QMD61_05 320) and last gene (QMD61_05 385) that correspond to the 5’- and 3’ ends of the direct repeats in Mic1c10 (Fig. 2C and Suppl. Figs S4-5). With high identity across the MIC-related genes and the two motifs, Yes71-06a is a close ancestor to the MIC island in Mic1c10 (Fig. 2C). With only nine nucleotides, the two motifs are too short for homologous recombination (HR), but fall within the 5–25 nucleotide length range known from microhomology-mediated end joining (MMEJ), one of the repair mechanisms for DNA double strand breaks (McVey and Lee 2008, Xiong et al. 2025). Remarkably, cutting at or near these motifs in a Yes71-06-like organism predicts horizontal gene transfer of the MIC core in neighborhood 1 into the *M. maripaludis ycaO* gene as previously predicted (Tsurumaru et al. 2018). Furthermore, the genomic integration of the MIC island into a Mic1c10-like organism would introduce two new stop codons (Fig. 2C). One affects the *ycaO* gene, but with minimal effects: the C-terminal tetrapeptide would be replaced by an unrelated dipeptide (Suppl. Fig. S4). The other results in truncation of the last gene of the MIC island in Mic1c10 that would lead to the removal of a C-terminal domain from the PAS-domain containing protein (MMic1c10_05 925, Suppl. Fig. S5). In turn, the first gene of the MIC island (MMic1c10_05 860) might be truncated at the 5’-end as compared to the analogous gene, QMD61_05 320, in Yes71-06a (Fig. 2C). However, this could be resolved by an in-frame GTG which might function as a start codon, adding a novel N-terminal sequence (Suppl. Fig. S4). This novel N-terminus results in a considerable overlap with *ycaO*. An in-frame ATG within the second motif would function as a start codon and would generate a short protein that corresponds to the C-terminal region of *ycaO* (Fig. 2C). Hence, the two motifs define the two direct repeats in corrosive strains and predict that insertion could have occurred in any *Methanococcus* strain containing the conserved *ycaO* gene. Along with Yes71-06a, *Methanobacterium congolense* strain Buetzberg also has a fusion of the two MIC neighborhoods (Fig. 2A) (Tejerizo et al. 2017). Buetzberg is more distant to Mic1c10, however, because the motifs are more divergent and the last gene of MIC neighborhood 1, a PAS-domain containing protein, has an additional PAS domain in Buetzberg as compared to Yes71-06a and Mic1c10 (Suppl. Fig. S5).

### A mosaic of integrative gene clusters

Besides the MIC island in Mic1c10, two glycan biosynthesis clusters, a metal-chelate gene cluster, a pT26-2-like integrative element and CRISPR/Cas are highlighted in Fig. 1. Each of these gene clusters shows high variability to otherwise closely related strains (white gaps in the genome comparison rings). For each of these clusters, we performed an extensive inter-strain comparison. As only glycosylation could be envisioned to potentially impact corrosion behavior directly, only the glycan biosynthesis clusters are discussed in the main text while detailed information for the other gene cluster can be found in the Suppl. Material. For metal-chelate clusters see Suppl. Fig. S6 and Suppl. Text S9. For pT26-2 plasmids and related elements see Suppl. Fig. S6, Suppl. Table S4, and Suppl. Text S9. For CRISPR/Cas see Suppl. Fig. S7, Suppl. Table S5 and Suppl. Text S6.

### Glycan biosynthesis

**Figure 3.**
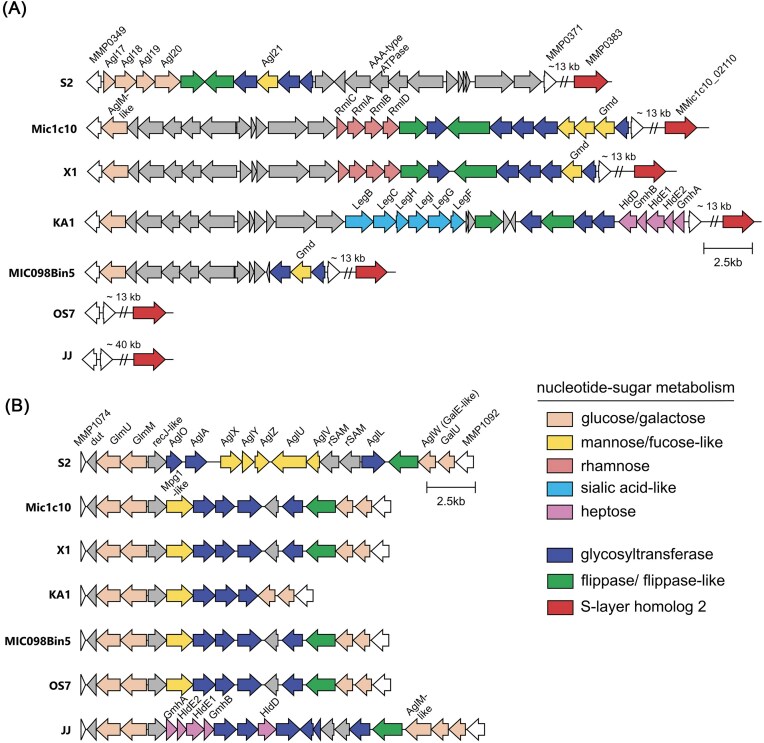
Glycan biosynthesis gene clusters in various *M. maripaludis* strains. (**A**) Glycan biosynthesis gene cluster 1 resides in a genetic region flanked by conserved genes (MMP0349/MMic1c10_1920 and MMP0371/MMic1c10_2050 in strains S2/Mic1c10). This cluster occurs at a short genomic distance to the gene for S-layer protein homolog 2 (red). (**B**) Glycan biosynthesis gene cluster 2 resides in a genetic region flanked by conserved genes (MMP1074/MMic1c10_6020 and MMP1092/MMic1c10_6090 in strains S2/Mic1c10). Each strain has its own set of sugar- and nucleotide-sugar modifying enzymes, glycosyltransferases, and putative flippases in the two biosynthesis gene clusters (see color coding in legend). Genes encoding proteins of interest are labelled. The size of longer intervening sequences is indicated by double-slashes, the size given as kb. The sorting of the strains is the same for panels A and B.

The divSEQ near 0.4 Mbp (MMic1c10_01 925 to MMic1c10_02 045) was identified as a gene cluster encoding nucleotide-sugar metabolism enzymes and probable glycosyltransferases as well as flippases (Fig. 1, glycan biosynthesis cluster 1). Such gene clusters can also be identified in the other *M. maripaludis* strains, yet with considerable diversity in the composition of genes (Fig. 3A). A gene cluster in X1 is very similar to that in Mic1c10. In the S2 strain, a highly distinct glycan biosynthesis cluster occurs in this genetic region, spanning from conserved MMP0349 to MMP0371, and includes five genes encoding enzymes for the modification of the second glucose-based and third mannose-based sugars of the archaeal tetrasaccharide (Agl17-21) (Siu et al. 2015) (Fig. 3A). Close homologs of Agl17-21 are not found in this region in Mic1c10. Instead, gene cluster analysis indicated glycan biosynthesis involves a gene encoding a putative UDP-glucose 6-dehydrogenase (AglM-like) (Yurist-Doutsch et al. 2010), four genes of the rhamnose pathway (RmlA-D) (Giraud and Naismith 2000) and three genes resembling a fucose-like pathway including a gene encoding GDP-mannose 4,6-dehydratase (Gmd) (Ginsburg 1961). Both the rhamnose and fucose-like pathways are encoded in operon-like clusters including putative flippase-like and glycosyltransferase genes (Fig. 3A). Interestingly, KA1 also has two operon-like clusters with completely different annotated genes. One shows homology to genes involved in sialic acid-like sugar biosynthesis (Zaretsky et al. 2018) and the second for ADP-heptose sugar biosynthesis (Kneidinger et al. 2002) (Fig. 3A). This region also contains several other uncharacterized genes, some of which are annotated as membrane proteins (grey genes, Fig. 3A). In contrast, this region in strain MIC098Bin5 has fewer genes and OS7 lacks this glycan biosynthesis cluster all together. The same genetic region in strain JJ is distinctly different and has a pT26-2-like mobile genetic element (Fig. 3A and Suppl. Fig. S6A).

The identification of glycan biosynthesis genes prompted us to also analyze a second known glycan biosynthesis cluster (Fig. 1, glycan biosynthesis cluster 2). Again, there is considerable diversity between strains. In *M. maripaludis* S2, the cluster extends from MMP1074 to MMP1092 and is responsible for the second, third, and fourth sugars of the glycan (AglXYZUV, Fig. 3B) (Jarrell et al. 2014, Ding et al. 2016). Analysis of the Mic1c10 genome identified a glycan biosynthesis region that included a divergent section, with no homologs to proteins responsible for glycan formation in strain S2 (Fig. 3B). Also, other corrosive and non-corrosive strains lack close homologs to these proteins. A putative mannose-1-phosphate guanylyltransferase (Mpg1-like) is encoded in Mic1c10, which would provide GDP-mannose for the Gmd enzyme found in glycan biosynthesis cluster 1. The second glycan biosynthesis cluster is also different in strain JJ, where an ADP-heptose pathway is found, which is analogous to a gene cluster in glycan biosynthesis cluster 1 in strain KA1. Initial assembly of the glucose and galactose components of the glycan though may be similar as enzymes responsible for these sugars are present (e.g. GlmU/M and AglW/GalU, Fig. 3B). All strains have AglB (Mic1c10_07 730) required for attaching glycans to cell wall proteins, *e.g*. S-layer proteins, pilin, and archaellin (Jarrell et al. 2014, Ding et al. 2016). Glycan biosynthesis cluster 2 is shared between strains Mic1c10, KA1, and OS7. Overall, the difference in both glycan biosynthesis gene clusters indicates that the glycan composition differs across corrosive *M. maripaludis* strains as well as non-corrosive ones.

### S-layer protein glycosylation

**Figure 4.**
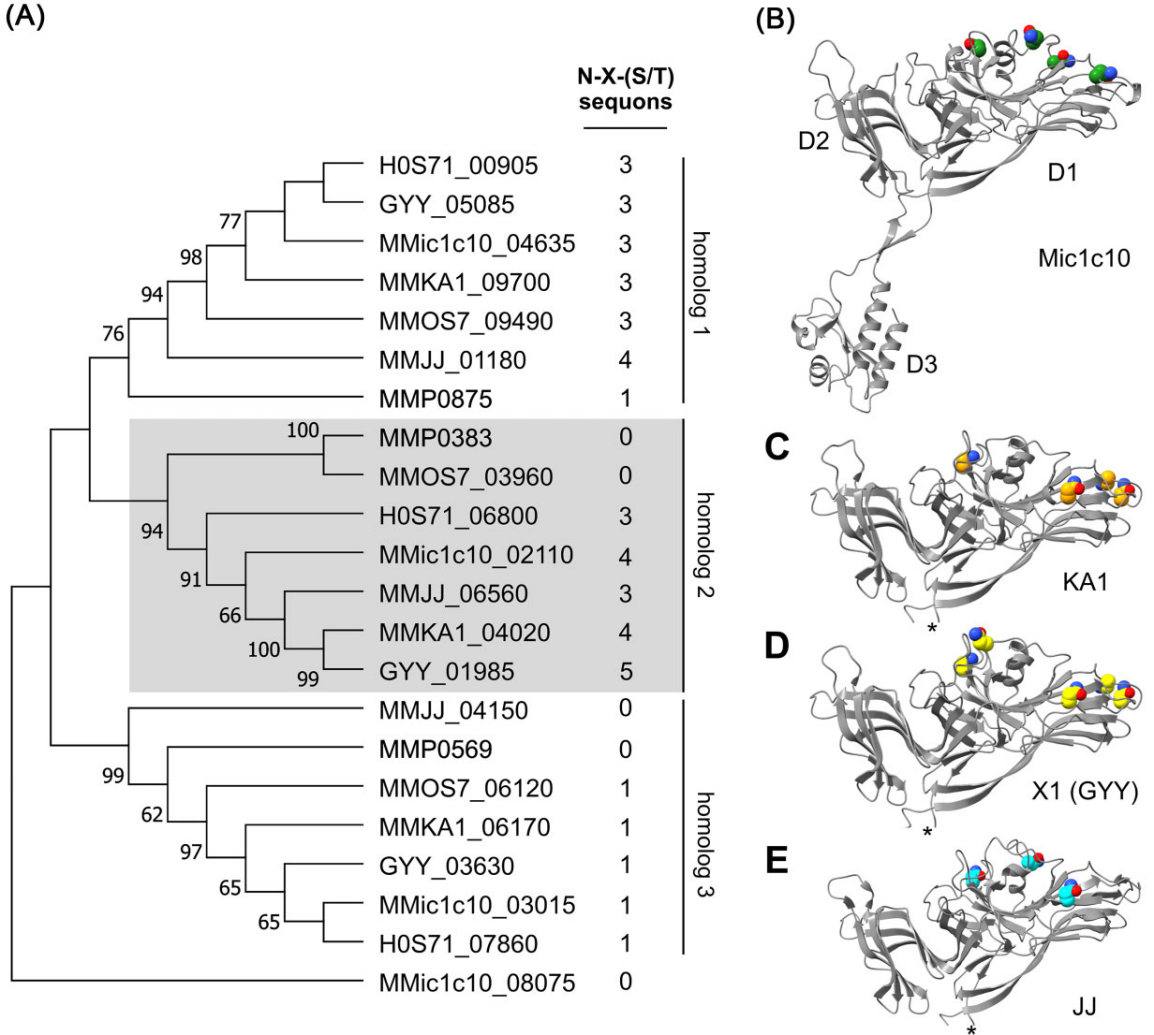
S-layer proteins in MIC methanogens show variations in possible N-glycosylation sites. (**A**) Phylogenetic tree of three homologs of S-layer proteins found in corrosive and non-corrosive *M. maripaludis* strains along with their number of N-X-(S/T) sequons as possible N-glycosylation sites (threshold of 32%, Suppl. Table S7). MMic1c10_08 075 encodes for a protein containing only D3 of the S-layer proteins and is used as an outgroup. See Suppl. Figs 8–10 and Table S7 for further details. (**B–E**) AF structural models of S-layer homolog 2 proteins for the indicated strains showing the three-domain (D1-3) architecture along with depicting asparagine residues from N-X-(S/T) sequons. *The highly conserved domain D3 has been omitted from strains KA1, X1 and JJ for clarity. Asparagine residues of N-X-(S/T) sequons are shown as spheres with nitrogen blue, oxygen red, and the carbon atoms color coded based on strain. Protein homologs are listed in (**A**) with corresponding locus tag identifiers [Mic1c10 (MMic1c10), KA1 (MMKA1), OS7 (MMOS7), MIC098Bin5 (H0S71), X1 (GYY), JJ (MMJJ), and S2 (MMP)] and the tree inferred with MEGA11 using the maximum likelihood statistical method with 500 bootstrap replications. Bootstrap values are shown above branches with a cutoff of 50%.

Each of the genomes of the seven analyzed strains of *M. maripaludis* encodes three S-layer proteins (Fig. 4 and Suppl. Figs. S8-10). As these are important for the contact of archaeal cells with their environment and are often glycosylated (Albers and Meyer 2011), they were further analyzed. A special focus was on the S-layer homolog 2 (MMic1c10_02 110), encoded 16 kb downstream of the glycan biosynthesis cluster 1 (Fig. 3A). Genomic comparison with KA1 and OS7 showed a divSEG within a central region of the gene flanked by regions of high conservation (Suppl. Table S6). Strikingly, comparing the encoded S-layer protein homolog 2 across the seven strains of interest shows high divergence in a portion of the sequence beginning after the first 100 residues, while the N- and C-termini are largely conserved (Suppl. Fig. S8B). This high divergence is generally not observed in the two other annotated S-layer homologs (Suppl. Figs. S9 and S10). Nevertheless, protein modelling by AlphaFold (AF) predicts the three homologs to be structurally similar (Suppl. Fig. S8A). AF predicts a three-domain architecture for homolog 2 of Mic1c10 with domain 1 and 2 (D1 and D2) containing high β-strand content (Fig. 4B). The divSEQ across homolog 2 proteins maps on to these two domains (Fig. 4B-E and Suppl. Fig. S8B).

Bioinformatic analysis indicated further that potential N-glycosylation at N-X-(S/T) sequons varies across the S-layer homologs (Fig. 4A and Suppl. Table S7) (Gupta and Brunak 2002). The overall number of sequons (but not their position) was previously reported (Kawaichi et al. 2024). Homolog 2 showed the most pronounced variation in number and positioning of sequons with strains OS7 and S2 not containing any sites and X1 having five sites. Potential N-glycosylation sites were localized to one face of highly divergent D1 with each protein having a unique N-X-(S/T) sequon fingerprint (Fig. 4B-E, Suppl. Table S7). All sequons in homolog 2 of Mic1c10 were in unique positions whereas X1 and KA1 had four matching sequons. The presence of highly divergent S-layer proteins in closely related strains and species is not uncommon in archaea as demonstrated for *Haloquadratum walsbyi* and *Haloferax volcanii* (Dyall-Smith et al. 2011, Tittes et al. 2021). A divergent S-layer with alterations in glycan composition most likely reflects the variable environments in which these organisms are exposed to (Vershinin et al. 2024). In MIC-causing methanogens, it is predicted therefore that variations documented of the glycan biosynthesis clusters in a genomic region near a divergent S-layer protein influences how these methanogens interact with their environment, *e.g*. biofilm formation, cell-cell or cell-substrate contact.

### MIC core analysis

**Figure 5.**
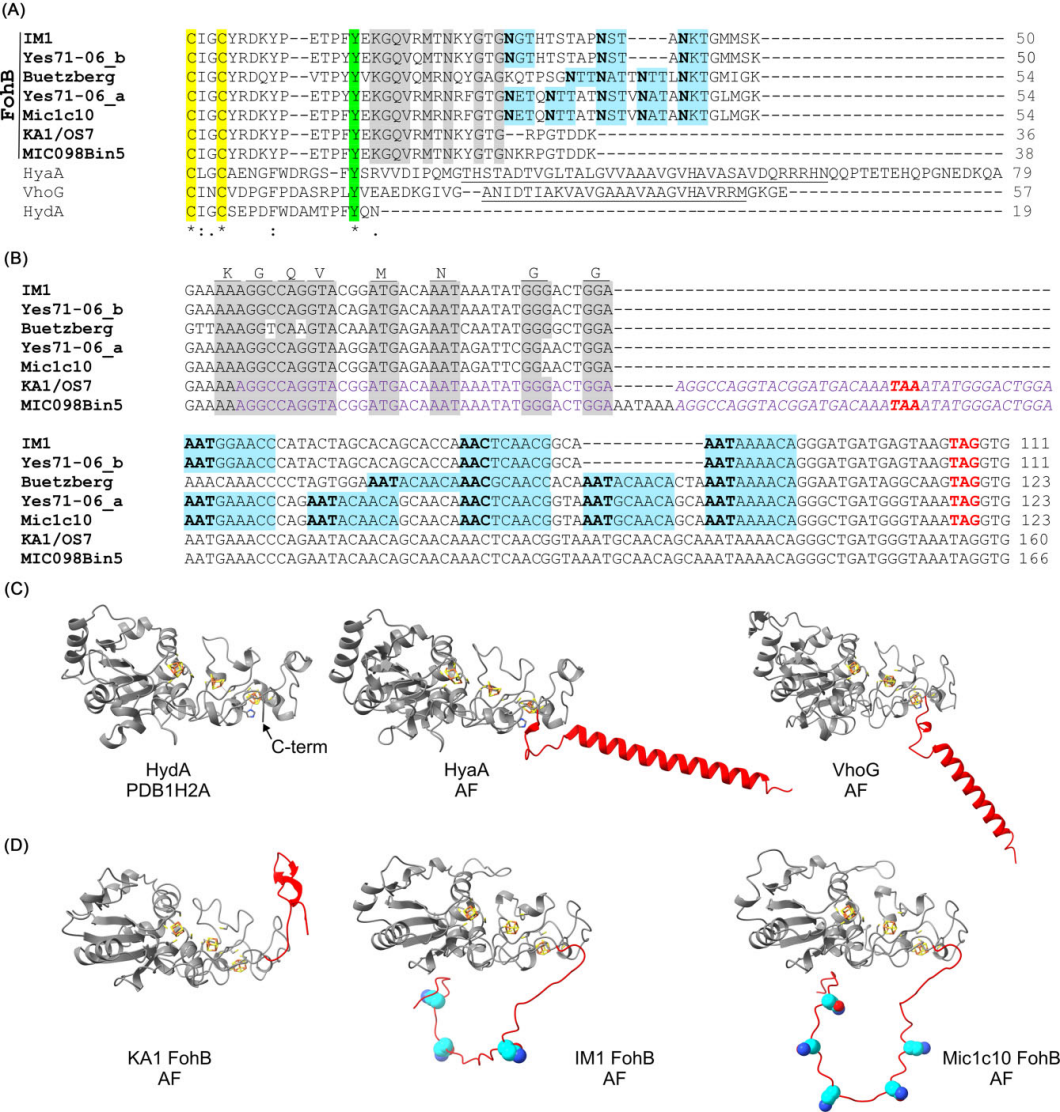
The C-terminal region of FohB is unique compared to other TAT-secreted NiFe-hydrogenase SSUs. (**A**) Protein sequence alignment of TAT-secreted SSUs with FohB in bold for indicated strains/isolates. Yes71-06 contains two copies. Possible glycosylation sequons (N-X-(S/T)) are shown in cyan with the Asn residues in bold. α-helical transmembrane domains are underlined. The last two cysteine residues of the distal Fe/S binding site are shown in yellow. A highly conserved tyrosine residue is in green. Conservation in the C-terminal region of MIC hydrogenases is shown in grey. (**B**) DNA sequence alignment of FohB’s corresponding to (**A**). A frameshifting duplication in KA1/OS7 and MIC098Bin5 is shown in purple with the duplication italicized. Stop codons are colored red. This duplication introduces an out-of-frame stop codon so that the N-X-(S/T) sequons, even though encoded in the genome, are not translated. (**C–D**) Structural models of TAT-secreted NiFe-hydrogenase SSUs with varying C-terminal extensions (colored red). Proximal, medial, and distal Fe/S clusters are shown as yellow and orange sticks. For the AF models, Fe/S clusters were modelled in based on alignment with similar proteins. Periplasmic and pseudo-periplasmic localized proteins (**C**) show differences to the corrosion-correlated FohB proteins (**D**). The C-termini of FohB proteins are hydrophilic and in the case of IM1 and Mic1c10, are proposed to be N-glycosylated (colored spheres: carbon, cyan; oxygen, red; nitrogen, blue). UniProt identifiers; HydA (P21853, *Desulfovibrio vulgaris* Miyazaki F); HyaA (P69739, *E. coli*); VhoG (Q50248, *Methanosarcina mazei*). Structural models have TAT-signal peptides removed. AF, AlphaFold.

The ability to corrode metal surfaces has been attributed to the presence of the MIC hydrogenase (FohAB) nestled within the MIC island in *M. maripaludis* (Tsurumaru et al. 2018, Lahme et al. 2021, Kawaichi et al. 2024). Furthermore, it has been proposed that N-glycosylation of FohB may influence the localization and hence corrosivity of MIC hydrogenases (Kawaichi et al. 2024). The FohB subunit binds three iron-sulfur (Fe/S) clusters important for shuttling electrons to the [Ni-Fe] active site of FohA (Shafaat et al. 2013). To better understand FohB, a bioinformatic comparison was carried out with known SSUs of other TAT-secreted NiFe-hydrogenases which are either soluble or anchored to the membrane via a C-terminal α-helix (Fig. 5). Protein alignment focusing on the sequence spanning from the distal Fe/S cluster cofactor to the C-terminus indicated proteins of various C-terminal compositions (Fig. 5A). After a conserved tyrosine, FohB diverges from three other SSUs (HydA, HyaA, and VhoG). FohB from Mic1c10 and Yes71-06a contain five N-X-(S/T) sequons for potential N-glycosylation making the C-terminus hydrophilic and differentiating it from the hydrophobic α-helix found in other hydrogenases. Distinguishing Mic1c10 from the other *M. maripaludis* strains is the fact that OS7, KA1, and MIC098Bin5 all lack potential N-glycosylation sites due to a 37 bp duplication event leading to a frameshift mutation upstream of the N-X-(S/T) sequons that results in a premature stop codon which is out-of-frame in Mic1c10 (Fig. 5A-B). In strains IM1 and Buetzberg, potential N-glycosylation sites are present but with fewer sites (three and four sites, respectively). Notably, MIC neighbourhood 1 in all *M. maripaludis* strains is ∼15 kb upstream of the second glycan biosynthesis cluster (Fig. 1). In a similar manner, the MIC neighbourhood 2 is directly upstream of a glycan biosynthesis cluster containing genes for pseudaminic acid in strain Buetzberg (Tejerizo et al. 2017). Consistent with the hypothesis that glycosylation of FohB localizes the MIC hydrogenase in the cell wall, filtrates from IM1 and Mic1c10 did not produce significant amounts of hydrogen when exposed to iron as compared to abiotic controls and *M. maripaludis* S2 (Suppl. Fig. S11 and Suppl. Text S10). These results are comparable to previous findings. However, it is important to note that variations in experimental conditions—such as the use of different media, filtrate concentrations, or incubation times—may have influenced the results (Holmes et al. 2024, Kawaichi et al. 2024). Therefore, a hydrophilic C-terminus of FohB with potential N-glycosylation sites is one of the defining features of the MIC hydrogenase family. Yet some enzymes have lost this characteristic due to mutations, which may have an influence on strain corrosiveness.

**Figure 6.**
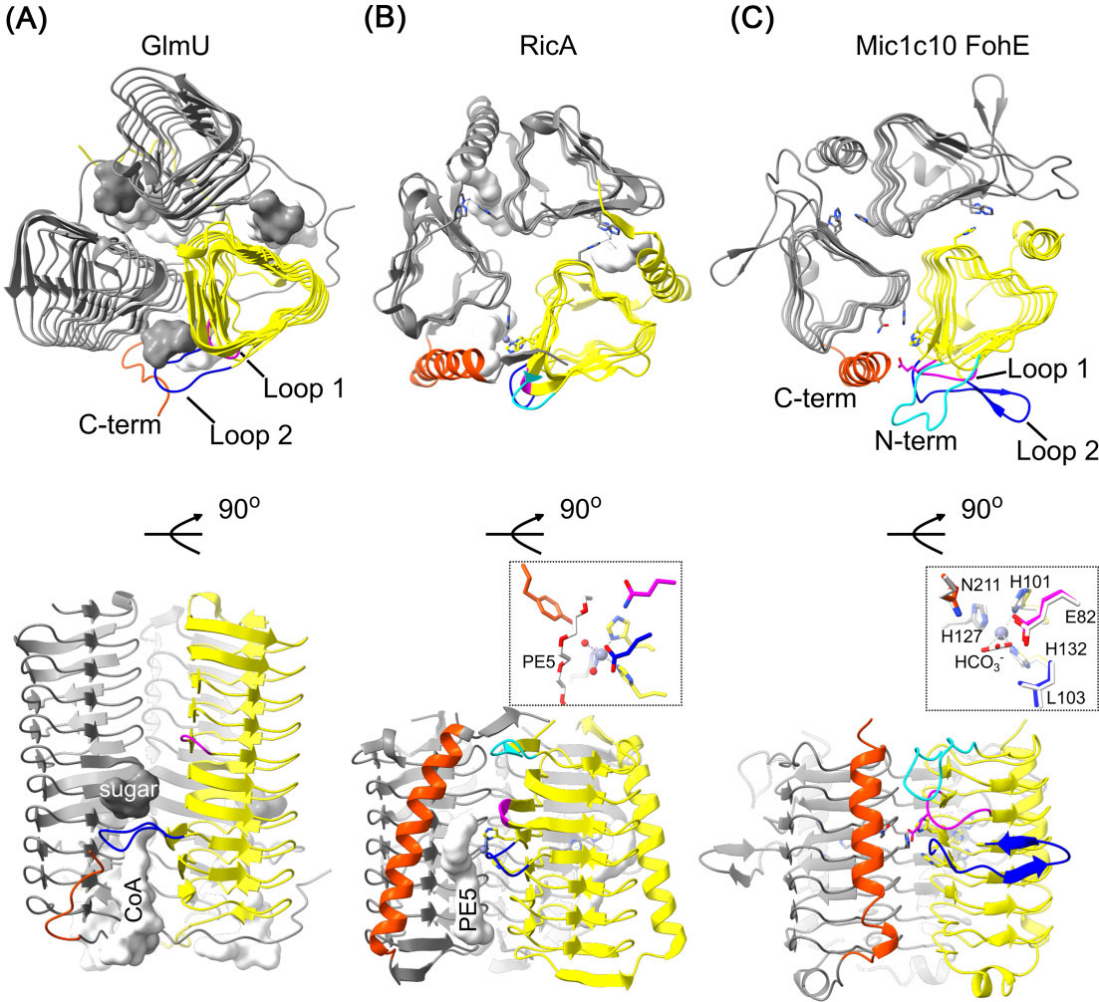
Structural comparison of FohE from the MIC core to other β-helix proteins. (**A–C**) Homotrimeric protein structures of the β-helix proteins GlmU (**A**, PDB 2OI6), RicA (**B**, PDB 4N27), and Mic1c10 FohE (**C**, AF model) in two orientations with one protomer colored yellow. Proteins of this superfamily often contain two substrate binding sites as seen for GlmU binding the GlcN-1-PO_4_ substrate (sugar) and coenzyme A (CoA). Substrates can be orientated in the binding pockets via several loops (N-terminal, cyan; Loop 1, magenta; Loop 2, blue) and the C-terminus (orange). The substrate for RicA is not known, but a hydrophilic polyethylene glycol 400 (PE5) molecule can bind. The γ-carbonic anhydrase-like subfamily of β-helix proteins (**B** and **C**) can further bind divalent metal ions at a 3His metal coordination site (insets, Zn^2+^ grey sphere). Overlay of the AF model of FohE with γ-carbonic anhydrase Cam predicts a nearly identical metal coordination environment and substrate binding pocket (inset of **C**, PDB 1QRL in white, see also Suppl. Fig. 12A). Key residues are labeled for FohE along with a bicarbonate ion (HCO_3_^−^) in the substrate binding pocket of Cam. Note: N-termini have been removed for clarity from GlmU (UDP-GlcNAc binding domain) and FohE (signal peptide, residues Met1-Ala33).

Along with the presence of the genes for a TAT system responsible for secreting FohAB, the sixth gene of the MIC core, FohE, remains present in all nine MIC cores across different genomic neighbourhoods (Fig. 2). Bioinformatic analysis confirms FohE to be a member of the left-handed β-helix superfamily of proteins. The β-helix tertiary structure is formed by repeating β-strands containing six residues and forms trimeric protein complexes that can generally bind various substrates in two solvent exposed binding pockets at the dimer interface (Fig. 6, Suppl. Fig. S12, Suppl. Text S11) (Jenkins et al. 1998, Prabha and Balaji 2021). These include acyltransferases involved in nucleotide-sugar biosynthesis (*e.g*. GlmU, Agl17, LegH-like, Fig. 3) and metalloproteins involved in the hydration of CO_2_ and other putative acyltransferases (e.g. γ-carbonic anhydrase CAM, PaaY, and RicA, Fig. 6B-C and Suppl. Fig. S12).

Like Cam, FohE is predicted to be localized extracellularly as it contains a putative SEC signal peptide (Supp. Fig. 13A). AF predicts FohE to form a β-helix that can be modelled as a trimer (Fig. 6C). The trimeric FohE model and its 3-His metal binding site most closely aligns to Cam (PDB 1QRL) and contains Loops1-2 that are expected to mediate substrate binding, protein partner binding, and/or catalysis (Fig. 6C and Suppl. Fig. S12A). The conserved glutamate E82 of FohE in Loop1 is positioned close to the active site and could facilitate the coordination of a carbonyl moiety as seen in Cam (Fig. 6C). This glutamate is within a conserved N-terminal half of the β-helix whereas the C-terminal half of FohE diverges from Cam (Suppl. Fig. S13A). The divergence begins with Loop2, where Cam has a longer Loop2. Interestingly, the protein in IM1 is truncated exactly at this divergence point but still contains Loops1-2. As a result, FohE in IM1 contains only two out of the three histidines for metal coordination (Suppl. Fig. S12D). This truncation is the result of a point mutation that has also been identified in the MIC cores of Yes71-06b and Yes49-02, thereby underscoring the biological significance (Suppl. Fig. S13B-C). A 3’ segment of *fohE*, given here the name *fohE’*, is also annotated as an ORF in the MIC transposons of the two Yes isolates and found also highly conserved in IM1 (Fig. 2A). A canonical start codon is not evident, but it might be speculated that one of several atypical start codons (e.g. ATA) is used (Suppl. Fig. S13B-C and Suppl. Text S11). Depending on where the translational start site occurs for FohE’, a heterodimer of FohE/FohE’ may or may not lose the ability to bind metals at the 3-His coordination site (Suppl. Fig. S12D). Therefore, in these *Methanobacterium* strains and isolates, the two proteins FohE/E’ might be encoded, each corresponding to the two binding pockets known for the β-helix family. Due to the structural similarity with known β-helix family members involved in glycan biosynthesis that have two substrate binding pockets, the conserved genetic location within the MIC core that also encodes putatively glycosylated FohB, and the presence of FohE/E’ in some organisms potentially lacking a metal-binding site, we assign FohE as a potentially bifunctional enzyme required for the proper localization and/or function of FohAB in the cell wall (see also Suppl. Text S11).

### Microscopic analysis of methanogens on surfaces

**Figure 7.**
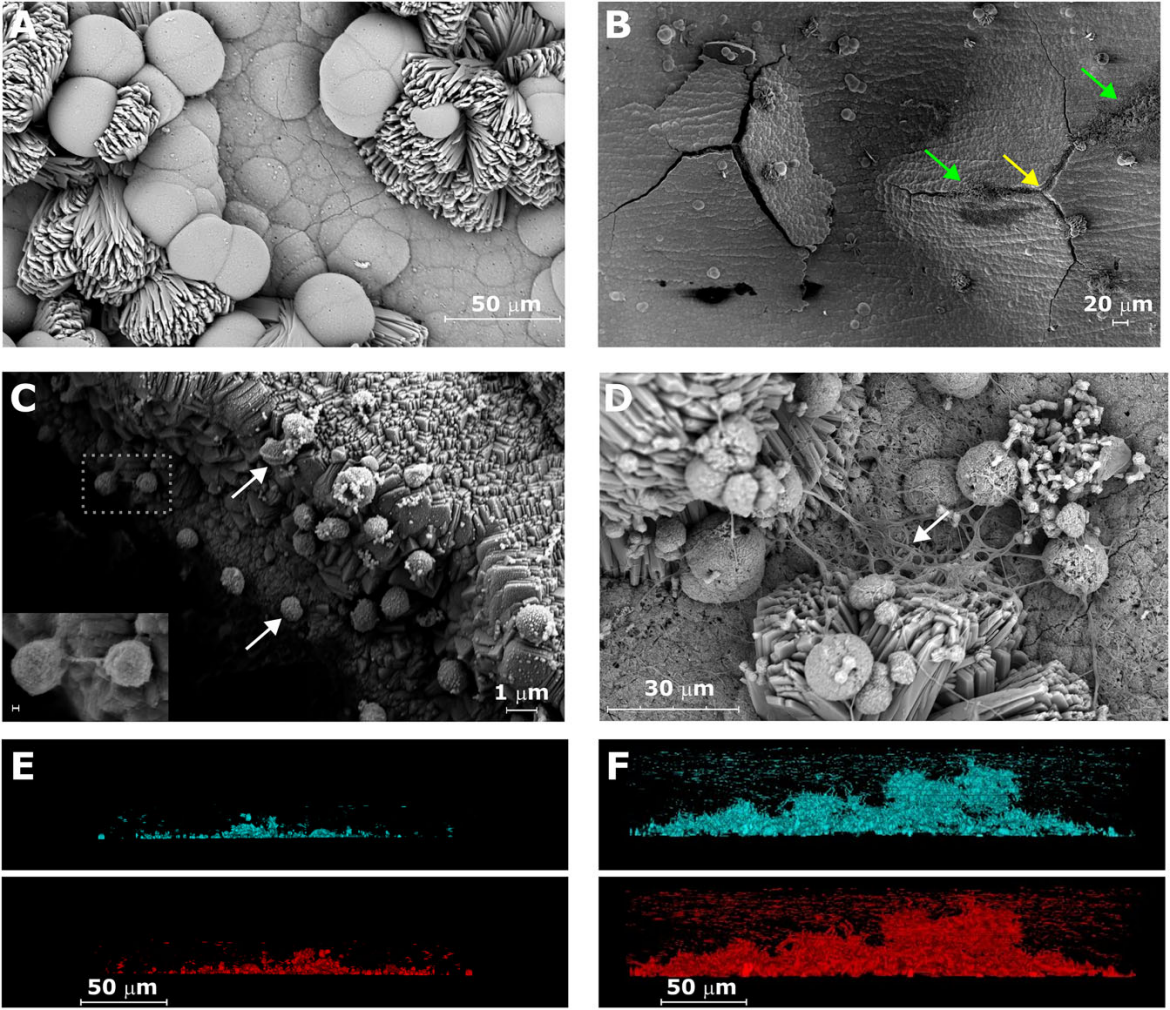
Microscopic analysis of *Methanobacterium* IM1 and *M. maripaludis* Mic1c10. **(A–C)** SEM images of either highly **(A)** or moderately **(B)** corroded iron coupons that had been incubated in static cultures of Mic1c10. The yellow arrow marks a crevice in the corrosion layer, which is shown at higher magnification in **(C)**. Green arrows mark an unknown material that was detected at the crevices. White arrows mark spherical particles identified as *M. maripaludis* cells interacting with the walls of the crevice **(C)**. The inset in **(C)** is an enlargement of the dashed box area, scale bar is 100 nm. **(D)** SEM image of a highly corroded iron coupon incubated in a static culture of IM1. The white arrow marks biofilm formation on corrosion products. **(E–F)** CLSM images of Mic1c10 **(E)** and IM1 **(F)** grown on glass surfaces. Top images represent F_420_ autofluorescence (cyan) and bottom images fluorescence of the protein stain SyproOrange^®^ (red).

The rate of Fe^0^ oxidation (equation 2) promoted by the heterologous expression of the MIC core has previously been correlated to surface-associated growth of a *M. maripaludis* JJ mutant (Holten et al. 2021). To determine if and how Mic1c10 can interact with surfaces, its growth behavior on iron coupons and glass was assessed and compared to IM1. Stock cultures of Mic1c10 and IM1 grown statically resulted in the conglomeration of Fe^0^ granules along with the formation of a black precipitate on the surface of the granules (Suppl. Fig. S14). Non-corrosive JJ and corrosive KA1 did not demonstrate the conglomeration behavior. Growth of Mic1c10 under static conditions on iron coupons caused moderate to extensive corrosion, yet cells were not found associated to the surface of corrosion products by scanning electron microscopic (SEM) analysis (Fig. 7A-B). The existence of an unknown substance protruding from some crevices on a moderately corroded coupon, however, drew our attention to these features. On the walls and edges of these crevices, coccoid particles were identified as *M. maripaludis* cells (Fig. 7C) (Jarrell et al. 2013, Chen et al. 2021). Cells were generally not directly in contact with one another, and extracellular polymeric substance (EPS) could not be discerned in their vicinity. Few cells, however, appeared to be in direct contact via cellular appendages (Fig. 7C, inset). In contrast, *Methanobacterium* IM1 grew extensive biofilms on corrosion products with web-like structures and presence of EPS (Fig. 7D). Elongated cells were observed connecting various types of corrosion products, from the corrosion surface to corrosion protrusions. IM1 also grew denser biofilms on a glass substrate than Mic1c10 as visualized by confocal light scanning microscopy (CLSM) (Fig. 7E-F). Overall, Mic1c10 and IM1 show distinct differences in how they interact with surfaces.

## Discussion

As MIC on metallic infrastructure is a world-wide threat causing billions of US dollars in damage (Camara et al. 2022), new perspectives are required to better understand the underlying mechanisms. The continued dependence on metallic infrastructure in addition to the transition to a H_2_ economy utilizing existing and new steel pipelines forebode that this threat will remain relevant into the future (Jemblie et al. 2025). With the identification of the *fohAB* genes and their correlation to the oxidation of iron, Tsurumaru *et al*. in 2018 provided the first direct genetic evidence for describing how methanogens contribute to MIC (Tsurumaru et al. 2018). The presence of genes encoding the MIC hydrogenase remains the only validated experimental evidence mechanistically linking methanogens to the corrosion of metal (Tsurumaru et al. 2018, Holten et al. 2021, Lahme et al. 2021, Kawaichi et al. 2024, Lahme and Aguas 2025). Our study, focusing on Mic1c10 and IM1, provides a detailed genomic comparison of methanogens involved in Mi-MIC and confirms the spread of the MIC hydrogenase FohAB encoded in the newly defined MIC core amongst methanogens at different taxonomical levels. Together with the identification of *fohA*/FohA in oil facilities around the world (Tsurumaru et al. 2018, Lahme et al. 2021, Lahme and Aguas 2025), our results indicate that mesophilic and thermophilic organisms in marine sediment and groundwater ecosystems can also carry genetic traits conducive to biocorrosion.

**Figure 8.**
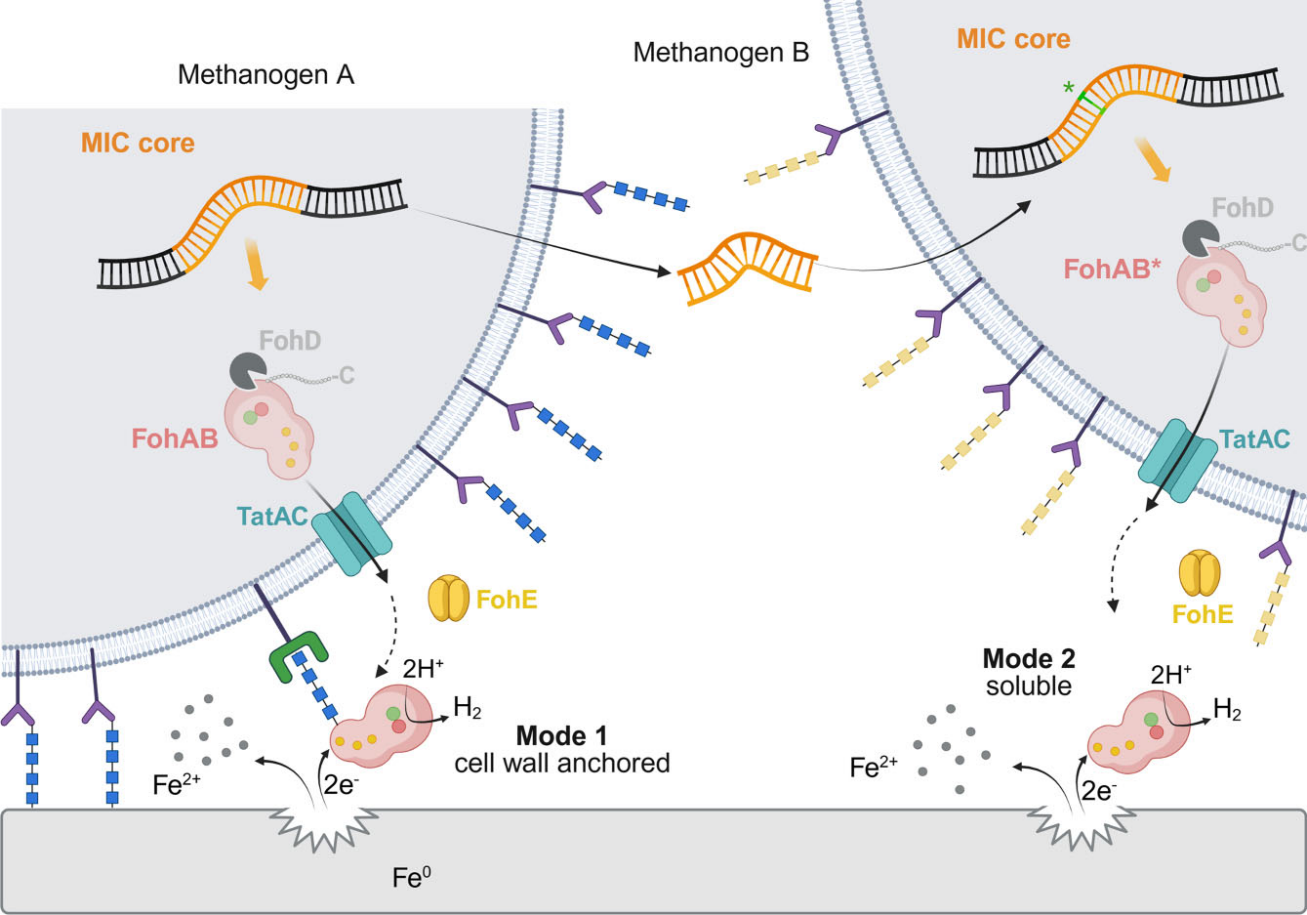
Two putative modes of action for MIC hydrogenases (FohAB) involved in biocorrosion. The MIC core (orange) encodes six core proteins: the secreted NiFe-hydrogenase FohAB, hydrogenase maturation protease FohD, a TAT secretion system TatAC, and a secreted, γ-carbonic anhydrase-like protein of unknown function FohE. The MIC core can be mobilized via various means and passed from methanogen to methanogen (A to B, see Fig. 2). Methanogen A represents organisms encoding FohB proteins that can be putatively glycosylated at the C-terminus (blue squares), which may anchor the hydrogenase to the cell wall via interaction with glycan-binding proteins (Kawaichi et al. 2024). Mode 1 therefore predicts close association of cells and/or biofilms to iron surfaces, permitting the abstraction of electrons used to catalyze hydrogen formation by FohAB and consequently the solubilization of Fe^2+^ facilitating the corrosion process. Adherence of cells to iron may further be supported by glycans attached to S-layer proteins (purple) (Holten et al. 2021). In the second mode depicted for methanogen B, mutation of the MIC core (green asterisk) leads to the loss of glycosylation sites in FohB (FohB*) (Tsurumaru et al. 2018). Without an affinity to the cell wall, FohAB* may diffuse to iron surfaces and carry out catalysis without the requirement of cells being attached to the surface. Glycans, or the lack thereof, on S-layer proteins can vary in number and from strain to strain in *M. maripaludis* (yellow squares in methanogen B). MIC island mobilization and the two possible modes of action for FohAB also apply in *Methanobacteria*, yet with the S-layer replaced by a rigid archaeal peptidoglycan layer. Image made with Biorender, https://BioRender.com/n9co47a.

By exploring the genetic diversity of MIC-causing methanogens, it has been discovered that the MIC core can be found in at least two different genetic neighborhoods. It remains to be determined if and how the auxiliary genes of the two neighborhoods impact corrosion, yet the commonality of the MIC core suggests that they are the minimal set of genes required for corrosion to occur. Heterologous expression of the MIC core on a plasmid in a mutant of the non-corrosive *M. maripaludis* JJ was previously shown to enhance Fe^0^ oxidation (Holten et al. 2021). This further supports that the MIC core is responsible for Mi-MIC (Holten et al. 2021). Three different mechanisms of mobilization leading to the transmission of the MIC core between cells can be described (Figs 2 and 8). Expanding upon the initial discovery that MIC neighborhood 1 in *M. maripaludis* OS7 is genetically unstable (Tsurumaru et al. 2018), flanking direct repeats allow for the MIC island of Mic1c10 to be excised when grown without selective pressure of iron as the sole electron donor. Transmission of the excised MIC island could occur between any strain containing *ycaO*, which occurs in most methanogens. A second means of mobilization via horizontal gene transfer predicts the origin of MIC neighborhood 1 in *M. maripaludis*. Two nine-nucleotide motifs enclosing MIC neighborhood 1 in *Methanobacterium* isolate Yes71-06 are also present in the *ycaO* gene, slightly more than 200 bp apart. The hypothesized excision of this DNA fragment and integration into a *M. maripaludis* genome explains the origin of the direct repeats flanking the MIC island. Identifying MIC neighborhood 2 in IM1 that is also found in isolates of other *Methanobacteria* led to uncovering the third mechanism of mobilization via transposases. With multiple mechanisms of mobilization, it may be expected that the MIC core will spread further and thus will continue to be discovered in metagenomic studies or in other putatively corrosive methanogens. Continued studies and metagenomic sampling will also help in further tracking the evolutionary origin of the MIC proteins.

Setting Mic1c10 apart from the other corrosive strains OS7, KA1, and MIC098Bin5, is the fact that FohB in Mic1c10 is predicted to be glycosylated in a similar manner to FohB found in MIC neighborhood 1 of *Methanobacterium* isolate Yes71-06. This observation indicates that Mic1c10 is a closer ancestor to *Methanobacteria* containing MIC neighborhood 1 and that strains OS7, KA1, and MIC098Bin5 evolved from a Mic1c10-like strain. An important evolutionary step was the duplication of a 37 bp segment at the 3’-end of *fohB*, causing C-terminal truncation of FohB and prohibiting glycosylation. FohB of the three strains OS7, KA1, and MIC098Bin5 are mutated in the same way and thus all three cannot be glycosylated. The fact that the MIC island in *M. maripaludis* has a low read coverage in sequencing efforts and some strains have evolved to secrete a FohAB enzyme that is either no longer associated to the cell wall or less stable, suggests that it is an evolutionary advantage for this organism to not be tightly associated with the function of FohAB. On the other hand, the MIC transposon in *Methanobacterium* IM1 does not contain direct repeats and is expected to be stably integrated. IM1 grows slowly on H_2_ as a sole electron donor indicating the central role FohAB must play in abstracting energy equivalents from iron for driving methanogenesis in this strain (Dinh et al. 2004).

The information gleaned from comparing the MIC-related genes in addition to the inventories of S-layer proteins and glycan biosynthesis gene clusters along with the adherence of cells to corrosion products facilitates the comparison of two putative modes of action for Mi-MIC (Fig. 8). While both require the interaction of secreted FohAB with iron surfaces to facilitate the evolution of H_2_, they are differentiated by the localization of the enzyme. Both modes require the cleavage of the C-terminus of FohA by FohD and secretion by TatAC. In mode 1, FohB promotes the anchoring of the MIC hydrogenase to the cell wall of methanogens via glycosylation or other biomolecular interactions (Fig. 8) (Kawaichi et al. 2024). Mode 2 involves FohB proteins that are predicted to not be tightly associated with the cell wall and the MIC hydrogenase may diffuse away from the cell to iron surfaces (Tsurumaru et al. 2018). The levels of H_2_ evolution in spent media reflect these modes, with only mode 2 explaining increased H_2_ concentrations in spent filtrates of OS7 cultures exposed to iron (Tsurumaru et al. 2018). Based on these measures, FohAB in IM1 and Mic1c10 studied here are predicted to act via mode 1. Complete secretion of glycosylated FohAB to the extracellular milieu followed by adherence to metal surfaces via glycans, however, cannot yet be ruled out at this time. Along this line, it will be interesting to compare how glycosylation of FohB influences interaction of the MIC hydrogenase with metal surfaces along with its activity and stability as compared to non-glycosylated counterparts. Since glycosylation of S-layer proteins, Type IV pili proteins and archeallins can influence surface- and cell to cell interaction (Eichler 2013, Holten et al. 2021, Schulze et al. 2021), a similar scenario may be relevant for FohAB, which could influence corrosivity. Visualization of initial methanogenic biofilms on Fe^0^ surfaces, localization of the MIC hydrogenase, and positive controls with purified FohAB are required to further validate each mode of action across the corrosive methanogens encoding a MIC hydrogenase. Deciphering between the two is an important consideration in designing new mitigation strategies against corrosive methanogens.

As a MIC core component, FohE is expected to be important in both modes. Based on literature precedent and bioinformatic analysis, it is surmised here that FohE plays a role in the localization of the MIC hydrogenase by acting on the C-terminus of FohB in addition to acting as a potential carbonic anhydrase. Carbonic anhydrase activity may not be the only function, as the protein is predicted to be split in two (FohE/FohE’) in several *Methanobacteria* with the potential loss of a metal-binding residue preventing high-affinity metal binding required for binding CO_2_. The retainment of FohE in *M. maripaludis* strains with truncated FohB proteins may also assist in localizing the MIC hydrogenase to the cell wall, yet with lower affinity.

The affinity of cells for iron surfaces further informs about the two modes of action (Fig. 8), as suggested by previous work (Holten et al. 2021). In static cultures, Mic1c10 and IM1, but not KA1, promote the conglomeration of iron granules. While KA1 also exhibits some affinity for iron surfaces, it is expected to be lower than that of Mic1c10 (Uchiyama et al. 2010). The conglomeration may be due to the formation of biofilms at the metal-metal or metal-glass interface. Cells of Mic1c10, however, could only be identified in crevices of the corrosion layer and not on exposed surfaces. These observations suggest that Mic1c10 cells may become embedded within corrosion products over time (An et al. 2021), in contrast to IM1 where vast amounts of cells and EPS were visible on corrosion surfaces. Therefore, the accumulation of iron granules in Mic1c10 static cultures may alternatively be explained by an encapsulating corrosion layer. At present, there is no direct evidence to support or refute biofilm formation by Mic1c10 on Fe⁰ surfaces or within the corrosion products. Strains JJ and S2 may also interact with metallic surfaces (Chen et al. 2021, Holten et al. 2021), but require an electron donor other than Fe° for sustained growth. The highly divergent S-layer protein in *M. maripaludis* strains described here containing unique N-X-(S/T) sequon fingerprints (homolog 2) and the variations in glycan composition are likely to dictate how the cells interact with their environment including other cells and iron surfaces (Fig. 8). Notably, Mic1c10 contains four unique sequons in S-layer homolog 2 and was the only strain containing genes for rhamnose and mannose biosynthesis, which correlates to the presence of these sugars in the pseudomurein of *Methanobacteria* that can self-aggregate (Veiga et al. 1997, Kosaka et al. 2014). In contrast, OS7 has lost all potential glycosylation sites in S-layer homolog 2 and the glycan biosynthesis machinery in cluster 1. Variations in the CRISPR-Cas systems and the presence of integrative plasmids indicate that viral interactions may have played a role in generating the genetic variation of the *M. maripaludis* strains and in turn affected corrosivity (Sanchez-Nieves et al. 2023).

Considering this work in light of our prior corrosion studies (An et al. 2020, An et al. 2021), the higher the propensity of corrosive methanogens to adhere to iron surfaces, the greater the level of corrosion due to proximity and localization of FohAB with the iron surface. This conclusion is in agreement with the observation that the deletion of *aglB*, which prevents surface adherence induced by N-linked glycosylation, results in decreased oxidation of iron mediated by FohAB (Holten et al. 2021). Whereas both Mic1c10 and IM1 promote corrosion processes, only the latter successfully colonizes and forms extensive biofilms on the surface of corrosion products. Based on this comparison, it is expected that the cell wall or sacculus of *Methanobacteria*, involving archaeal-type peptidoglycan (Mukhopadhyay 2024), promotes attachment to iron surfaces to a greater degree than the S-layer of *Methanococci* allowing for higher rates of corrosion. Along with iron surfaces, cells can also be observed on graphite electrodes for IM1 but not Mic1c10/KA1 even though all can perform electromethanogenesis presumably via the availability of H_2_ produced by FohAB (Beese-Vasbender et al. 2015, Mayer et al. 2022). Dynamic or agitated culture conditions in corrosion and electromethanogenesis studies yet can have a strong influence on the localization of cells at surfaces and biofilm formation as compared to static conditions (Beese-Vasbender et al. 2015, An et al. 2020, An et al. 2021, Holten et al. 2021, Mayer et al. 2022).

This work further elucidates the threat posed by Mi-MIC, in which methanogens can pass genetic elements confirmed to induce corrosion, the MIC core, via multiple means of DNA mobilization to other methanogens (Fig. 8). Whereas the MIC hydrogenase encoded within the MIC core can be attributed to the molecular species inducing corrosion via two possible modes of action, it is shown that the ability of methanogens to adhere to iron surfaces plays an important role in the degree of biocorrosion. With the detailed genomic perspectives presented here, future gene deletion studies can begin dissecting the roles in which various MIC-related genes play in biocorrosion involving *Methanobacteria* and *M. maripaludis*. Additionally, localization of FohAB with respect to iron surfaces and the cell wall will be an important aspect to further clarify. The identification of the MIC core in metagenomic data also argues for the continued development of monitoring capabilities (qPCR and immunosensors) for FohAB and other biomarkers in the environment is an important aspect to pursue in order to properly access current and future threats to steel and metallic infrastructure around the world.

## Author contributions

S.K.: conceptualization, investigation, validation, formal analysis, visualization, writing original draft, review & editing; J.J.B.: conceptualization, funding acquisition, validation, formal analysis, investigation, visualization, writing original draft, review & editing; F.P.: data curation, formal analysis, investigation, validation, visualization, writing original draft, review & editing; M.D-S.: investigation, validation, visualization, writing original draft, review & editing; K.S.: investigation, validation; G.A.E.: conceptualization, investigation, visualization, validation, review & editing; A.K.: conceptualization, funding acquisition, project administration, resources, supervision, writing & editing.

## Supplementary Material

xtaf018_Supplemental_Files

## Data Availability

The Mic1c10 sequencing data, including raw reads, have been submitted as BioProject PRJNA1210997. The Mic1c10 genome is available under GenBank accession CP186018.
