# Supplementary material for: From genes to Black Rust: genomic insights into corrosive methanogens
Source: FEMS Microbes. 2025 Nov 17;6:xtaf018. doi: 10.1093/femsmc/xtaf018 (PMC12667266; doi:10.1093/femsmc/xtaf018)
Supplement: xtaf018_Supplemental_Files [file xtaf018_supplemental_files.zip › FEMSMC-2025-029.R1 one sentence summary.docx]

Genetic analysis indicated that a core set of genes correlated to biocorrosion has spread amongst certain archaea and the rate of corrosion is influenced by the composition of the cell wall.
