# Supplementary material for: From genes to Black Rust: genomic insights into corrosive methanogens
Source: FEMS Microbes. 2025 Nov 17;6:xtaf018. doi: 10.1093/femsmc/xtaf018 (PMC12667266; doi:10.1093/femsmc/xtaf018)
Supplement: xtaf018_Supplemental_Files [file xtaf018_supplemental_files.zip › fpf_MmarGnm_Supp_250624_FEMS-Microbes-Final-accepted.docx]

Supplementary Material for

**From Genes to Black Rust: Genomic insights into corrosive methanogens**

*Kleinbub et al.*

- **Supplementary Figures S1-14**
- **Supplementary Texts and Methods S1-11**
- **Supplementary Tables S1-11**

**1. Supplementary Figures**


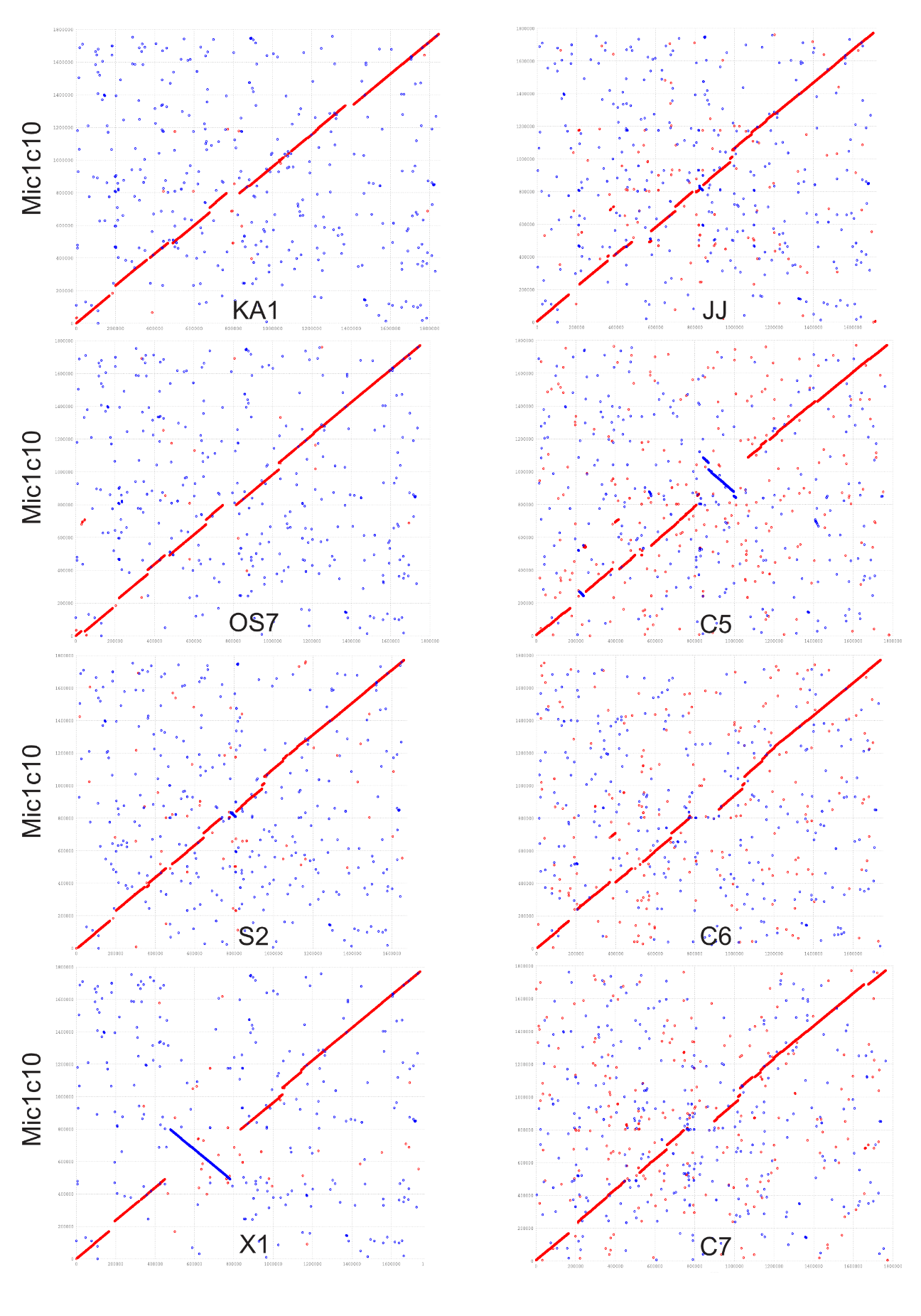


**Supplementary Figure S1: DNA dot plots (Mummer) comparing the *M. maripaludis* Mic1c10 genome with the indicated strains of the same species.** *M. maripaludis* Mic1c10 was compared to available complete genomes from the genus *Methanococcus* (see Suppl. Table S1). For each plot of pairwise similarity, the names of the strains are given on vertical and horizontal axes. Red plot lines represent similar sequences that both occur in the forward direction, whereas blue plot lines indicate similar sequences where one sequence is inverted. For ease of comparison, the following changes were made to comparison genomes; the *M. maripaludis* C5 genome (CP000609) was opened at nt 1628358 and reverse-complemented; the *M. maripaludis* C6 genome (CP000867) was opened at nt 896246 and reverse-complemented; the *M. maripaludis* C7 genome (CP000745) was opened at nt 965920; and the *M. maripaludis* JJ genome (CP026606) was opened at nt 1014793 and reverse-complemented.


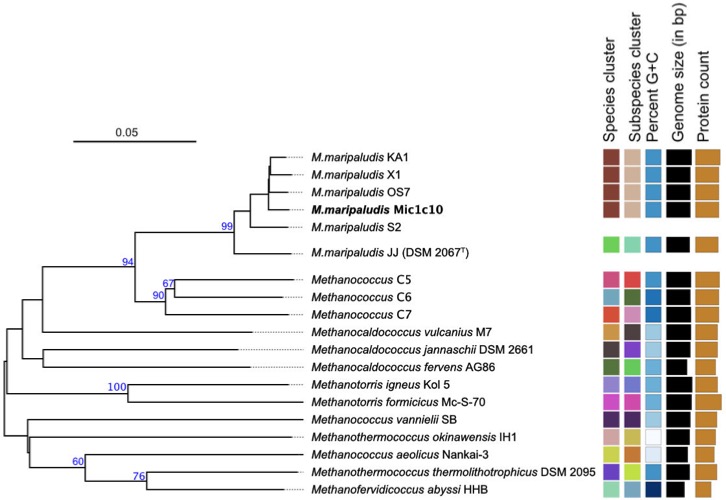


**Supplementary Figure S2: Phylogenetic tree reconstruction of *M. maripaludis* strains and related species using whole genome sequences.** Tree inferred with FastME 2.1.6.1 (Lefort *et al.*, 2015) from GBDP distances calculated from genome sequences. The branch lengths are scaled in terms of GBDP distance formula d5. The numbers above branches are GBDP pseudo-bootstrap support values > 60% from 100 replications. The average branch support over all nodes is 59.5%. The tree was rooted at the midpoint. Columns of coloured squares at right summarise properties of the named taxa in the tree at the same horizontal line.

**
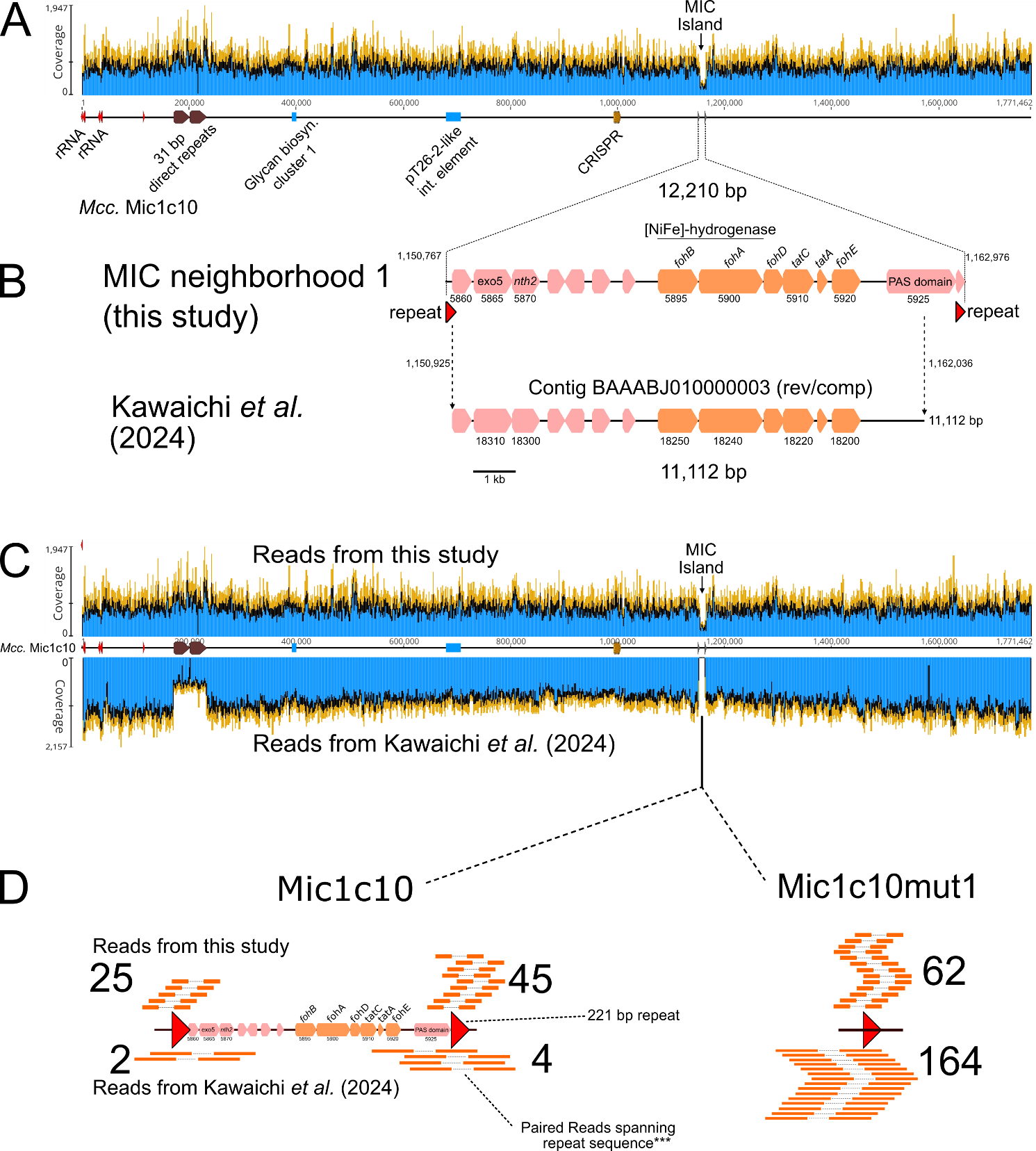
**

**Supplementary Figure** **S3:** **Comparison of read coverage across the MIC island of two *M. maripaludis* Mic1c10 genome projects.** **A**) Sequence coverage analysis for the complete genome sequencing project reported in this study. A total of 8 230 722 reads were mapped to the entire genome consisting of 1 771 462 bp using the map-to-reference tool within Geneious Prime ver. 2024.0.7 (Geneious mapper, medium-low sensitivity/fast, default settings). Read coverage is shown as the blue plot above the gene map and averages 664-fold outside of the MIC island. Across the MIC island (MIC neighborhood 1, colored as in Figure 2A), the average read coverage is 214-fold. Refer to Figure 1 for other features that have been labeled. **B**) Details of the MIC island of the two genome sequencing projects. The full MIC core island nestled in neighborhood 1 (top), including the two flanking near-identical copies of the 221 bp repeat (red arrows), has 12 210 bp. Protein-coding genes are indicated by arrows, indicating the encoding strand and drawn to scale. Relevant genes are labelled. Locus tag numbers of some genes (without the locus tag prefix MMic1c10) are given below the arrow. The partial MIC island reported by Kawaichi *et al.* (Kawaichi *et al.*, 2024) (bottom), which is an independent contig in the draft genome project, is also displayed. **C**) Comparative sequence coverage analysis for the two sequencing projects. Reads from the current study (see A) and from the study of Kawaichi *et. al.* (downloaded from the SRA, Bioproject PRJDB9712) were mapped to the genome sequence of *M. maripaludis* Mic1c10 determined in the current study (1 771 462 bp). For the explanation of the genome markup (in the center), see panel A. For the draft genome, average read coverage is 1154-fold, but is only 677-fold across the two 31 kb direct repeats (because reads mapped only once with random assignment to the two copies of the repeat). A drastic coverage drop is observed across the MIC island (down to 7.7-fold). **D**) Illumina reads and read pairs traversing the 221 bp near-identical duplication enclosing the MIC island (red arrows) were analyzed for connectivity of the adjacent genomic regions. In the left part (Mic1c10 configuration, MIC island present), the connectivity from the upstream chromosomal region into the 5’ end of the MIC island and from the 3’ end of the MIC island into the downstream chromosomal region is illustrated. On the right (Mic1c10mut1 configuration, MIC island absent), the direct connection of the upstream to the downstream chromosomal region is illustrated. For reads from the current study, connectivity could be established by read pair analysis. The individual reads (150 bp long) were too short to traverse the 221 bp duplication. For the draft genome, Illumina reads of 300 bp in paired mode were used, so that even some individual reads allowed to unravel the linkage. The number of clones which were encountered for each of the junctions is indicated adjacent to the sketched Illumina reads. ***For unknown reasons, Contig 3 of the draft genome does not take into account the four reads traversing the right MIC island/genome boundary.

*ycaO*_3‘-end GTGTGGATAGAGATAGAATTTCACTCTGGATTAAAGATAGGATTCGAAGAAACCTT--

MMic1c10_05855-60 **GTG**TTGATAGAGATAGAATTTCACTCTGGATTAAAGATCGGATTCGAAGAAACCTT--

QMD61_05320 --GCAGACTCAGGGAAAACAACAAC---AATTGAAAATATTCTTGAGAAAAAAGAAGA

* ** ** * ** ** *** ** ** ** * ***

*ycaO*_3‘-end GAAAGTAACTTAAATTTAATT**TAA**------------------------------------

MMic1c10_05855-60 GAAAGTAACAAAAAT**TGA**GTGCAAGGGTACAACTGTTTCCCTCGATTATGGTAATACCAT

QMD61_05320 GAAAGTAACAAAAATTGAATGTAAAGGAACAACAGTTGCACTTGATTACGGAAATGCAAT

********* ***** * * **

*ycaO*_3‘-end ------------------------------------------------------------

MMic1c10_05855-60 AATTAATGGCGAAAAAATCCATATTTTCGGCTCTCCAGGACAGGAAAGATTCAAATTCAT

QMD61_05320 GATTAACGGTGAAAAATTCCATATATTCGCCACTCCAGGACAGGAAAGATTTCAATTCAT

*ycaO*_3‘-end ------------------------------------------------------------

MMic1c10_05855-60 GCGTGAAATCCTTTCAAACGGGTTAAATGGTGCTATTGTAGTCATTGACAATTCAAGAGG

QMD61_05320 GCGTGAAATACTTTCAAACGGGTTAGATGGTGCAATTGTGGTTATAGATAACTCTAGAGG

*ycaO*_3‘-end ------------------------------------------------------------

MMic1c10_05855-60 AGTTACAGATACTGATATCCAAATAATGGATAATTTAAACACCAATAACATTCCTTATGT

QMD61_05320 AATTACAGATACTGATAAACAAATAATGGATAATTTAAACTCTAACAATATCCCTTATGT

*ycaO*_3‘-end ------------------------------------------------------------

MMic1c10_05855-60 AGTTTTTGCAAATAAACAGGATATATCTCCAGGAAATCCTGAATCTGAGTACATAGATTC

QMD61_05320 AATATTCTGCAATAAACAGGACATAGTGCCTGGAAAAATAGAATCACCGCACATTAAAGA

*ycaO*_3‘-end ------------------------------------------------------------

MMic1c10_05855-60 TCATATTCAAATAATCCCAACCATCGCAAAAGAAGGAGAAGGAATTCAGGAAGGATTAGA

QMD61_05320 AGATATCCCAATAATACCCACTACAGCGAAGTTTGGGGAAGGAATTCATGAGGGATTAGA

*ycaO*_3‘-end ----------------------------

MMic1c10_05855-60 AATTCTTCTAGAATTAGTAGAAAAC**TAA**

QMD61_05320 AACTCTTTTAGAGTTAATGGAAACT**TAA**

**Supplementary Figure S4:** **Alignment of sequences related to the HGT of the MIC island at the 5’ end.** Sequence alignment depicting the insertion of the 5’-end of MIC neighborhood 1 from *Methanobacteria* into *M. maripaludis*, in relation to Figure 2C. The portion of the *ycaO* gene corresponding to the first direct repeat flanking the MIC island in Mic1c10 is shown in red text. An internal nine-nucleotide motif occurring in both the *ycaO* gene (gene A in Fig. 2) and gene QMD61_05320 (gene B in Fig. 2) from isolate Yes71-06 (GenBank accession JASEIM010000008) is underlined. Putative cut sites flanking this motif are indicated by lightning bolts. Annealing of the 5’-end of the cut QMD61_05320 gene into the *ycaO* gene affects both genes (MMic1c10_05855 and MMic1c10 05860). The *ycaO* gene in Mic1c10 (MMic1c10_05855) is mutated at its extreme C-terminus, the original tetrapeptide LNLI being replaced by the dipeptide KN. The gene MMic1c10_05860 is either inactivated (due to truncation at the N-terminus) or receives an altered N-terminal sequence in case the GTG codon (green) functions as a start codon (Tsurumaru *et al.*, 2018). In this case, the two genes have an atypically long gene overlap. Stop codons are in bold and highlighted red. Note that the corresponding triplets TGA and TAA in QMD61_05320 are out of frame (in frame codons are indicated by yellow and grey shading). We note that Buetzberg also has a sequence similar to the homologous motif region with three mismatches: AAAATTACCAAAATTGA (not shown in alignment). The first 45 bases of QMD61_05320 are not included in the alignment. Alignment was made with CLUSTALW.

QMD61_05385 AAAGAGATACCCATATTAAAAACTGTTGTTCCAATAAAGTTAAATGGACGTGAGTGCATT

MCBB_1258 AATAAAATACCCATATTAAAAACTGTGATTCCAATAAAATTAGTAGGACGTGAATGCCTC

MMic1c10_05925-30 AAAGAGATACCCATATTAAAAACTGTTGTTCCAATAAAGTTAAATGGACGTGAGTGCATT

*ycaO*_3’-end ------------------------------------------------------------

QMD61_05385 CTTGAAAGTTTTATTGA-------------------------------------------

MCBB_1258 CTTGAAAGTTTTATTGATATGACTGAACGTAAAAAAATGGAAAATGCCTTAAAACATAGC

MMic1c10_05925-30 CTTGAAAGTTTTATTGA-------------------------------------------

*ycaO*_3’-end ------------------------------------------------------------

QMD61_05385 ------------------------------------------------------------

MCBB_1258 GAAGAAAGTTTCAGGGCTTTATCTGATGATTCAATTGACCTTATCATGCGCCATGATAGG

MMic1c10_05925-30 ------------------------------------------------------------

*ycaO*_3’-end ------------------------------------------------------------

QMD61_05385 ------------------------------------------------------------

MCBB_1258 GAGCATAGACATCTTTATGTGAACCCCATTGTTGAAAAGTTTATAGGTATTCTGCCTGAG

MMic1c10_05925-30 ------------------------------------------------------------

*ycaO*_3’-end ------------------------------------------------------------

QMD61_05385 ------------------------------------------------------------

MCBB_1258 GACTTTATAGGTAAAACACTTAAGGAAATGGGGTTTCCAAAGGATTTAGTGAAATTGTGG

MMic1c10_05925-30 ------------------------------------------------------------

*ycaO*_3’-end ------------------------------------------------------------

QMD61_05385 ------------------------------------------------------------

MCBB_1258 GAGAAAGCTATTGACAAAGTATTCAAAACAAAAAAAAATAACCACATTGAATTCGAACTC

MMic1c10_05925-30 ------------------------------------------------------------

*ycaO*_3’-end ------------------------------------------------------------

QMD61_05385 ------------------------------------------------------------

MCBB_1258 CCGAAGGGTATATGGATAGATGCATTGTTAGTGCCAGAATTTGATGAGAAAGGTGATGTA

MMic1c10_05925-30 ------------------------------------------------------------

*ycaO*_3’-end ------------------------------------------------------------

QMD61_05385 --------------------------CATCACTGAGCGTAAAAAGATGGAAATGGCATTG

MCBB_1258 AAAATTGTTTTAACTTCAGCACGTGACATCACTGAACGTAAAAAGATGGAAACCGCCTTG

MMic1c10_05925-30 --------------------------CATCACTGAGCGTAAAAAGATGGAAATGGCATTG

*ycaO*_3’-end ------------------------------------------------------------

QMD61_05385 TCATGGGAAATGGCAATTAATAATGCTTTGGCTAAATTATCAAAAAAACTCCTCTCTCAA

MCBB_1258 TCATGGGAAGTTTCAATTAACAATGCTTTGGCTAAATTATCAAGAAATCTCCTGTCTCAA

MMic1c10_05925-30 TCATGGGAAATGGCAATTAATAATGCTTTGGCTAAATTATCAAAAAAACTCCTCTCTCAA

*ycaO*_3’-end ------------------------------------------------------------

QMD61_05385 GCCTCAATTGAGGATATCACATATCTTGTTTTAAAATATGCCAAGGATCTAACCCAAAGC

MCBB_1258 GCCTCAATTGATGATATTTCATATCTTGTTTTAGAACATGCCAAAGATCTAACCTGCAGC

MMic1c10_05925-30 GCCTCAATTGAGGATATCACATATCTTGTTTTAAAAT**ATG**CCAAA----**TAA**TGCAAAAT

*ycaO*_3’-end -----------------------------------ATATGCCAAA----TAATGCAAAAT

* ******* *** *

QMD61_05385 AAAGATGGTTTTGTTGGATATATTGATCCAGAAACAGGTTATTTAATAGTTCCTAACCCA

MCBB_1258 CAATACGGTTTTGTTGGATATATAAATCCTAAAACAGGTTATCTAATGGTTCCTACCCTC

MMic1c10_05925-30 TAA----ATCTTAAAAAAGACAT------TGAAACAG----TTAAAGAAATATTAAAACA

*ycaO*_3’-end TAA----ATCTTAAAAAAGACAT------TGAAACAG----TTAAAGAAATATTAAAACA

** * ** * * ** ****** * ** * **

QMD61_05385 ACACGAAATTTCAGGGAAAAACGTAAGATTAAAGACAAGAACATAATTTTTAAGACATTT

MCBB_1258 AAACGAGATTTCCGGAAAGAATTAAAGAAAAAAAATAAGAAAACAGTTTTTAAGAAATTT

MMic1c10_05925-30 GAACGG---TTTTGATAAAATCATAACTGTTAAATTAAATAAA-ACTGATATAGATGTTT

*ycaO*_3’-end GAACGG---TTTTGATAAAATCATAACTGTTAAATTAAATAAA-ACTGATATAGATGTTT

*** ** * ** * ** ** ** * * * * * *** ***

QMD61_05385 CGAGGATTGTGGGGTTGGGTGTTGAATAATAAAGAGTCAATTCTCACAAATGATCCAGCA

MCBB_1258 AAGGGATTGTGGGGTTGGGTGTTGAATAATAAAGAGTCAATCTTCACCAATGATCCTGCT

MMic1c10_05925-30 CAAGAGTAATTATTCCTAAAATGGAAATGTACAGTGTTGATAGAGATAGA--ATTTCACT

*ycaO*_3’-end CAAGAGTAATTATTCCTAAAATGGAAATGTACAGTGTTGATAGAGATAGA--ATTTCACT

* * * * *** ** ** ** ** * * ** *

QMD61_05385 TGGGATCCAAGATCAACTGGAACTCCTGAAAGTCAT-ATTGCAATCAATAGTTTTGTATC

MCBB_1258 TGGGATCCAAGATCAACTGGAACTCCTGAGGACCAT-ATCGCAATTAACAATTTTGTATC

MMic1c10_05925-30 CTGGATTAAAGAT-----AGGATTCGAAGAAACCTTGAAAGTAACTTA-AATTTAATT**TA**

*ycaO*_3’-end CTGGATTAAAGATC-----GGATTCGAAGAAACCTTGAAAGTAACTTA-AATTTAATT**TA**

**** ***** * * ** * * * * ** * * *** * *

QMD61_05385 TGCTCCCGCAATGATAGAGGATAAATTAGTAGGGCAAGTTGCTTTGGCTAACTCAGATCA

MCBB_1258 CGCCCCTGCGATGATAGAAGACAAATTAGTAGGGCAAATTGCTTTAGCTAACGCAGACCA

MMic1c10_05925-30 **A**-----------------------------------------------------------

*ycaO*_3’-end **A**-----------------------------------------------------------

QMD61_05385 TGATTATACTAAAGAAGATCTAAAACTGATAGAACGTTTAGCAGATATCTATGCTATTGC

MCBB_1258 TGATTATACTAAAAAAGATTTGGATTTAGTAGAACGTTTAGCTGATCTCTATGCCATTGC

MMic1c10_05925-30 ------------------------------------------------------------

*ycaO*_3’-end ------------------------------------------------------------

QMD61_05385 TATTAACCGTCAGTTATTAGAGGAAAAAATTCATAAGAGTGAAGAAAAACACAGGAAAAT

MCBB_1258 TATTAATCGTGAATTATTAGAAGAAAAAATTCGTGGAAGTGAAGAAAAACATAGAAAAAT

MMic1c10_05925-30 ------------------------------------------------------------

*ycaO*_3’-end ------------------------------------------------------------

QMD61_05385 TGTTGAAAAATTTTTAGAAACTGTATCCGAGAATAAG**TGA**

MCBB_1258 AGTTGAAAAATTTTTAGAAACTGTGTCAGAGGAT**TAA**---

MMic1c10_05925-30 ----------------------------------------

*ycaO*_3’-end ----------------------------------------

**Supplementary Figure S5: Alignment of sequences related to the HGT of the MIC island at the 3’ end.** Sequence alignment depicting the insertion of the 3’-end of MIC neighborhood 1 from *Methanobacteria* into *M. maripaludis*, in relation to Figure 2C. The portion of the *ycaO* gene (gene A in Fig. 2) corresponding to the second direct repeat flanking the MIC island in Mic1c10 is shown in red text. An internal nine-nucleotide motif occurring in both the *ycaO* gene and gene QMD61_05385 (gene C in Fig. 2) is underlined. Putative cut sites flanking this motif are indicated by lightning bolts. Annealing of the 3’-end of the cut QMD61_05385 gene into the *ycaO* gene would result in an overlap of genes (MMic1c10_05925 and MMic1c10_05930). Thus, MMic1c10_05925 stemming from the QMD61_05385 gene from *Methanobacteria* has a 3’ truncation due to the introduction of a stop codon TAA (black highlight). This truncation occurs at a domain boundary and would remove the C-terminal GAF domain which occurs in QMD61_05385 (purple text). The TAA triplet in *ycaO* 3’-end is out of frame (in frame codons are indicated by yellow and grey shading). A short protein corresponding to the 3’-end of the *ycaO* gene (gene A’ in Fig. 2) is encoded by an in-frame ATG (in green) start codon (MMic1c10_05930). Other stop codons are in bold and highlighted red. We note that Buetzberg (MCBB_1258) also has a sequence similar to but not completely conserved with the homologous motif region in addition to a DNA stretch that encodes an additional internal PAS domain (cyan text). 3’-ends of indicated genes (only locus tag numbers are listed for each stain/isolate) were used for alignment. Alignment was made with CLUSTALW.

**
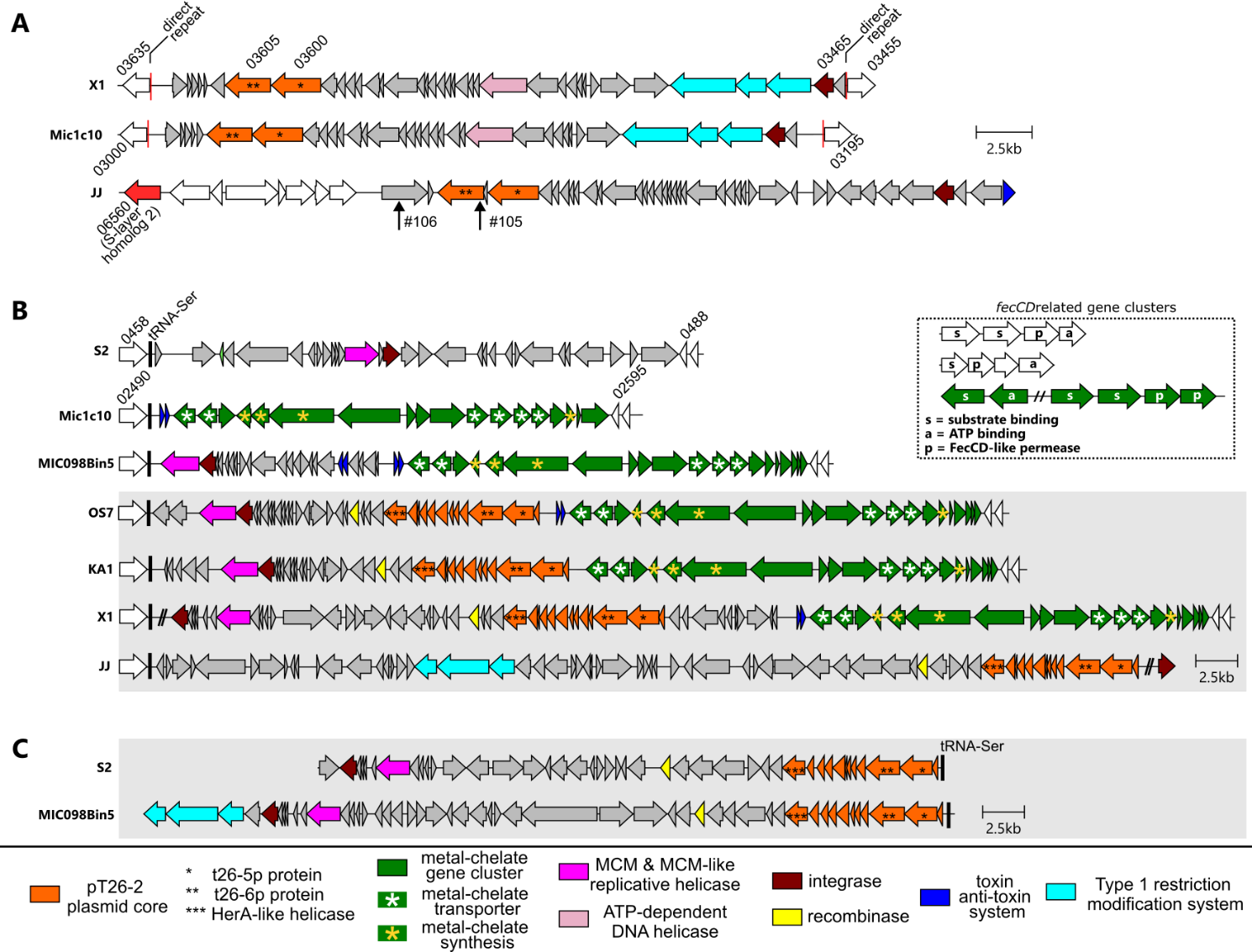
**

**Supplementary Figure S6: Gene cluster comparison of regions related to pT26-2 plasmids and/or metal-chelate transport and synthesis in *M. maripaludis* strains. A)** The divSEG KA32/OS29 in Mic1c10 (second row, see Suppl. Table S9/S10) is very similar to a region which occurs in X1 (see also Suppl. Table S4). These regions encode genes distantly related to t26-5p (*) and t26-6p (**) proteins, which also belong to the core genes within the pT26-2 family of plasmids (orange). As these gene clusters lack five out of seven of the core genes, they are distinguished as pT26-2-like. The entire pT26-2-like integrative element in Mic1c10 and X1 is enclosed by a 20 bp direct repeat (red line). A further pT26-2-like mobile genetic element is also found in strain JJ and is located at the position of glycan biosynthesis cluster 1 (part of the 40 kb intervening sequence indicated in Fig. 3A, here shown in the reverse direction). Arrows (#106/#105) refer to two genes that closely match CRISPR spacers in Mic1c10 and OS7 (Fig. S7C, Suppl. Text S6, spacer numbers refer to the Mic1c10 CRISPR array). **B**) Strains containing a pT26-2 plasmid are marked by the grey box. These plasmids are integrated at or near a tRNA-Ser (black bars) downstream of MMic1c10_02490 orthologs. The pT26-2 plasmids are characterized by pT26-2 core genes (orange), a recombinase (yellow), an integrase (brown) and a MCM helicase (purple). MIC098Bin5, S2, and Mic1c10 do not contain a pT26-2 plasmid in this genetic region (but the MCM helicase and integrase exists in MIC098Bin5). Directly downstream of the pT26-2 plasmid in strains OS7, KA1, and X1 is a gene cluster for putative metal-chelate synthesis and transport (green). Mic1c0 and MIC098Bin5 also have this gene cluster along with a toxin anti-toxin system (blue) but non-corrosive strains S2 and JJ do not. The inset compares the six genes of the metal-chelate transporter to two other sets of genes encoding ABC-type II FecCD-like metal-chelate transporter systems in Mic1c10 composed of periplasmic substrate binding proteins, ATP-binding proteins, and FecCD-like permeases. The first set ranges from MMic1c10_06525 to MMic1c10_06540 and the second from MMic1c10_02690 to MMic1c10_02675. **C**) a pT26-2 plasmid is integrated at a different tRNA-Ser in strains S2 and MIC098Bin5. Other genes of interest are labeled in color and/or with locus tag numbers for orientation (see also (Soler *et al.*, 2010, Badel *et al.*, 2019)).

**A**

**
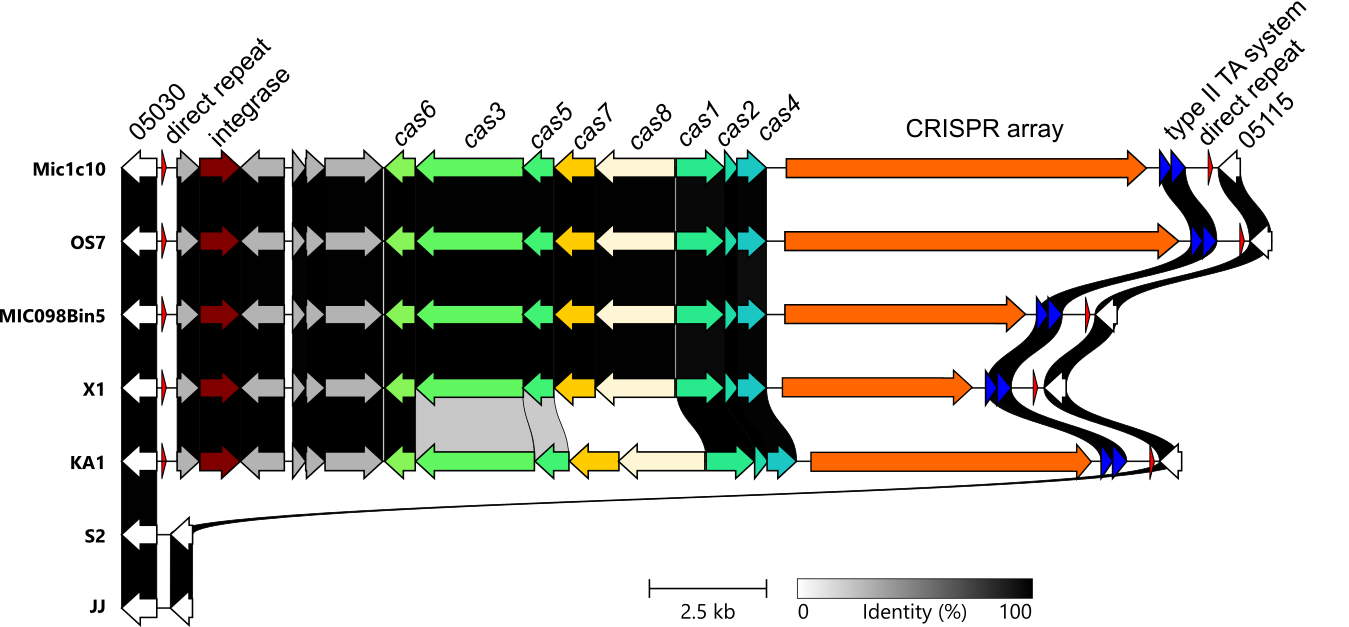
**

**B**


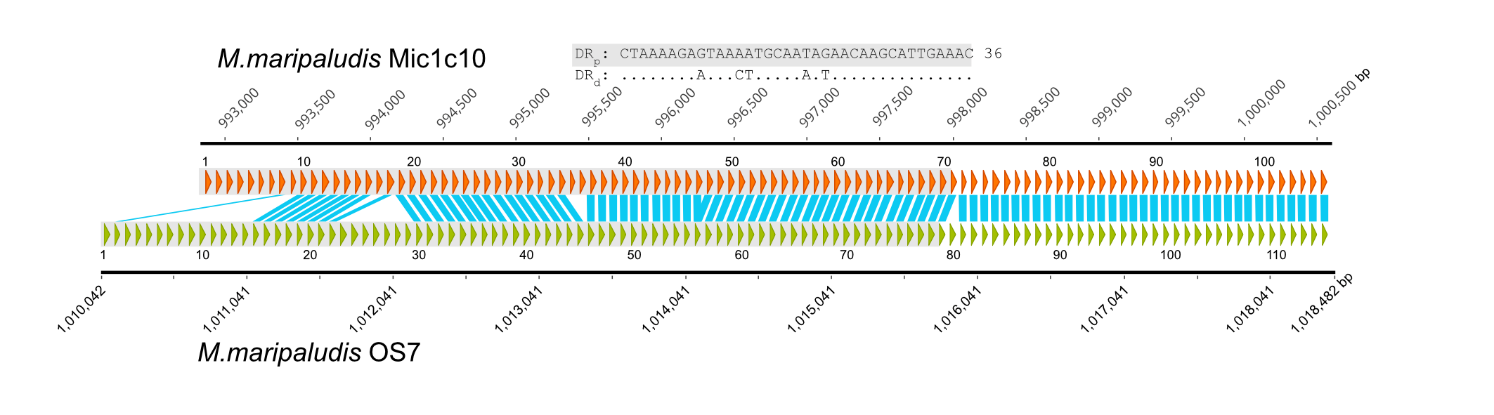


**C**

OS7_#114 AAAGTTACTGAATTaATCTCAGTATGGCCGATAAC

Mic1c10_#105 AAAGTTACTGAATTaATCTCAGTATGGCCGATAAC

MMJJ_06660 AAAGTTACTGAATTtATCTCAGTATGGCCGATAAC

************** ********************

**D**

OS7_#115 TTAAAAATCAACCTGAAACaCCTTCTTTTAAACCT

Mic1c10_#106 TTAAAAATCAACCTGAAACaCCTTCTTTTAAACCT

MMJJ_06640 TTAAAAATCAACCTGAAACcCCTTCTTTTAAACCT

******************* ***************

**Supplementary Figure S7: CRISPR-Cas systems in *M. maripaludis*** **strains**. **A**) gene cluster alignment of CRISPR-Cas systems flanked by conserved genes (MMic1c10_05030-05115). The presence of direct repeats (red) suggests the CRISPR-Cas system to be a mobile element containing also an integrase (brown) and a type II toxin anti-toxin (TA) system (blue). Cas proteins from strains Mic1c10, KA1, and OS7 are listed in Suppl. Table S5. **B**) Comparison of the CRISPR spacer arrays of strains Mic1c10 and OS7. Spacers are indicated as colored triangles (orange, Mic1c10; green, OS7). The spacers are numbered from the left, with every 10th spacer labeled. Spacers with identical (or near identical) sequences in the two strains are indicated by blue connecting strips. Shading behind the spacers and the spacer sequence (at the top of panel B) indicates the type of direct repeat (DR) used, proximal (DR_p_, shaded grey) or distal (DR_d_, no shading). Above and below the spacer arrays are the genomic coordinates of each strain. **C-D**) DNA sequence alignment showing the similarity of CRISPR spacers from OS7 and Mic1c10 with genes encoding the t26-p-like protein (C, MMJJ_06660) and MMJJ_06640 (D) (refer to arrows in Fig. S6A). Refer to Supplemental Text S6 for further details.

**
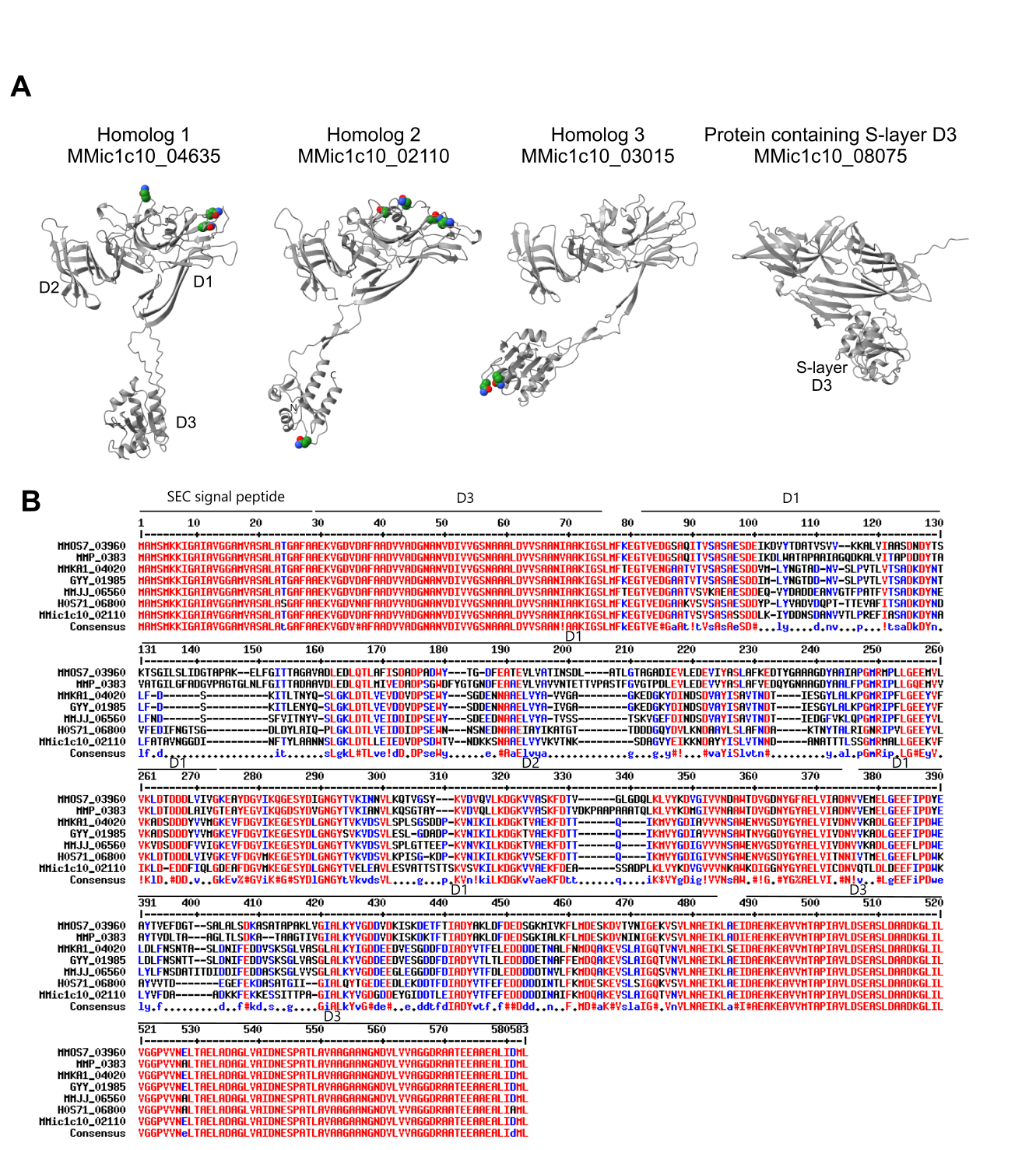
**

**Supplementary Figure S8: Structure predictions for Mic1c10 S-layer proteins and alignment of S-layer protein homolog 2 sequences from relevant strains.** In reference to Figure 4, **A**) AF models of Mic1c10 S-layer proteins and **B**) sequence alignment of S-layer protein homolog 2. For conserved and variable regions see Suppl. Table S6. Homolog 2 structural model is also shown in Fig. 4B. The three domains common to all three homologs are labeled in homolog 1 (D1, domain 1; D2, domain 2; D3, domain 3). Domains are also indicated above the sequence alignment. *M. maripaludis* strains also encode a fourth S-layer-like protein (MMic1c10_08075, right in panel A) that contains D3 and is used as an outgroup in Fig. 4A. It is also found in the other seven strains, and none are predicted to be glycosylated. The proteins listed in (B) originate from Mic1c10 (MMic1c10), KA1 (MMKAI), OS7 (MMOS7), MIC098Bin5 (H0S71), X1 (GYY), JJ (MMJJ), and S2 (MMP). Note that all homolog 2 proteins are highly divergent (in contrast to homologs 1 and 3 (Suppl. Fig. S9-10). Subregions of the proteins from strains Mic1c10, KA1, and OS7 are listed in Suppl. Table S7. Alignment was made with Multalin.


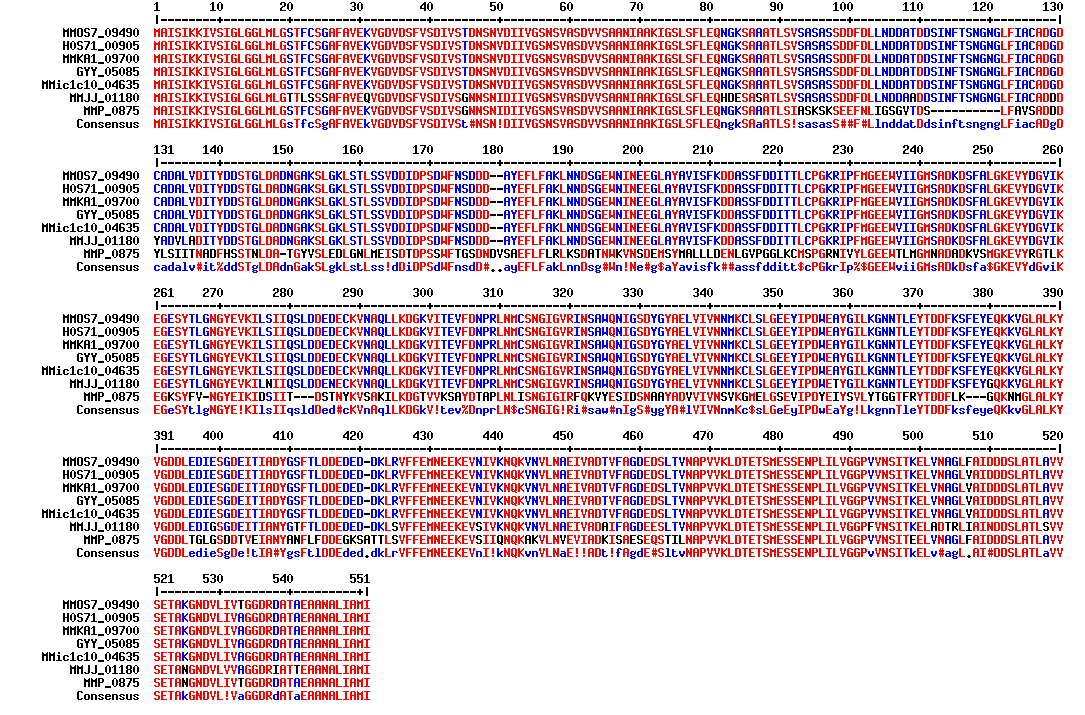


**Supplementary Figure S9: Alignment of S-layer protein homolog 1 sequences from relevant strains.** In reference to Figure 4, sequence alignment of S-layer protein homolog 1. Note that all proteins show high conservation except for MMP_0875 from strain S2, where divergent D1 and D2 result in the loss of three conserved N-X-(S/T) sequons (refer to Suppl. Table S7). For correlation between locus tags and strains see Suppl. Fig. S8. Alignment was made with Multalin.


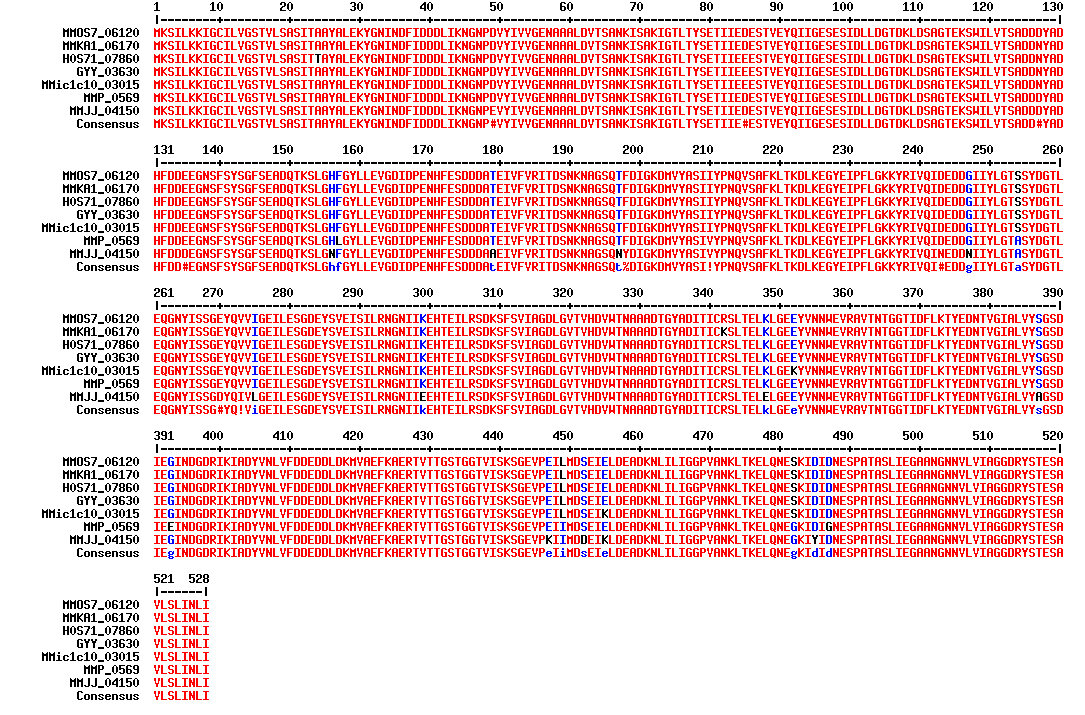


**Supplementary Figure S10**: **Alignment of S-layer protein homolog 3 sequences from relevant strains.** In reference to Figure 4, sequence alignment of S-layer protein homolog 3. Note that all proteins are highly conserved. For correlation between locus tags and strains see Suppl. Fig. S8. Alignment was made with Multalin.


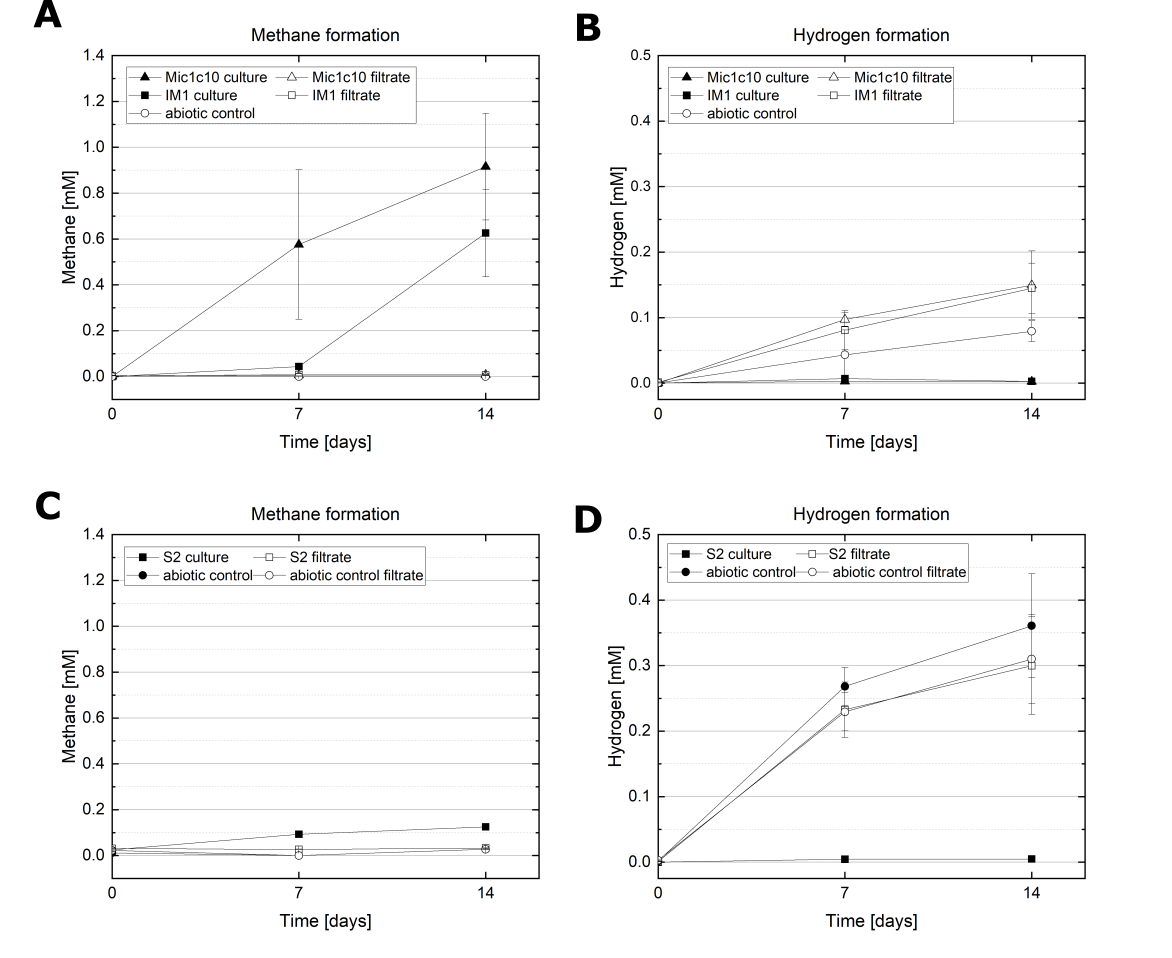


**Supplementary Figure S11: Methane formation and hydrogen formation during spent filtrate corrosion experiments in the presence of iron coupons in Artificial Sea Water.** **A**) The graph shows the methane formation [mM] in the culture and spent filtrate headspaces of *M. maripaludis* Mic1c10 and *Methanobacterium* IM1, respectively, at day 0 and after an incubation time of 7 and 14 days. **B**) The hydrogen formation [mM] in the culture and spent filtrate headspaces of *M. maripaludis* Mic1c10 and *Methanobacterium* IM1, respectively, at day 0 and after 7 and 14 days of incubation is shown. **C-D**) Show the same representative data for the non-corrosive *M. maripaludis* strain S2. The error bars show the standard deviation of at least three replicates.


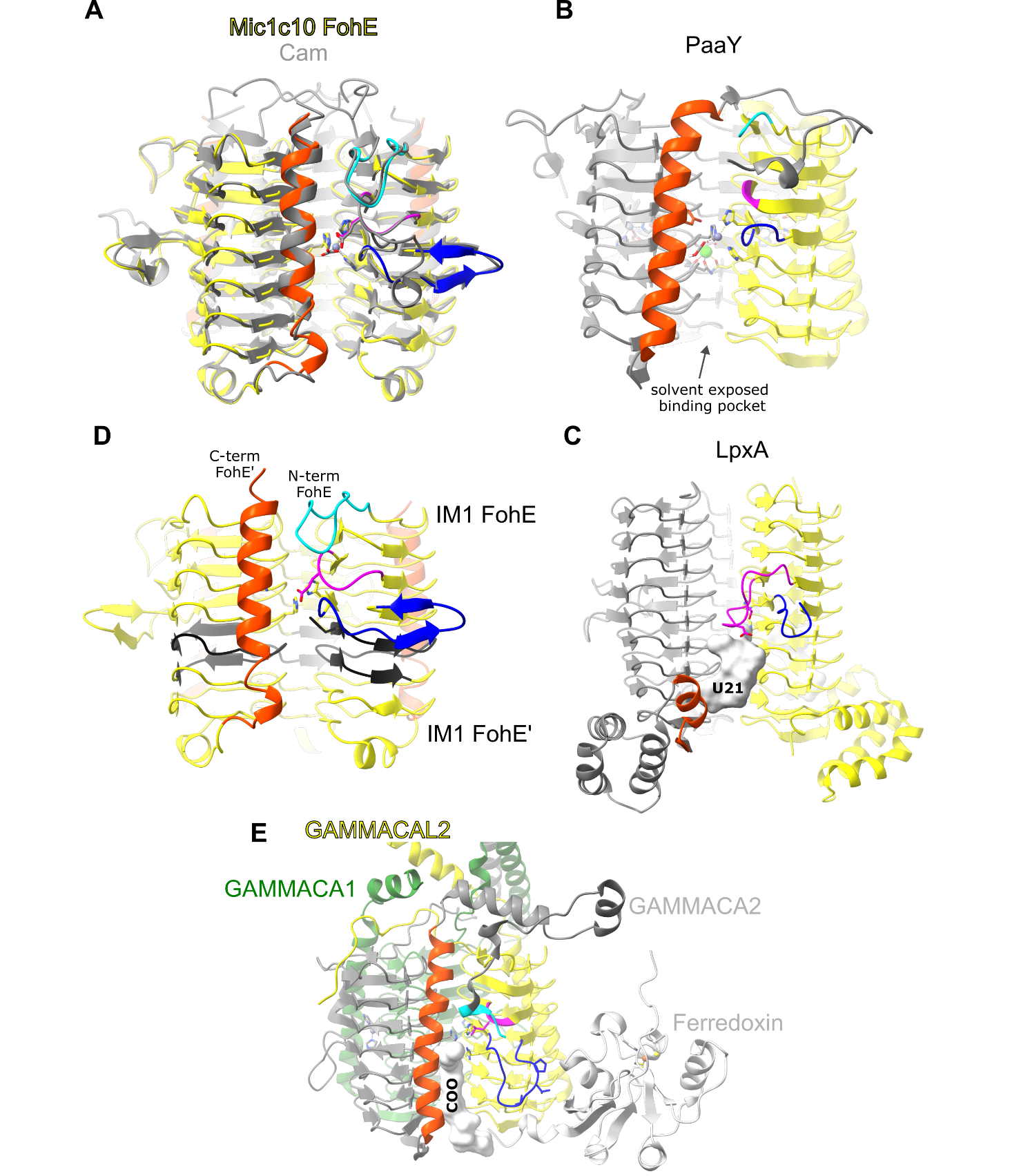


**Supplementary Figure S12: Structural comparison of FohE homologs to the γ-carbonic anhydrase Cam and other β-helix proteins. A)** Overlay of AF model of Mic1c10 FohE with Cam (grey, PDB 1QRL). Coloring of FohE is the same as in Fig. 6C. For sequence alignment see Suppl. Fig. S13A . Signal peptides have been removed. **B)** Crystal structure of PaaY (PDB 8GPP) with bound bicarbonate in binding pocket 1 and binding pocket 2 solvent exposed. **C)** Crystal structure of LpxA with UDP-3-O-(R-3-hydroxydecanoyl)-GlcNAc (U21) bound in binding pocket 2 (PDB 5DG3). **D)** Due to a point mutation (see Suppl. Fig. 13D), FohE in IM1 is truncated and only contains binding pocket 1 lacking the complete metal binding site. Depending on multiple possible translational start sites, FohE’ may or may not be lacking 25 residues (black ribbon) found in Mic1c10 FohE (see Suppl. Fig. 13B-C). AF model of Mic1c10 was used to map residues for IM1 FohE/E’. **E)** Crystal structure of a heterotrimeric complex of β-helix proteins (GAMMACA1, GAMMACA2, and GAMMACAL2) found in the crystal structure of the respiratory I + III supercomplex from *Arabidopsis thaliana* (PDB 8BQ5). Crotonyl coenzyme A (COO) is bound in binding pocket 2. Loop2 (blue) mediates interaction with [2Fe-2S] ferredoxin. As in Fig. 6, N-terminal loops are cyan, Loop1 is magenta, Loop2 is blue, and C-terminal α-helices covering the two binding pockets are orange/red.


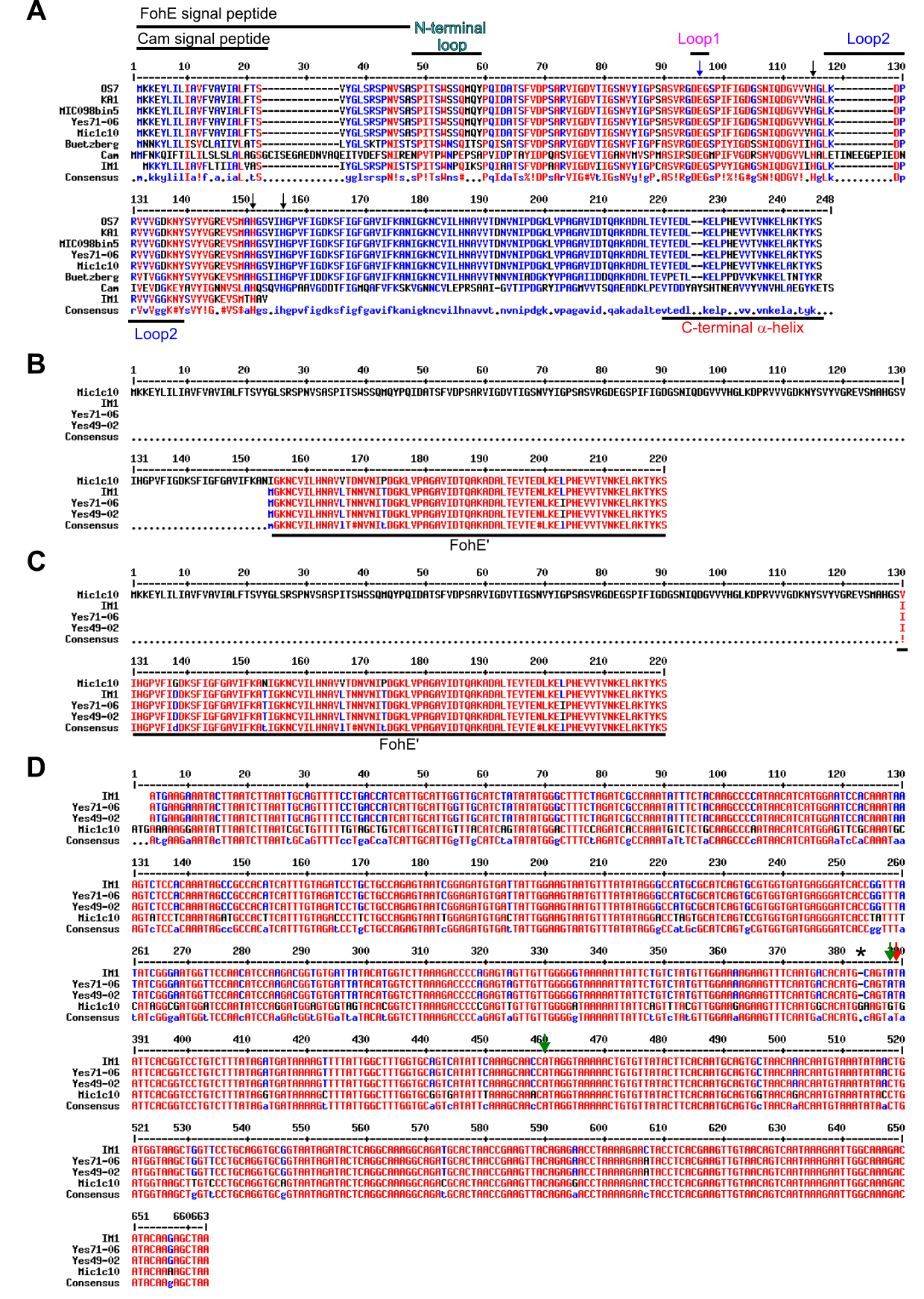


**Supplementary Figure S13:** **Alignments of FohE/E’ from relevant strains.** **A**) protein sequence alignment of FohE across the indicated strains in addition to Cam from *Methanosarcina thermophila* (UniProt: P40881, CAH_METTT). Important elements are indicated (also refer to Fig. 6 and Suppl. Fig. S12) and the three histidines involved in the coordination of Zn^2+^ are marked by black arrows. A conserved glutamate near active site is marked with a blue arrow. **B-C**) protein sequence alignments of potential FohE’ proteins against FohE from Mic1c10. FohE’ is annotated in GenBank with an atypical ATA start codon resulting in a polypeptide of 67 residues (B) that would lack two turns of the β-helix as depicted in Suppl. Fig. S12D. Usage of an earlier non-annotated in-frame ATA start codon would result in FohE’ with 91 residues, which could build a full length FohE/E’ heterodimer analogous to full length FohE in Mic1c10 (C, refer to Suppl. Text S11). **D**) nucleotide sequence alignment of *fohE* from Mic1c10 against the coding region for *fohE* and *fohE’* in the indicated strains where FohE is truncated. Large asterisk (*) denotes a point mutation found in strains IM1, Yes71-06, and Yes49-03. Red arrow marks the premature stop codon for *fohE* in IM1, Yes71-06, and Yes49-03. Green arrows denote two non-standard ATA start codons for *fohE’*. Alignments were made with Multalin. Yes71-06b was used for alignments.


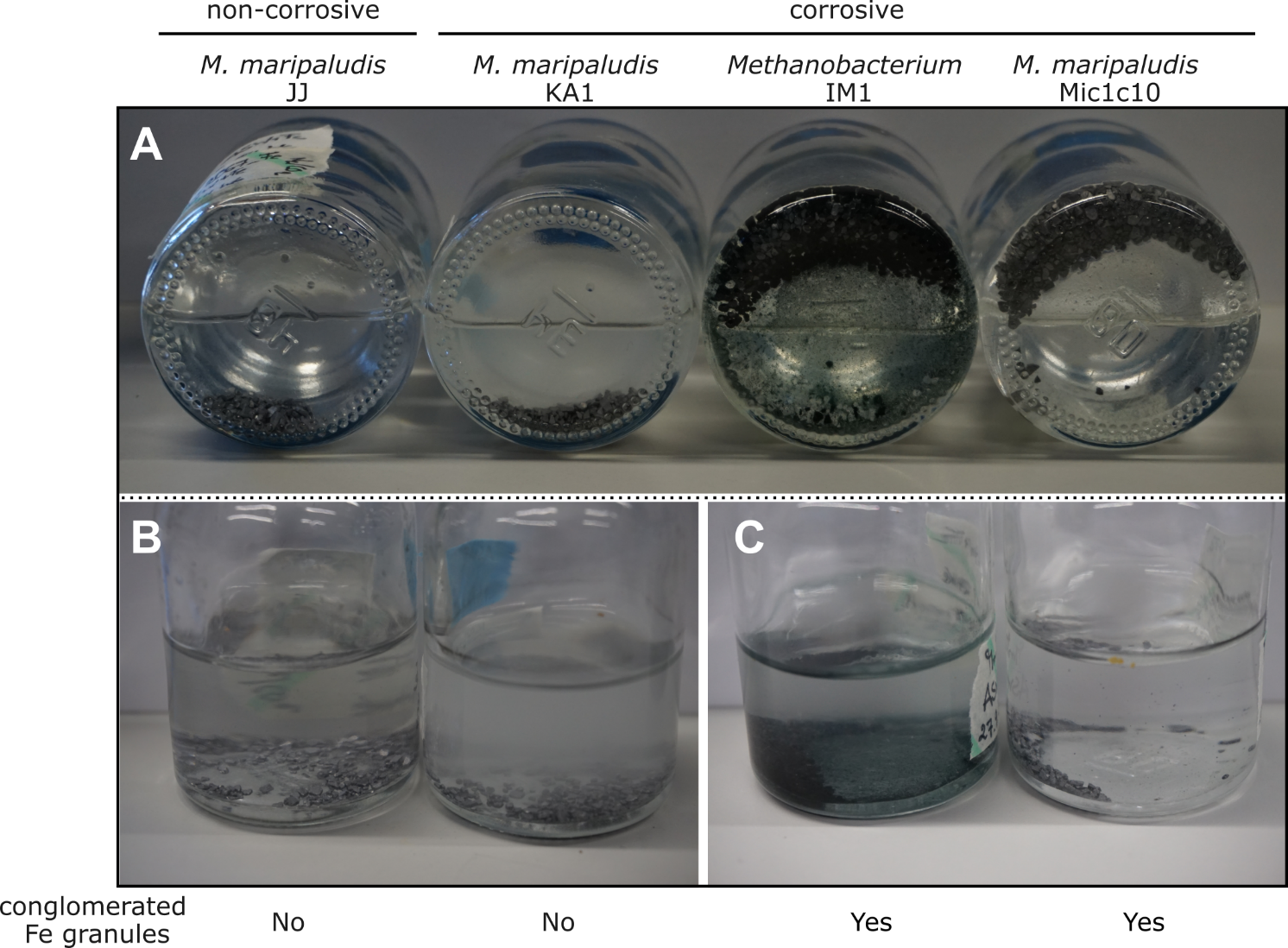


**Supplementary Figure S14: Static cultures of methanogens grown on Fe granules.** Images of cultures grown on Fe granules as the sole electron donor in 20 mL of ASW media under an N_2_/CO_2_ (80:20, v/v, 1 bar) gas atmosphere for the indicated corrosive and non-corrosive strains in 60 mL serum flasks. **A**) Flasks were gently shaken by hand and then placed on their side for capturing the image. Iron granules in cultures of JJ and KA1 did not conglomerate and settled to the bottom of the flask. On the contrary, the granules in cultures of IM1 and Mic1c10 stuck to the side of the flask, an indication of biofilm formation and/or adherence induced by corrosion products. **B-C**) Images reflect the state of the same cultures from (A) after gently shaking and then placing the flasks upright. In (B), iron granules were dispersed over the bottom of the flask. In (C), the iron granules remained conglomerated on the side of the flask. Whereas the culture of JJ showed very little signs of cell growth, the culture of KA1 had turbid media (A-B). Cultures of Mic1c10 and IM1 displayed distinct signs of corrosion as evidenced by the formation of black precipitates on the iron granules (A,C). In this regard, IM1 shows the most extensive sign of corrosion.

**2. Supplementary Texts and Methods**

**Supplementary Text S1: Genomic assembly and additional features.**

The *M. maripaludis* Mic1c10 chromosome was adjusted to that of *M. maripaludis* KA1 (GenBank: AP011526), which is one of the most closely related strains. The chromosomal ring is opened such that the genome starts with one of the rRNA operons on the reverse strand. This configuration is also used for other genomes from *M. maripaludis* (see Figure 1, Suppl. Figure 1). The convention of choosing a position close to an orc/cdc-6 type replication protein has not been adopted for strains of *Methanococcus* because the gene encoding such a protein has not been found in the genome (Hendrickson *et al.*, 2004). However, detailed bioinformatic analyses have since assigned arCOG00472 members (MMic1c10_00170; MMP0033 in *M. maripaludis* S2) as the orc/cdc6 equivalent in *Methanococcales* (Makarova & Koonin, 2013).

GC-skew and GC-content changes are indicated in rings three and four of Figure 1, and a prominent inflection of GC-skew at around 40 kb corresponds to the inferred replication origin near genes encoding MCM helicase (MMic1c10_00155) and Orc/Cdc6 (MMic1c10_00170).

**Supplementary Text S2: Comparison of the *M. maripaludis* Mic1c10 genome to those of *M. maripaludis* strains KA1 and OS7.**

The chromosome from *M. maripaludis* Mic1c10mut1 was compared separately to those of *M. maripaludis* strains KA1 (Suppl. Table S9) and OS7 (Suppl. Table S10), using the same strategy that was previously applied to strains of *Halobacterium salinarum* (Pfeiffer *et al.*, 2020). Briefly, sequence chunks of 400 kb were aligned using MAFFT (Katoh & Standley, 2013) and matching segments (matchSEGs) were identified as contiguous regions of high sequence similarity (typically 97-99% nucleotide sequence identity). Alignments are considered contiguous when they are devoid of long (200 bp) regions with reduced sequence similarity and did not have long indels or duplications. The start of the last matchSEG was used as starting point for the next chunk of 400 kb. By this approach, the complete chromosome was traversed. Genome rearrangements were encountered neither upon comparison to *M. maripaludis* KA1, nor to *M. maripaludis* OS7, which facilitated the analysis. MatchSEGs are separated, at least in one of the two compared strains, by a divergent segment (divSEG), which corresponds to an indel or to equivalently positioned but unrelated sequences in the compared strains (referred to as a replacement). In a second pass, matchSEG and divSEG candidates were finalized. A consistent handling of borderline cases was attempted for the comparison to *M. maripaludis* strains KA1 and OS7, respectively. All segments were analyzed along the chromosome by BLASTn analysis. Boundaries were shifted, matchSEGs were fused or split, and new matchSEGs were assigned as appropriate when borderline cases with respect to indels and regions with reduced sequence similarity were encountered. The final version of a matchSEG was validated by alignments with MAFFT and with BLASTn. Sequence identity values were taken from BLASTn alignments. Additional segment categories (repeatSEG, crisprSEG) allowed to resolve complex cases of sequence relationship, as described below and detailed in the legend to Suppl. Tables S9 and S10. In order to allow a three-way comparison between the three *M. maripaludis* strains Mic1c10, OS7, and KA1, the segments (as listed in Suppl. Tables S9 and S10) were integrated into a single table (Suppl. Table S11).

In the comparison of *M. maripaludis* Mic1c10mut1 to *M. maripaludis* strains KA1 and OS7, the MIC island of those strains was a divSEG, and insertion in KA1/OS7 compared to the sequence of Mic1c10 available at that time. This observation led to the conclusion that a spontaneous mutant (Mic1c10mut1) and not the wildtype (Mic1c10) had been sequenced using cells grown with H_2_ as the electron source. It triggered a second round of genome sequencing using cells grown under selection pressure (presence of iron granules), which resulted in the complete genome sequence of *M. maripaludis* Mic1c10, including its MIC island. After validation that several Illumina reads traverse the 221 bp direct repeat at both ends of the MIC island, its sequence was integrated manually into the Mic1c10mut1 chromosome sequence to result in the complete sequence of the Mic1c10 chromosome. As this MIC island is near-identical to those of *M. maripaludis* strains KA1 and OS7, this divSEG disappeared when the comparison to *M. maripaludis* Mic1c10mut1 was upgraded to that of *M. maripaludis* Mic1c10. The enclosing matchSEGs fused into a single, longer one. The extended matchSEG was confirmed by MAFFT and BLASTn alignment. The disappearance of the divSEG highlights that the MIC islands of the three strains are extremely similar so that differences are below the threshold for a divSEG assignment. The tables with matchSEGs and divSEGs was updated without repeating the complete analysis procedure. In all three strains, the left copy of the 221 bp repeat is identical. In Mic1c10, the right copy of the repeat has a single point mutation compared to the left copy. This point mutations is also found in strains KA1/OS7. These strains have, however, an additional point mutation in the right copy.

RepeatSEGs are matching segments which occur as tandem repeats in at least one of the strains, with a different number of copies in the two strains. In the comparison to strain KA1, repeatSEGs range in size from 79 bp to 31 kb. In 5 cases, there is one copy in one strain and two in the other. The other 3 repeatSEGs deviate from this (3+1; 4+1; 3+2). In the comparison to OS7, repeatSEGs range in size from 201 bp to 31 kb. The latter occurs as two contiguous 31 kb sequences in *M. maripaludis* Mic1c10 (repeatSEG OS06) but as a single interrupted sequence in *M. maripaludis* OS7 (interruption: divSEG OS07). In 3 cases, there is one copy in one strain and two in the other. One other repeatSEGs deviates from this (3+2). One segment is classified as repeatSEG even though it matches between *M. maripaludis* strains Mic1c10 and OS7 (2 copies in each strain), because *M. maripaludis* KA1 has only a single copy (KA42; OS39).

The category crisprSEG was assigned for the CRISPR array. In *M. maripaludis* strains Mic1c10 and OS7, this array is a hybrid with the 2^nd^ part sharing the CRISPR repeat with that from *M. maripaludis* KA1 (KA47; OS44), while the 1^st^ part has a variant of the CRISPR repeat (5 base differences) (KA46; OS43) (see Suppl. Fig. S7B and Suppl. Text S6).

MatchSEGs may be separated by a divergent segment (divSEG). This contains either distinct sequences at an equivalent genome position in the two strains (thus representing a replacement) or represents an indel with a sequence present in only one strain. For several indels, the strain having the sequence also contains a sequence duplication at the boundary, while the other strain lacks one copy of the duplication plus the intervening sequence. In many cases, a divSEG corresponds to independent, strain-specific sequences located at an equivalent genome position. In two cases, the divSEG sequences are highly similar (80% to 84% nucleotide sequence identity; KA51/OS48, KA53/OS50) but are categorized as divSEG as this value is well below the sequence similarity of a matchSEG (typically at least 97% nucleotide sequence identity).

In the comparison with *M. maripaludis* KA1, we assigned 37 matchSEGs, which together cover nearly 1.59 Mb, corresponding to 90% of the chromosome of *M. maripaludis* Mic1c10 and 86% of *M. maripaludis* KA1 (Suppl. Table S9). MatchSEGs range in size from 586 bp to 186 kb. We assigned 27 divSEGs, which range in size from 259 bp to 65.8 kb (for the longer sequence). The majority of these (16) have a sequence in each strain, while seven have a sequence only in *M. maripaludis* KA1 and four only in *M. maripaludis* Mic1c10. See Suppl. Table S9 and its legend for further details. In total, the chromosomes from *M. maripaludis* strains Mic1c10 and KA1 can be described with as few as 72 segments in total, highlighting their extremely close similarity and the complete absence of genome rearrangements.

In the comparison with *M. maripaludis* OS7, we assigned 34 matchSEGs, which together cover 1.56 Mb, corresponding to 89% of the chromosome of both strains (Suppl. Table S10). MatchSEGs range in size from 423 bp to 197 kb. We assigned 28 divSEGs, which range in size from 325 bp to 66.4 kb (for the longer sequence). The majority of these (15) have a sequence in each strain while eight have a sequence only in *M. maripaludis* OS7 and four only in *M. maripaludis* Mic1c10. See Suppl. Table S10 and its legend for further details. In total, the chromosomes from *M. maripaludis* strains Mic1c10 and OS7 can be described with as few as 67 segments in total, highlighting their extremely close similarity and the complete absence of genome rearrangements.

The analysis tool PHASTEST (May-2025) did not identify any prophages, yet several long (>15 kb) divSEGs have typical features of prophage regions with most genes encoded on the same strand, while being largely uncharacterized. Typically, such regions code for an integrase gene which typically is close to one end. Regions with this characteristic are divSEG_OS02 (18 kb), divSEG_KA20/OS21 (23-25 kb, plasmid pT26-2, see below and Suppl. Fig. S6 (Soler *et al.*, 2010, Badel *et al.*, 2019)￼; divSEG_ KA32/OS29 (29.2 kb, virus-related integrative element pT26-2-like, see main text and Suppl. Fig. S6). Also potentially related to this topic is divSEG_KA36/OS35 (KA1: 65.8 kb; OS7: 66.4 kb).

Strains OS7 and KA1 were reported to contain a copy of the integrative plasmid pT26-2, which is virus-related (Suppl. Fig. S6) (Soler *et al.*, 2010, Badel *et al.*, 2019). This plasmid is absent from the genome Mic1c10 (divSEG KA20/OS21). The att site (54 bp) corresponds to a direct repeat at the end of matchSEG KA19/OS20 and the start of matchSEG KA21/OS22. This plasmid extends from MMKA1_04860 (MMOS7_04790) to MMKA1_05150 (MMOS7_05080), with two additional ORFs at the beginning in OS7 (MMOS7_04770, MMOS7_04780). Plasmid pT26-2 is also present in strain X1, but is split. Part 1 is from the start (GYY_02375) to the resolvase (GYY_02460), part 2 from a second resolvase (GYY_04310) to the end (GYY_04225). The 54 bp att site sequence is upstream of part 1 and downstream of part 2. Splitting of the plasmid may be the reason why the limits have not been detectable previously (Badel *et al.*, 2019). The pT26-2 plasmids from strains KA1, OS7 and X1 contain the seven previously reported “pT26-2 core” proteins (t26-5p, t26-6p, t26-7p, t26-11p, t26-13p, t26-14p, t26-15p; two copies of t26-7p; MMKA1_05140 to MMKA1_05120, MMKA1_05080 to MMKA1_05040; MMOS7_05070 to MMOS7_05050, MMOS7_05010 to MMOS7_04970; GYY_04230, GYY_04235, GYY_04245, GYY_04270, GYY_04275, GYY_04285, GYY_04290, GYY_04295) (Suppl. Fig. S6).

One divSEG resulted in the replacement of the central region of an ortholog set of annotated S-layer proteins (homolog 2, MMmic1c10_02110, MMKA1_04020, MMOS7_03960). While the N-terminal and C-terminal regions are highly similar (>90% protein sequence identity), the central regions are remarkably divergent (<50% protein sequence identity) (Suppl. Fig. S8, Suppl. Table S6). However, the three strains encode three paralogous S-layer proteins with 32% to 45% protein sequence identity to each other. The two additional ortholog sets (homolog 3, MMmic1c10_03015, MMKA1_06170, MMOS7_06120; homolog 1, MMmic1c10_04635, MMKA1_09700, MMOS7_09490) show at least 99% protein sequence identity along their complete length (Suppl. Fig. S9/S10).

**Supplementary Text S3: Annotation of the protein-coding genes of the *M. maripaludis* Mic1c10 genome.**

Initial gene prediction was performed using RAST (Overbeek *et al.*, 2014). Prior to function annotation, the validity of gene calling was extensively checked. Proteins were compared by BLASTp against the complete proteomes from several strains of *M. maripaludis*, which had been downloaded from UniProt (see Suppl. Table S1). Typically, at least one strain has an ortholog with at least 95% protein sequence identity. Gene calling, especially start codon assignment, was considered valid when the majority of the close homologs represented full length alignments starting with Met-1 for both sequences. ORFs which failed simple validation were subjected to more detailed manual curation. Start codon assignments were manually validated if a TTG was assigned, which was quite frequent in this AT-rich genome (172 genes, 9.3%). To minimize missing gene calls, all intergenic regions (≥50 bp) were subjected to BLASTx analysis against the NCBI nr database, applying a previously reported procedure (Pfeiffer *et al.*, 2020). All stable RNAs were compared by BLASTx to the ORF set in order to identify and eliminate overlapping ORFs.

An initial function annotation of the genes was performed by RAST (Overbeek *et al.*, 2014). For subsequent manual curation, we used the annotation of *M. maripaludis* S2 as reference, as this annotation is extensively curated. This includes a genome annotation (Hendrickson *et al.*, 2004), a detailed review on the metabolism of *M. maripaludis* S2 (Goyal *et al.*, 2016), and 570 reactions from the whole genome metabolic model iMM518 for *M. maripaludis* strain S2 (Goyal *et al.*, 2014), based on KEGG (Kanehisa *et al.*, 2019). Additionally, proteins were compared by BLASTp against (a) the available *M. maripaludis* proteomes (see Suppl. Table S1), (b) the SwissProt section of UniProt (release 2024_10) and (c) the proteomes from 12 haloarchaeal genomes (version Oct-2024) which have been extensively curated by a Gold Standard Protein based procedure (Pfeiffer & Oesterhelt, 2015, Pfeiffer *et al.*, 2020). For each protein, an annotation consensus was attempted, based on these analyses.

**Supplementary Text S4: Annotation of the stable RNA genes of the *M. maripaludis* Mic1c10 genome.**

Annotation of stable RNAs (rRNAs, tRNAs, RNase P RNA, 7S RNA) was initiated by comparison with the corresponding gene annotations from strain *M. maripaludis* S2. Here, stable RNAs were extracted in parallel from the genome (GenBank accession BX950229) (Hendrickson *et al.*, 2004) and from RFAM (Kalvari *et al.*). The The RFAM website (http://rfam.xfam.org/) was accessed on 2 September 2019. The same set of RNAs was accessible from both sources. Positions, if distinct, were taken from RFAM. Assignment of the DNA strand was available only from Genbank. For tRNAs, the anticodon, the encoded amino acid, and the intron in one of the tRNAs was only available from Genbank. Also, one of the tRNAs extracted from GenBank was not represented in RFAM. RNA sequences were retrieved from the RFAM website and the reverse complement was computed if required. After having completely compiled the RNAs from *M. maripaludis* S2, those from *M. maripaludis* Mic1c10 were assigned by BLASTn analysis. *M. maripaludis* Mic1c10 has 39 tRNA genes that cover all the standard 20 amino acids, as well as a tRNA for selenocysteine (SelCys-TCA). Two tRNA genes (Met-CAT and Trp-CCA) contain an intron.

The ini-Met tRNAs were assigned by BLASTn analysis with the ini-Met tRNAs from *Haloferax volcanii* and *Haloarcula marismortui* (partial matches). For haloarchaea, the ini-Met tRNA assignment is based on two reports (Gupta & Brunak, 2002, Köhrer *et al.*, 2008). Haloarchaea have two Ile-tRNAs, one with the canonical anticodon GAT, the other with the “Met” anticodon CAT. The latter tRNA shifts decoding from ATG (Met) to ATA (Ile) due to the modification of C-34 to agmatidine (Köhrer *et al.*, 2008, Mandal *et al.*, 2010). *M. maripaludis* also contains the agmatidine modification (Mandal *et al.*, 2010). The corresponding tRNA in strains S2 and Mic1c10 was identified by BLASTn analysis with the corresponding haloarchaeal tRNA-Ile(CAT) from *Hfx. volcanii* and *Har. marismortui*.

**Supplementary Text S5: DNA methylation and tetramer analysis.**

Using the PacBio reads and the assembled genome sequence, base modifications were analyzed using the SMRT^®^ Analysis software (Basemods tool) (Chin *et al.*, 2013). The results are summarized in Suppl. Table S8. Three motifs with m6A modified bases were detected. GATC was the only palindromic motif, and only about half of these sites were methylated, with some sites being modified on both strands while other sites displayed no methylation. The CCCGA motif was modified at all sites but only on one strand, as the complementary sequence (TCGGG) does not contain an A base. The extended motif CAC-N6-RTTA and its complement TAAY-N6-GTG were modified at all sites and on both strands. Such bipartite recognition motifs are typical of type I restriction systems (Loenen *et al.*, 2014), and since only one restriction system of this type is annotated in the Mic1c10 genome, the three genes involved can tentatively be identified (MMic1c10_03470 to MMic1c10_03480). These genes are encoded on the 29.2 kb pT26-2-like integrative element in *M. maripaludis* Mic1c10 (Fig. 1 and Suppl. Fig. S6). Tetramer frequency analysis revealed the motif CTAG was strongly under-represented in the genome (odds ratio=0.56) while its inverse, GATC, was moderately under-represented (odds ratio=0.76).

**Supplementary Text S6: Comparative analysis of the CRISPR regions of *M. maripaludis* strains.**

Database searches (BLASTn) using the spacers showed no significant nucleotide similarity to known virus sequences, or to the genomes of Bacteria, but within the Archaea there was a remarkable similarity with the CRISPR spacers of *M. maripaludis* OS7. Both strains contain a hybrid CRISPR locus where the repeats of the proximal and distal segments differ by 5 bases (see Suppl. Fig. S7).

Mic1c10 contains one CRISPR array (nt 992 835 - 1 000 603) containing 106 spacer sequences, two of which (#47, #48) are identical (Suppl Fig. S7B). Unexpectedly, the 36 bp CRISPR repeats (CR) differ in sequence (5 base changes) between the proximal and distal ends of the array, with the first 71 CR being of one sequence, and the remaining 36 CR being the second sequence (Suppl. Fig. S7B). This pattern indicates that the CRISPR array is a genetic hybrid (Suppl. Fig. S7A-B). It is remarkable that the hybrid CRISPR array is shared between *M. maripaludis* strains Mic1c10 and OS7. A detailed comparison showed that 95 of the 106 *M. maripaludis* Mic1c10 spacers were closely similar or identical in sequence to CRISPR spacers of *M. maripaludis* OS7, and their synteny was maintained (Suppl. Fig. 7B).

Some of the Mic1c10 spacers showed high sequence similarity to mobile genetic elements of other *Methanococcus* strains. For example, spacer #24 matches an intergenic target on a 12 kb plasmid (pMMKA1) of *M. maripaludis* strain KA1. Spacers #87 and #88 match targets nearby the CDS (MmarC6_0077, MmarC6_0089) of *M. maripaludis* C6 that probably specify virus-related proteins as they are part of a genomically clustered, similarly oriented genes that include a CDS (close to MmarC6_0077) specifying a caudovirus tape measure protein (MmarC6_0079) and a CDS related to *Methanothermobacter* caudovirus psiM100 (MmarC6_0075). Spacers #105 and #106 also target two nearby CDS (MMJJ_06640 and MMJJ_06660), this time in *M. maripaludis* JJ (DSM 2067), and the matching CDS are part of a longer region with clear links to a pT26-2-like integrative element (see Suppl. Fig. S6). For example, MMJJ_06640 is a member of the anti-restriction (ArdC) family of proteins, which were first described as part of IncW plasmid pSa (Belogurov *et al.*, 2000). ArdC helps to establish a plasmid in the recipient after its conjugative transfer (Val-Calvo *et al.*, 2018).

Searches of CRISPR spacer databases (see methods) revealed a number of matches to the genome of *M. maripaludis* Mic1c10. Three spacers of *M. vannielii* SB closely match sites within the integrative element found between nt 678,951 - 708,173 (pT26-2 like element; see Fig. 1 at ca 0.7 Mb, Suppl. Fig. S6), and one spacer of *M. maripaludis* X1 targets a gene encoding glycine--tRNA ligase (MMic1c10_01235).

Upstream of the *M. maripaludis* Mic1c10 CRISPR array are *cas* genes encoding Cas proteins belonging to type IB (Suppl. Table S5). A few CDS further along is an integrase family protein (MMic1c10_05040) that is near a 102 nt sequence that is directly repeated on the other side of the CRISPR array (nt 1 001 974-1 002 075). The flanking repeats and recombinase indicate that the entire CRISPR/Cas region is carried on a mobile element of about 22.4 kb (nt 979 613-1 001 973). The CRISPR/Cas region of strain OS7 is similar and is also flanked by the same long direct repeat. Strains X1, KA1 and C5 each have a single CRISPR/Cas locus that is also flanked by direct repeats related to the repeat found in *M. maripaludis* Mic1c10. Also, the *cas* genes are highly similar between corrosive strains Mic1c10, OS7, and MIC098Bin5, while several *cas* genes from strain KA1 are only distantly related (Suppl. Table S5). They are similar to those from *M. vannielii* strain SB (DSM 1224). Strains C6, C7, JJ and S2 do not carry a CRISPR-Cas system and only carry the flanking repeat sequence in single copy. In the type strain (JJ, DSM 2067), the repeat sequence occurs between *cbiH* (MMJJ_00380) and a gene for an uncharacterized protein (MMJJ_00370).

**Supplementary Text S7: Taxonomic analysis of Mic1c10 and other *M. maripaludis* strains.**

The complete genome of *M. maripaludis* Mic1c10 consists of a single, circular chromosome of 1,771,462 bp and with a low GC content (33.0% G+C). For taxonomic assignment, the Mic1c10 genome was compared with eight other sequenced strains of *M. maripaludis* for which a complete genome sequence is available (Suppl. Fig. S2 and Suppl. Table S1). Mic1c10 was aligned by Mummer, which unravelled close similarity to all strains, especially to strains KA1 and OS7 (Suppl. Fig. S1). It was also subjected to whole-genome based analysis using the TYGS server (https://tygs.dsmz.de) (Meier-Kolthoff *et al.*, 2022). Their pairwise average nucleotide identity (ANIb) and *in silico* DNA-DNA hybridisation (DDH) values are given in Suppl. Table S2. As expected, all share high (>87%) ANI values, however the dDDH_d4_ values separate the strains into five, species-level groups. Strains Mic1c10, KA1, OS7, S2, and X1 all share dDDH_d4_ values >70%, confirming they belong to the same species. Unexpectedly, this set of strains shows only 63-65% dDDH_d4_ similarity to strain JJ (the type species of *M. maripaludis*), indicating they might represent a distinct species (Goris *et al.*, 2007). Strains C5, C6, and C7 each display dDDH_d4_ values ≤ 44% with all other strains and appear to represent distinct species. Low DNA-DNA hybridization values were noted before, but despite this they were considered to belong to the species *Methanococcus maripaludis* (Keswani *et al.*, 1996). The inferred phylogenetic tree is visualized in Suppl. Fig. S2. In summary, *M. maripaludis* Mic1c10 belongs to a set of closely related strains, best represented by strain S2 which had its genome sequenced in 2004 (Hendrickson *et al.*, 2004). The relation of this cluster to more distantly related strains, including the type strain of *M. maripaludis,* remains to be clarified, but is beyond the scope of the current work.

**Supplementary Text S8: Comparison of the complete *M. maripaludis* Mic1c10 genome to the draft genome.**

We compared the complete genome determined by us to the draft genome reported by Kawaichi *et. al.* (Kawaichi *et al.*, 2024). The draft genome consists of 38 contigs, of which only five are longer than 1 kb. Contig 4 is not relevant because this is Coliphage phiX174 (GenBank:J02482), thus being unrelated to *M. maripaludis*. The 33 shorter contigs have not been analyzed. It should be taken into account that each of the four relevant contigs (1, 2, 3, 5) represents the reverse strand compared to the complete genome, the latter being consistent in its orientation with those from strains KA1 and OS7. In summary, the draft genome is nearly identical to the complete genome, with some peculiar differences. Leaving aside a few point mutations and short gaps (affecting a total of 15 positions), the differences occur either at repeat regions (see below) or are due to unresolved regions in the draft genome.

The complete Mic1c10 genome contains two rRNA operons (reverse strand; the region from end of 5S rRNA to start of 16S rRNA; 1^st^ rRNA operon: pos 68-4819; 2^nd^ rRNA operon: pos 32,127-38,196). Contig 1 of the draft genome ( 1,691,275 bp) excludes nearly all of the rRNA operons. It starts a few bases within the 5S rRNA of the 1^st^ rRNA operon and continues in reverse direction, ending a few bases within the 16S rRNA of the 2^nd^ rRNA operon. There are several breakpoints (see below). Contig 5 (5005 bp) contains the rRNA operon (starting 157 bp upstream of the 16S rRNA, ending 7 bp downstream of the 5S rRNA). Contig 2 ( 27,398 bp) covers the region between the 2 rRNA operons, starting close to the end of the 5S rRNA of the 2^nd^ rRNA operon and ending 30 bp upstream of the 16S rRNA of the 1^st^ operon.

Three breakpoints are not relevant as they reflect unresolved regions in the draft genome sequence (each a stretch of 100 N residues).

One breakpoint is due to the 31 kb duplication in the complete genome. This duplication is present in the strain analysed in the current study but is absent from the strain analysed for the draft genome. This is a biological difference between the strain variants analysed in the two laboratories. The presence of the duplication in the strain from this study is confirmed by many PacBio reads that traverse the duplication boundary, confirming its biological origin. The duplication junction is also confirmed by a large number of Illumina reads from both sequencing attempts, those for Mic1c10 and for Mic1c10mut1. No such junction reads were observed in the Illumina sequences deposited by Kawaichi et al in the SRA database (DRR524101), confirming that the strains used by the two groups differ from each other. Also, the seeming coverage drop of Illumina reads over the 31 kb duplication (Suppl. Fig. S3) is consistent with only one copy of the repeat being present in the strain used for draft genome sequencing. Reads are randomly mapped to one of the two copies in the complete genome sequence.

One breakpoint is due to a mutation in the strain used for obtaining the draft genome. An internal segment is deleted from a sodium/alanine symporter (MMic1c10_08175, *agcS*, 52 codons, pos 107-158 of 453).

The last breakpoint is the MIC island, which is enclosed by a near-perfect 221 bp duplication (1 point mutation). In this region, the draft genome Contig 1 corresponds to the Mic1c10mut1 genome sequence in being devoid of a MIC island (Suppl. Fig. S3). The MIC island is Contig 3 ( 11,112 bp). Contig 3 is incomplete at the 3’ end where it lacks about 1 kb of the MIC island and does not contain the second copy of the enclosing 221 bp direct repeat. Otherwise, the sequence is identical between Contig 3 of the draft genome and the complete genome except for a single point mutation which is located in the shared upstream copy of the 221 bp duplication. As mentioned above, we encountered a coverage drop down to *ca.* 30% across the MIC island (Suppl. Figure S3A). The Illumina reads for the draft genome show a much more drastic coverage drop from >1000 to <10 (Suppl. Figure S3B).

Being surprised that Contig1 of the draft genome mimicked the deletion strain Mic1c10mut1, we attempted to unravel the basis for this assembly peculiarity. We thus analyzed the linkage across the 221 bp direct repeat which encloses the MIC island (Suppl. Fig. S3), which was feasible with the available data. In case of the draft genome, 300 bp Illumina reads were obtained so that individual sequencing reads may completely traverse the duplication. In both sequencing approaches, paired reads were analyzed which enabled a linkage across the duplication, even if the individual reads do not fully traverse it. For the complete genome, 25 and 45 reads link the termini of the MIC island to the unique sequences upstream and downstream of the MIC island, firmly establishing the position of the MIC island within the chromosome. We encountered 62 reads which traverse directly from the upstream to the downstream region. Thus, chromosomal copies lacking the MIC island (as in Mic1c10mut1) exists prominently in the population, even though the strain was grown under Fe^0^ selection. Consistent with the extreme coverage drop observed for the draft genome, we detected 164 reads which directly connect the upstream and downstream regions. Only two and four Illumina reads were encountered which connect the adjacent genome sequence to the MIC island. Obviously, Kawaichi *et. al.* (Kawaichi *et al.*, 2024) captured their strain just before it completely lost the MIC island. The four Illumina reads traversing the right boundary were obviously not considered in the assembly, leaving the MIC island on Contig 3 incomplete.

**Supplementary Text S9: Detailed analysis of pT26-2-related integrative elements and metal-chelate gene clusters in *M. maripaludis*:** Mic1c10 is distinguished from other corrosive and non-corrosive strains by several divergent sequences (divSEGs) represented as white gaps in the genome alignment (Fig. 1, Suppl. Text S2). The largest divSEG was identified as a variable element of 35 ORFs that shows some relation to the pT26-2 family of plasmids (pT26-2-like integrative element, Fig. 1) (Soler *et al.*, 2010, Badel *et al.*, 2019). This element encodes for two distant protein relatives (t26-5p and t26-6p) out of the seven “pT26-2 core” proteins (Suppl. Table S4) along with an integrase, an ATP-dependent DNA helicase, and a type 1 restriction modification system (Suppl. Fig. S6A). The latter might be responsible for ^m6^A modification of a bipartite recognition motif (Suppl. Text S5, Suppl. Tab. S8). The whole element is enclosed by a 20 bp direct repeat, with conserved *M. maripaludis* genes on both sides, indicating its integrative nature. Notably, only one closely related pT26-2-like element was further detected, which occurs in *M. maripaludis* X1.

Other strains of interest contain a canonical pT26-2 plasmid, including all seven “pT26-2 core” proteins, but at a different genomic position (Suppl. Fig. S6B-C). The function of this family of plasmids has been hypothesized to involve DNA transfer utilizing a putative HerA-like AAA+-helicase (Badel *et al.*, 2019, Beltran *et al.*, 2023). Mic1c10 is exceptional in not having a canonical pT26-2 plasmid (Suppl. Fig. S6). As a previous phylogenetic analysis has found evidence for the past or present interaction of pT26-2 plasmids within the *Methanococcales* order by identifying CRISPR spacers stemming from pT26-2 ORFs (Badel *et al.*, 2019), we searched for a CRISPR-Cas system in Mic1c10. Interestingly, Mic1c10 has a unique hybrid CRISPR array similar to OS7 (Suppl. Fig. S7A-B) and within it, two spacers that closely match to two genes within a pT26-2 plasmid-like mobile genetic element in strain JJ (Suppl. Figs. S6A,7C-D). One of these genes in JJ corresponds to a t26-6p-like protein, which demonstrates that *M. maripaludis* strains have been influenced by multiple pT26-2-like elements. Upstream of the CRISPR array are eight *cas* genes (Suppl. Table S5). The CRISPR-Cas system in Mic1c10 and other corrosive methanogens, but absent from S2 or JJ, is also a putative mobilizable region that is enclosed by direct repeats along with an integrase gene and a type II toxin anti-toxin (TA) system (Suppl. Fig. S7A). Overall, various integrative plasmids, mobile genetic elements, and CRISPR-Cas systems have been important in the genomic organization of corrosive *M. maripaludis* strains.

Directly adjacent to the pT26-2 plasmid in strain OS7 is a probable type II TA system followed by a gene cluster that is not found in the same genetic region in strains S2 and JJ (Suppl. Fig S6B). A closely related gene cluster is found in Mic1c10 and other strains. Gene annotation and BLASTp indicated that this gene cluster likely involves a system for handling a metal-chelate substrate (metal-chelate gene cluster, Fig. 1 and Suppl. Fig. S6) involving a tetrapyrrole ligand. This assignment is based on the presence of three genes encoding a metal chelatase (Al-Karadaghi *et al.*, 2006) in addition to a gene for a CobU-like tetrapyrrole-modifying enzyme and five genes encoding a split transporter system showing similarity to the FecCD transmembrane protein family. The latter family includes type II ABC importer systems for metal-tetrapyrrole chelates involving cobalamine (BtuCD/F), heme (HmuUV/T), or iron chelates such as iron(III) dicitrate (FecBCDE) (Staudenmaier *et al.*, 1989, Beek *et al.*, 2014). Two other distantly related FecCD family transport systems can be found in *M. maripaludis* (Suppl. Fig. S6B). As corrosive *M. maripaludis* strains would be exposed to iron and other metals resulting from the corrosion process, the metal-chelate gene cluster likely contributes to balancing metal homeostasis.

**Supplementary Text S10: Methods for hydrogen formation by spent cell-free medium.**

Investigation of hydrogen formation by spent cell-free medium of *M. maripaludis* Mic1c10, *Methanobacterium* IM1 and *M. maripaludis* strain S2 was performed based on the experiment performed by Deutzmann and colleagues (Deutzmann *et al.*, 2015). *Methanobacterium* IM1 and *M. maripaludis* Mic1c10 were cultivated in ASW medium (see Materials and methods in main article) with iron granules as sole electron source. *M. maripaludis* strain S2 was cultivated in modified *Methanogenium* Medium 141 as described previously (Lohner *et al.*, 2014, Deutzmann *et al.*, 2015). All cultures were grown under N_2_/CO_2_ (80:20%, v/v, 1 bar) gas atmosphere. To avoid the transfer of volatile compounds such as H_2_ and CH_4_, the cultures were flushed for 15 minutes with a N_2_/CO_2_ gas mixture (80:20%, v/v, 1 bar) before use. To prepare the spent cell-free medium, the stationary phase cultures were vortexed vigorously to remove any cells and enzymes that may have adhered to the iron granules. Then, the cultures were passed through a sterile filter (0.22 µm pore size) in an anaerobic chamber. 20 mL spent cell-free medium was directly filled into sterile and Hungate sealed serum bottles containing iron granules (1.5% (w/v), 1-2 mm, 99.98% purity). As control, 20 mL ASW with iron granules were inoculated with 2% of the respective microbes. Abiotic controls were included as well. Prior to incubation, the headspace of all serum bottles was flushed with a N_2_/CO_2_ gas mixture (80:20%, v/v, 1 bar). Then the cultures and controls were incubated at non shaking conditions at 30°C (*Methanobacterium* IM1) or 37°C (*M. maripaludis* Mic1c10 and *M. maripaludis* strain S2). The formation of hydrogen and methane was monitored via gas chromatography using the 8890 GC System equipped with a thermal conductivity detector (Agilent Technologies). Results are provided as Suppl. Fig. S11.

**Supplementary Text S11: Extended bioinformatic analysis of FohE and FohE’.**

The γ-carbonic anhydrase-like protein in the MIC gene cluster of Mic1c10 (MMic1c10_05920, assigned here as FohE), OS7 and KA1 (Fig. 2), is GlmU/DapH/DapD-related and LpxA-like. All these proteins have in common hexapeptide repeats, which result in the formation of a β-helix that assembles into a homotrimer (Fig. 6 and Suppl. Fig. S12) (Jenkins *et al.*, 1998, Prabha & Balaji, 2021). GlmU, along with LegH-like protein and Agl17 (Fig. 3), are acyltransferases involved in glycan biosynthesis and belong to the large β-helix superfamily of enzymes. The acyltransferases are responsible for the transfer of acyl groups, *e.g*., acetyl, succinyl, or long-chain fatty acids, to hydroxyl or amino sidechains of amino acids, sugars, and other small molecules (Prabha & Balaji, 2021). They are often encoded in gene clusters corresponding to specific biological function. Catalysis takes place near a variable loop (blue, Loop2, Fig. 6A), which bridges two substrate binding pockets as exemplified by GlmU bridging acetyl CoA and sugar binding sites (Fig. 6A). Other structural elements involving additional loops, N-termini, and C-termini further facilitate specific binding of substrates at both sites.

The prototypical member of the γ-carbonic carbonic anhydrase family, Cam, from *Methanosarcina thermophila* is differentiated from the other β-helix family members by a 3-histidine (3-His) divalent metal binding site (Suppl. Fig. 12A) (Iverson *et al.*, 2000). This metal site has been attributed to carbonic anhydrase reactivity. Yet not all homologs containing 3-His coordination, like RicA in Fig. 6B, are active in hydrating CO_2_ *in vitro* (Iverson *et al.*, 2000, Jeyakanthan *et al.*, 2008, Park *et al.*, 2012, Herrou & Crosson, 2013). The further annotated γ-carbonic anhydrase from *Brucella abortus*, RicA, can bind a polyether (PE5) in an acetyl-CoA-like binding pocket (Fig. 6B). A solvent exposed acetyl-CoA-like binding pocket is also found in the annotated γ-carbonic anhydrase PaaY from *Acinetobacter baumannii*, with the enzyme showing bifunctional carbonic anhydrase and thioesterase activities (Suppl. Fig. S12B) (Jiao *et al.*, 2023). Other β-helix family members like LpxA and the unique heterotrimeric complex of β-helix proteins in mitochondria (GAMMACA1, GAMMACA2, and GAMMACAL2) are missing a third histidine residue in the metal binding pocket and do not bind Zn at the GAMMACA2/ GAMMACAL2 dimer interface, yet both of these examples bind molecules in the acetyl-CoA-like binding pocket (Suppl. Fig. S12C,E). Notably, the GAMMACA1/GAMMACA2 interface has a Zn bound at a 3-His coordination stie. The heterotrimeric mitochondrial complex also illustrates how the variable loop near the active site (Loop2 in GAMMACA2, blue) can also mediate protein-protein interactions with another protein outside the trimeric core of β-helix proteins (Suppl. Fig. S12E). While all the γ-carbonic anhydrase annotated proteins have the 3-His metal site, variations in Loops1-2, the N-terminal loop, and the C-terminal helix may suggest different accessibility to the active site required for either carbonic anhydrase or other as of yet undescribed enzymatic activities (Fig. 6 and Suppl. Fig. S12).

GenBank accessions for Yes49-02 (JASEFG010000015) and Yes71-06b (JASEIM010000036) annotate the DNA sequence corresponding to FohE with two ORFs. The first ORF corresponding to FohE, is truncated in relation to Mic1c10 FohE, due to the deletion of a single nucleotide resulting in the introduction of a premature stop codon (Suppl. Fig. S13). Whereas the first ORF begins with a canonical ATG start codon, the second ORF is annotated as beginning with ATA.

Upon further analysis, we detected an earlier in-frame ATA codon leading to an extension of *fohE’* by 24 codons (Suppl. Fig. S13D). At the 5’ end of this alternative start site of *fohE’*, a 4-base overlap with FohE (ATAA) is reminiscent of the typical overlap of translationally coupled gene pairs. Importantly, the shorter FohE’ version (Suppl. Fig. S13B) would result in FohE/FohE’ heterodimers not being able to bind metals due to a missing third metal-chelating histidine residue (Suppl. Fig. 12D). The longer FohE’ version (Suppl. Fig. S13C) on the other hand could form a heterodimer with FohE and retain the metal-coordination site.

**3. Supplementary Tables**

**Supplementary Table S1: Strains of *M. maripaludis* which were considered for annotation and other analyses.**

Most strains received a strain-level taxID by NCBI. This was not done for few strains (listed in NCBI under the taxId of the type strain of the species, shown in parenthesis).

| **Species** | **Strain** | **NCBI taxId** | **Culture Collection** | **Replicons (accessions)**  **and UniProt proteomes (UP)** | **Genome size (bp)** | **%GC** | **Comment** | **Reference** |
| --- | --- | --- | --- | --- | --- | --- | --- | --- |
| *M. maripaludis* | Mic1c10 | (39152) | NBRC 105639 | Chr: CP186018.1  BAAABJ000000000.1 (38 contigs)  [UP001453044](https://www.uniprot.org/proteomes/UP001453044) | Chr: 1 771 462 | 33.0 | Isolated from oil storage tank | This study;  (Mori *et al.*, 2010) |
| *M. maripaludis* | KA1 | 637914 | NBRC 102054 | Chr: AP011526.1 Plas: AP011527.1 (pMMKA1)  [UP000264208](https://www.uniprot.org/proteomes/UP000264208) | Chr: 1 846 330 Plas: 12 084 | 32.9 | Isolated from oil storage tank | (Uchiyama *et al.*, 2010) |
| *M. maripaludis* | OS7 | 637915 | NBRC 103642 | Chr: AP011528.1  [UP000263689](https://www.uniprot.org/proteomes/UP000263689) | Chr: 1 749 749 | 32.9 | Isolated from oil storage tank | (Tsurumaru *et al.*, 2018) |
| *M. maripaludis* | JJ^T^ | 39152 | DSM 2067^T^ | Chr: CP026606.1  UP000239462 | Chr: 1 714 918 | 32.9 | Type strain of species | (Poehlein *et al.*, 2018) |
| „M. maripaludis“ | X1 | 1053692 | (uncultured) | Chr: CP002913.1  UP000008889 | Chr: 1 746 697 | 32.9 | Metagenome reconstruction; subsurface thermophilic saline oil reservoir | (Wang *et al.*, 2011) |
| *M. maripaludis* | S2 (LL) | 267377 | DSM 14266 | Chr: BX950229.1  UP000000590 | Chr: 1 661 137 | 33.1 | Isolated from salt marsh; also designated strain LL | (Hendrickson *et al.*, 2004)  (Lyu *et al.*, 2016) |
| *M. maripaludis* | C5 | (39152) | ATCC BAA-1333 | Chr: CP000609.1 Plas: CP000610.1  UP000000253 | Chr: 1 780 761 Plas: 8285 | 33.0 | Isolated from marsh sediment, Sapelo Island, GA, USA | (Whitman *et al.*, 1986)  (Keswani *et al.*, 1996) |
| *M. maripaludis* | C6 | 444158 | ATCC BAA-1332 | Chr: CP000867.1  UP000000791 | Chr: 1 744 193 | 33.4 | Isolated from marsh sediment, Sapelo Island, GA, USA | (Whitman *et al.*, 1986)  (Keswani *et al.*, 1996) |
| *M. maripaludis* | C7 | 426368 | ATCC BAA-1331 | Chr: CP000745.1  UP000002298 | Chr: 1 772 694 | 33.3 | Isolated from marsh sediment, Sapelo Island, GA, USA | (Whitman *et al.*, 1986)  (Keswani *et al.*, 1996) |

**Supplementary Table S2: Genomic relations between the analyzed *M. maripaludis* strains^a^**.

| **Strains** | **Mic10** | **KA1** | **OS7** | **S2** | **X1** | **JJ** | **C5** | **C6** | **C7** |
| --- | --- | --- | --- | --- | --- | --- | --- | --- | --- |
| **Mic10** |  | 86.7 | 85.6 | 73.7 | 84.2 | 63.4 | 35.0 | 35.4 | 34.3 |
| **KA1** | 98.4 [84.1] |  | 88.2 | 74.0 | 87.4 | 63.7 | 35.7 | 35.4 | 34.3 |
| **OS7** | 98.4 [84.7] | 98.6 [86.8] |  | 74.7 | 85.3 | 64.0 | 35.7 | 35.6 | 34.3 |
| **S2** | 96.6 [83.1] | 96.7 [83.9] | 96.7 [83.7] |  | 74.0 | 64.9 | 35.5 | 35.5 | 34.3 |
| **X1** | 98.1 [85.8] | 98.3 [84.4] | 98.2 [84.3] | 96.6 [79.6] |  | 63.5 | 35.0 | 35.4 | 34.2 |
| **JJ** | 94.9 [79.6] | 94.6 [76.0] | 95.1 [78.3] | 95.3 [81.3] | 94.9 [78.4] |  | 35.4 | 35.6 | 34.7 |
| **C5** | 87.6 [73.2] | 87.8 [72.4] | 88.0 [73.6] | 88.0 [77.2] | 87.8 [72.9] | 88.1 [73.0] |  | 43.4 | 40.4 |
| **C6** | 87.8 [73.9] | 87.6 [70.6] | 88.0 [72.4] | 88.2 [78.2] | 87.9 [73.4] | 88.0 [74.4] | 90.6 [78.4] |  | 42.1 |
| **C7** | 87.4 [72.7] | 87.3 [71.4] | 87.4 [73.6] | 87.6 [78.4] | 87.5 [73.1] | 87.7 [74.2] | 89.6 [76.5] | 90.4 [80.0] |  |

^a^Upper triangle: *in silico* DNA-DNA hybridization (DDH) values calculated with the Genome-to-Genome distance Calculator (GGDC) 2.1 at http://ggdc.dsmz.de/ggdc.php#. Lower triangle: ANIb (average nucleotide identity, BLASTn) values calculated at http://jspecies.ribohost.com/jspeiesws. Values in square brackets are the percentage of aligned nucleotides between the two chromosome sequences. Values which support placement into the same species as Mic1c10 are colored purple. Values which are close to the species cutoff but fall short of this are colored yellow. Unshaded cells (for strains C5, C6, and C7) contain values that indicate these strains represent distinct species. Strains designations are shown in the top row and leftmost column. Details of the analyzed strains are given in Suppl. Table S1.

**Supplementary Table S3: Oligonucleotides used to PCR amplify and sequence the MIC core island and its flanking regions of *Methanobacterium* IM1.**

See Fig. 2 for the MIC transposon in *Methanobacterium* IM1. A target DNA sequence of 2513 bp containing *fohB* and *fohA* genes (encoding the [NiFe]-hydrogenase small and large subunits) was PCR amplified using oligonucleotides 1048_F and 1030_R. Oligonucleotides 1049_R and 1061_F were used to amplify 2163 bp containing *fohD, tatA, tatC* and *fohE* DNA sequences. Oligonucleotides highlighted orange were used for sequencing the MIC core. Oligonucleotides highlighted in purple were used to amplify and sequence 1893 bp upstream of *fohB,* corresponding to a transposase sequence. Downstream of *fohE* 3 different but overlapping DNA fragments were generated via PCR, 1531 bp, 1455 bp and 3408 bp, using the following primer pairs FR5_F / FR6_R, FR7_F / FR8_R and FR9_F / FR10_R, respectively. Oligonucleotides highlighted in light blue were used for sequencing the 3 fragments downstream of *fohE*.

| **Oligonucleotide name** | **Sequence (5`-3`)** |
| --- | --- |
| 1048_F | GCATGATAATGCCAACTGGAAATG |
| 1030_R | CTATTCAGTGCCATCTGGGGTTTTA |
| 1029_F | ATGAAAAAAGAATTTAAAAAAATATTGGATCATCC |
| 1054_R | gtatcatgaccctgacatccaag |
| 1055_F | ctggtacatggtaactggcg |
| 1057_F | agttgaggcagcctatggca |
| 1059_F | gtcttccaacaaactacagttacacc |
| 1060_R | acaaaatcgccccatatgttcc |
| 1061_F | gcactgaatagtgccgtctct |
| 1063_F | cctgaaggggtccgtttgat |
| 1049_R | tgcaggaaccagcttaccatc |
| 1065_F | gattggtggactgggaatgc |
| Fb1_R | tgttcttctatctattggatgatc |
| Fb2_F | cggagatgaagttaaaaactac |
| FR5_F | ctgtgttatacttcacaatgc |
| FR6_R | gaagatatcctggcattaatg |
| FR7_F | gtggtcaattaccacgatttc |
| FR8_R | ccctgcaaaatttaaggcc |
| FR9_F | ccctctttaatggtgaaattaattaa |
| FR10_R | ctgcttattgcctttttctt |
| FR11_F | ctgagcacattttctatttacc |
| FR12_F | ctggtgagtataagcacatt |
| FR13_F | caatactaattctatggaccatat |

**Supplementary Table S4: Comparison of the pT26-2-like integrative elements from *M. maripaludis* strains Mic1c10 and X1.**

The pT26-2-like integrative element extends from MMic1c10_03460 to MMic1c10_03630 and most of the proteins have a homolog in strain X1 (locus tag prefix GYY). Sequence relationship between homologous proteins is indicated by color (green: >90%, blue: 60-90%, yellow: <60%). Strain-specific proteins are highlighted in red (dashes indicate that no data are available).

| **Mic1c10** | **length** | **X1** | **length** | **seq_id** |
| --- | --- | --- | --- | --- |
| 03460 | 174 | 03190 | 174 | 99% |
| 03465 | 287 | 03185 | 287 | 98% |
| 03470 | 664 | 03180 | 664 | 98% |
| 03475 | 448 | 03175 | 456 | 47% |
| 03480 | 972 | 03170 | 972 | 98% |
| 03485 | 480 | 03165 | 501 | 30% |
| - | - | 03160 | 436 | - |
| 03490 | 119 | 03155 | 122 | 42% |
| 03495 | 44 | 03150 | 44 | 81% |
| 03500 | 90 | 03145 | 90 | 91% |
| 03505 | 146 | 03140 | 146 | 98% |
| 03510 | 138 | 03135 | 138 | 100% |
| 03515 | 465 | 03130 | 465 | 100% |
| 03520 | 708 | 03125 | 707 | 95% |
| - | - | 03120 | 87 | - |
| 03525 | 78 | 03115 | 78 | 100% |
| 03530 | 186 | - | - | - |
| - | - | 03110 | 196 | - |
| 03532 | 46 | 03105 | 46 | 91% |
| 03535 | 86 | 03100 | 86 | 100% |
| 03540 | 116 | 03095 | 116 | 99% |
| 03545 | 120 | 03090 | 124 | 91% |
| 03550 | 81 | - | - | - |
| 03555 | 132 | - | - | - |
| - | - | 03085 | 49 | - |
| 03560 | 114 | 03080 | 120 | 81% |
| 03565 | 483 | 03075 | 486 | 86% |
| 03570 | 89 | 03070 | 74 | 83% |
| 03575 | 211 | 03065 | 211 | 80% |
| 03580 | 95 | 03060 | 94 | 80% |
| 03585 | 136 | - | - | - |
| - | - | 03055 | 141 | - |
| 03590 | 123 | 03050 | 121 | 70% |
| 03595 | 236 | 03045 | 233 | 85% |
| 03600 | 757 | 03040 | 737 | 61% |
| 03605 | 676 | 03035 | 675 | 92% |
| 03610 | 88 | - | - | - |
| 03615 | 51 | - | - | - |
| - | - | 03030 | 200 | - |
| - | - | 03025 | 43 | - |
| 03620 | 91 | 03020 | 51 | 98% |
| 03625 | 54 | 03015 | 106 | 93% |
| 03630 | 215 | 03010 | 54 | 77% |

**Supplementary Table S5: Comparison of the cas proteins between *M. maripaludis* strains Mic1c10, OS7, KA1, X1 and MIC098Bin5.**

CRISPR/cas genes in the five analyzed strains of *Methanococcus maripaludis*. Locus tag serial numbers are given for each strain (species-specific tags: Mic1c10: MMic1c10; OS7: MMOS7; X1: GYY; MIC098Bin5: H0S71; KA1: MMKA1), with protein length given for strain Mic1c10 and protein sequence identity (seq_id) given for strains OS7, X1, MIC098Bin5 and KA1 as compared to strain Mic1c10. All genes from strains OS7, X1 and MIC098Bin5 are highly similar to those from Mic1c10 (>=97% seq_id). Genes which are very closely related in strain KA1 are colored in green, those which are distant are colored in yellow. Gene *cas7* is annotated as *csh2* and *cas8b* as *csh1* in strains OS7 and KA1.

| **gene** | **Mic1c10** | **length** | **OS7** | **seq_id** | **X1** | **seq_id** | **MIC098Bin5** | **seq_id** | **KA1** | **seq_id** |
| --- | --- | --- | --- | --- | --- | --- | --- | --- | --- | --- |
| *cas1* | 05090 | 342 | 10410 | 97% | 05545 | 98% | 01360 | 97% | 10640 | 98% |
| *cas2* | 05095 | 96 | 10420 | 100% | 05550 | 100% | 01365 | 100% | 10650 | 100% |
| *cas3* | 05070 | 765 | 10370 | 99% | 05525 | 99% | 01340 | 99% | 10600 | 33% |
| *cas4* | 05100 | 209 | 10440 | 99% | 05555 | 97% | 01370 | 97% | 10660 | 97% |
| *cas5* | 05075 | 221 | 10380 | 99% | 05530 | 99% | 01345 | 99% | 10610 | 31% |
| *cas6* | 05065 | 217 | 10360 | 100% | 05520 | 100% | 01335 | 100% | 10590 | 100% |
| *cas7* | 05080 | 291 | 10390 | 100% | 05535 | 100% | 01350 | 100% | 10620 | 29% |
| *cas8b* | 05085 | 567 | 10400 | 99% | 05540 | 99% | 01355 | 100% | 10630 | 24% |

**Supplementary Table S6: Regions of high and low conservation in one of the S-layer proteins from the strains Mic1c10, OS7 and KA1.**

One of three paralogous genes for S-layer proteins (homolog 2 in Fig.4) shows a peculiar central sequence exchange due to a divSEG (replacement), thus combining highly conserved terminal sequences (N-term, C-term; >90% sequence identity; green) with a remarkably divergent central region (<50% sequence identity; orange, alignment in Suppl. Fig. S8). Because most breakpoints are strain-specific, all three pairwise comparisons are reported. The boundaries between N-terminal, middle, and C-terminal region were selected after identifying highly conserved N-terminal and C-terminal sequences via BLASTn and BLASTx analyses.

| compared strains | region | seq_id  [%] | strain | region | length | strain | region | length |
| --- | --- | --- | --- | --- | --- | --- | --- | --- |
| Mic1c10 vs KA1 | N-term | 96 | Mic1c10 | 1-96 | 96 | KA1 | 1-96 | 96 |
| Mic1c10 vs KA1 | middle | 49 | Mic1c10 | 97-399 | 303 | KA1 | 97-390 | 294 |
| Mic1c10 vs KA1 | C-term | 95 | Mic1c10 | 400-542 | 143 | KA1 | 391-533 | 143 |
| Mic1c10 vs OS7 | N-term | 100 | Mic1c10 | 1-85 | 85 | OS7 | 1-85 | 85 |
| Mic1c10 vs OS7 | middle | 42 | Mic1c10 | 86-434 | 349 | OS7 | 86-452 | 367 |
| Mic1c10 vs OS7 | C-term | 99 | Mic1c10 | 435-542 | 108 | OS7 | 453-560 | 108 |
| OS7 vs KA1 | N-term | 94 | OS7 | 1-99 | 99 | KA1 | 1-99 | 99 |
| OS7 vs KA1 | middle | 44 | OS7 | 100-452 | 353 | KA1 | 100-425 | 326 |
| OS7 vs KA1 | C-term | 98 | OS7 | 453-560 | 108 | KA1 | 426-533 | 108 |

**Supplementary Table S7: Predicted N-X-(S/T) sequons in S-layer proteins.**

| Surface Layer Protein Homolog | Protein | N-X-(S/T)  Sequons  (high-score) | Probability (%)  (>32%)^(a)^ | Group | N-X-(S/T)  Sequons  (low-score) | Probability (%)  ( <32%)^(b)^ | Group |
| --- | --- | --- | --- | --- | --- | --- | --- |
| 1 | GYY_05085 | 114 NFTS  189 NDSG  363 NNTL | 56  47  57 | (1b)  (1c)  (1d) |  |  |  |
|  | H0S71_00905 | 114 NFTS  189 NDSG  363 NNTL | 56  47  57 | (1b)  (1c)  (1d) |  |  |  |
|  | MMic1c10_04635 | 114 NFTS  189 NDSG  363 NNTL | 56  47  57 | (1b)  (1c)  (1d) |  |  |  |
|  | MMJJ_01180 | 46 NNSN  114 NFTS  189 NDSG  363 NNTL | 55  56  47  58 | (1a)  (1b)  (1c)  (1d) |  |  |  |
|  | MMKA1_09700 | 114 NFTS  189 NDSG  363 NNTL | 56  47  57 | (1b)  (1c)  (1d) |  |  |  |
|  | MMOS7_09490 | 114 NFTS  189 NDSG  363 NNTL | 56  47  57 | (1b)  (1c)  (1d) |  |  |  |
|  | MMP0875 | 46 NNSN | 55 | (1a) |  |  |  |

| Surface Layer Protein Homolog | Protein | N-X-(S/T)  Sequons  (high-score) | Probability (%)  (>32%)^(a)^ | Group^(d)^ | N-X-(S/T)  Sequons  (low-score) | Probability (%)  ( <32%)^(b)^ | Group^(d)^ |
| --- | --- | --- | --- | --- | --- | --- | --- |
| 2 | GYY_01985 | 105 NGTD  110 NVSL  186 NDSD  198 NDTI  348 NTTS | 78  65  51  42  60 | **(2a)**  **(2b)**  **(2i)**  **(2j)**  (2m) | 492 NESP | 9 | (2n) |
|  | H0S71_06800 | 135 NGTS  167 NNSN  212 NYTA | 65  57  56 | (2d)  (2g)  (2l) | 493 NESP | 9 | (2n) |
|  | MMic1c10_02110 | 141 NFTY  148 NNSL  187 NKSD  213 NATT | 55  62  59  47 | (2e)  (2f)  (2h)  (2k) | 502 NESP | 9 | (2n) |
|  | MMJJ_06560 | 132 NDSS  189 NDSD  201 NDTI | 69  55  45 | (2c)  **(2i)**  **(2j)** | 498 NESP | 9 | (2n) |
|  | MMKA1_04020 | 105 NGTA  110 NVSL  186 NDSD  198 NDTI | 76  75  51  42 | **(2a)**  **(2b)**  **(2i)**  **(2j)** | 493 NESP | 9 | (2n) |
|  | MMOS7_03960^(c)^ | - | - |  | 520 NESP | na | (2n) |
|  | MMP0383 | - | - |  | 535 NESP | na | (2n) |
| 3 | GYY_03630 | 480 NESK | 55 | (3a) | 488 NESP | 10 | (3b) |
|  | H0S71_07860 | 480 NESK | 55 | (3a) | 488 NESP | 10 | (3b) |
|  | MMic1c10_03015 | 480 NESK | 55 | (3a) | 488 NESP | 10 | (3b) |
|  | MMJJ_04150 | - | - |  | 488 NESP | na | (3b) |
|  | MMKA1_06170 | 480 NESK | 55 | (3a) | 488 NESP | 10 | (3b) |
|  | MMOS7_06120 | 480 NESK | 55 | (3a) | 488 NESP | 10 | (3b) |
|  | MMP0569 | - | - |  | 488 NESP | na | (3b) |

^(a)^The default probability threshold of NetNGlyc is 50%, as developed for predicting N-glycosylation in human proteins (Gupta & Brunak, 2002). For the current analysis of archaeal N-glycosylation sites (this table and Fig. 4), the cut-off was relaxed to the 32% threshold, alternatively provided by NetNGlyc. The probability median (56%) is only slightly above the 50% threshold, and several sites have a probability just below the threshold (47%, 45%, 42%). With the relaxed threshold, sequons from homologs 1 and 2, located within the variable region of interest in D1, are included in the analysis. These sequons are listed as high-score. ^(b)^Homologs 2 and 3 have a conserved sequon in D3 (NESP) that is low scoring and not counted in Fig. 4. The Pro in the third position after the Asn makes this sequon an unlikely N-glycosylation site (Gupta & Brunak, 2002). These are listed as low-score. NetNGlyc lists such low-scoring sequons only for proteins which have at least one higher-scoring sequon. Thus, for proteins exclusively having NESP, a probability value is not available (na). ^(c)^MMOS7_03960 has only the NESP tetrapeptide and no additional potential N-glycosylation site, contrary to a previous report (Kawaichi *et al.*, 2024).^(d)^The column “Group“ differentiates similar and dissimilar sequons. Grouping assigns the same letter to a sequon if it is aligned in Suppl. Fig. S9 (homolog 1), Suppl. Fig. S8 (homolog 2) or Suppl. Fig. S10 (homolog 3). Serial letters start with “a” for each homolog and are preceded by the homolog number. Bold text indicates sequons shared commonly between strains X1 and KA1, which map also to the same position in the protein structure prediction in Fig. 4.

**Supplementary Table S8: Methylation sites in the genome of *M. maripaludis* Mic1c10.**

| **Motif** | **Base #** | **Type** | **# motifs in genome** | **% motifs detected as methylated** | **Mean coverage** | **Mean IPD ratio** | **Objective score** |
| --- | --- | --- | --- | --- | --- | --- | --- |
| GATC | 2 | m6A | 5998 | 51.7 | 261 | 3.1 | 191 863 |
| CCCGA | 5 | m6A | 1272 | 99.9 | 260 | 5.4 | 415 964 |
| CAC-N6-RTTA / TAAY-N6-GTG | 2 3 | m6A m6A | 278 | 100 | 254 | 5.4 | 90 936 |

^a^m6A, N6-methyladenosine. IPD ration and Objective Score values as output from the Basemods tool of the SMRT^®^ Analysis Software

**Supplementary Table S9: Comparison of the chromosomes from *M. maripaludis* strains Mic1c10 and KA1.**

Due to extremely high similarity, only 72 segments are required to traverse all of the chromosome. These are serially numbered with a KA prefix (**serial**). Segments are classified into four categories (matchSEG, divSEG, repeatSEG, and crisprSEG) (**category**). A matchSEG is a contiguous region of very high sequence similarity (see Methods and Suppl. Text S2 for further details). Nucleotide sequence identity values (**seq_id**), the length (in bp) for each strain (**length**), and the genome position (**pos**) are provided. MatchSEGs range in size from 586 bp to 186 kb. A repeatSEG is a variant of a matching sequence, as this segment occurs as a tandem duplication in at least one of the strains. The number of copies and some further details are provided in the comment (**comment**). RepeatSEGs range in size from 79 bp to 31 kb. A divSEG is a genome region which is strain-specific. In many cases, there are unrelated sequences in the two strains, intercalated at an equivalent chromosome position. For each strain ("Mic1c10:";"KA1:"), the sequence is characterized by a term ("strain-specific";" short";"fewBases") or by a short description of key features. In few cases, divSEG sequences are highly similar (comment tagged Mic1c10+KA1; 80% or 84% seqid). The level of sequence identity is well below a matchSEG (typically >97% seqid). A divSEG may also be an indel which occurs in only one strain (a hyphen is given for length and position in the other strain). In some cases, the strain-specific sequence is bounded by a direct repeat ("<length> bp duplication with matchSEG <serial>"), one copy of which is absent in the other strain, together with the intervening sequence. The longest divSEG has 65 kb. Each strain has a CRISPR array, but the array in strain Mic1c10 is of hybrid nature. While the second part shares its CRISPR repeat with strain KA1 (CTAAAAGAATAACTTGCAAAATAACAAGCATTGAAAC), the first (and longer) part has a distinct but related CRISPR repeat (CTAAAAGAGTAAAATGCAATAGAACAAGCATTGAAAC). The repeats differ at 5 positions (see Suppl. Fig. S7B).

| **serial** | **category** | **Seq_id** | **Length**  **(Mic1c10)**  **[bp]** | **pos (Mic1c10)** | **length (KA1)**  **[bp]** | **pos (KA1)** | **comment** |
| --- | --- | --- | --- | --- | --- | --- | --- |
| KA01 | matchSEG | 99 | 168057 | 1-168057 | 168043 | 1-168043 | - |
| KA02 | repeatSEG | 98 | 31284 | 168058-199341,  199333-230616 | 31359 | 168044-199402 | Mic1c10: 2 copies, 1 point mutation, 9 bp overlap; KA1: 1 copy |
| KA03 | matchSEG | 99 | 116997 | 230617-347613 | 117040 | 199403-316442 | - |
| KA04 | divSEG | - | 5 | 347614-347618 | 938 | 316443-317380 | Mic1c10: fewBases; KA1: strain-specific |
| KA05 | matchSEG | 98 | 12746 | 347619-360364 | 12746 | 317381-330126 | - |
| KA06 | repeatSEG | 100 | 758 | 360365-361122 | 758 | 330127-330884, 330884-331641 | Mic1c10: 1 copy; KA1: 2 copies, identical, 1 bp overlap |
| KA07 | matchSEG | 98 | 22812 | 361123-383934 | 22810 | 331642-354451 | - |
| KA08 | repeatSEG | 94 | 488 | 383935-384422 | 487 | 354452-354938, 354937-355423 | Mic1c10: 1 copy; KA1: 2 copies, 2 point mutations, 2 bp overlap |
| KA09 | matchSEG | 96 | 586 | 384423-385008 | 586 | 355424-356009 | - |
| KA10 | divSEG | - | 1060 | 385009-386068 | 1092 | 356010-357101 | Mic1c10: strain-specific; KA1: strain-specific |
| KA11 | matchSEG | 91 | 1490 | 386069-387558 | 1496 | 357102-358597 | - |
| KA12 | divSEG | - | 15025 | 387559-402583 | 17573 | 358598-376170 | Mic1c10: strain-specific; KA1: strain-specific |
| KA13 | matchSEG | 97 | 13727 | 402584-416310 | 13729 | 376171-389899 | - |
| KA14 | divSEG | - | 909 | 416311-417219 | 882 | 389900-390781 | Mic1c10: strain-specific; KA1: strain-specific |
| KA15 | matchSEG | 99 | 41932 | 417220-459151 | 41884 | 390782-432665 | - |
| KA16 | divSEG | - | 2 | 459152-459153 | 2402 | 432666-435067 | Mic1c10: fewBases; KA1: strain-specific |
| KA17 | matchSEG | 98 | 5258 | 459154-464411 | 5259 | 435068-440326 | - |
| KA18 | divSEG | - | 2210 | 464412-466621 | 1685 | 440327-442011 | Mic1c10: strain-specific; KA1: strain-specific |
| KA19 | matchSEG | 98 | 24339 | 466622-490960 | 24338 | 442012-466349 | 54 bp duplication with matchSEG_KA21 (att site of pT26-2) |
| KA20 | divSEG | - | - | - | 25338 | 466350-491687 | KA1: strain-specific (pT26-2) |
| KA21 | matchSEG | 100 | 607 | 490907-491513 | 607 | 491688-492294 | 54 bp duplication with matchSEG_KA19 (att site of pT26-2); 7 bp duplication with matchSEG_KA23 |
| KA22 | divSEG | - | 750 | 491514-492263 | - | - | shows 99% seq_id to part of divSEG_KA36 from strain KA1 |
| KA23 | matchSEG | 98 | 18072 | 492264-510335 | 18062 | 492288-510349 | 7 bp duplication with matchSEG_KA21; 486 bp duplication with matchSEG_KA25 |
| KA24 | divSEG | - | 914 | 510336-511249 | - | - | Mic1c10: strain-specific |
| KA25 | matchSEG | 99 | 10608 | 511250-521857 | 10635 | 509864-520498 | 486 bp duplication with matchSEG_KA23 |
| KA26 | divSEG | - | 4288 | 521858-526145 | 6712 | 520499-527210 | Mic1c10: strain-specific; KA1: strain-specific |
| KA27 | matchSEG | 99 | 103704 | 526146-629849 | 103673 | 527211-630883 | - |
| KA28 | divSEG | - | 459 | 629850-630308 | 479 | 630884-631362 | Mic1c10: strain-specific; KA1: strain-specific |
| KA29 | matchSEG | 99 | 39589 | 630309-669897 | 39599 | 631363-670961 | - |
| KA30 | repeatSEG | 99 | 644 | 669898-670541,  670542-671185 | 640 | 670962-671601 | Mic1c10: 2 copies, identical, no overlap; KA1: 1 copy, last 4 bp lacking |
| KA31 | matchSEG | 99 | 7764 | 671186-678949 | 7764 | 671602-679365 | 20 bp duplication with matchSEG_KA33 |
| KA32 | divSEG | - | 29224 | 678950-708173 | - | - | Mic1c10: strain-specific (pT26-2-like int. element) |
| KA33 | matchSEG | 99 | 30313 | 708174-738486 | 30315 | 679346-709660 | 20 bp duplication with matchSEG_KA31 |
| KA34 | divSEG | - | 3022 | 738487-741508 | 36 | 709661-709696 | Mic1c10: strain-specific; KA1: short |
| KA35 | matchSEG | 99 | 55843 | 741509-797351 | 55912 | 709697-765608 | 56 bp duplication with matchSEG_KA37 |
| KA36 | divSEG | - | - | - | 65800 | 765609-831408 | KA1: strain-specific except for a 754 bp region with 99% seq_id to divSEG_KA22 from strain Mic1c10 |
| KA37 | matchSEG | 99 | 42869 | 797296-840164 | 42856 | 831409-874264 | 56 bp duplication with matchSEG_KA35; 464 bp duplication with matchSEG_KA41 |
| KA38 | divSEG | - | - | - | 5788 | 874265-880052 | KA1: strain-specific (except for a 565 bp region with high similarity) |
| KA39 | matchSEG | 99 | 50861 | 839701-890561 | 50990 | 880053-931042 | 464 bp duplication with matchSEG_KA37 |
| KA40 | divSEG | - | - | - | 259 | 931043-931301 | KA1: strain-specific |
| KA41 | matchSEG | 99 | 7745 | 890562-898306 | 7745 | 931302-939046 | - |
| KA42 | repeatSEG | 100 | 201 | 898307-898507,  898508-898708 | 201 | 939047-939247 | Mic1c10: 2 copies, identical, no overlap; KA1: 1 copy |
| KA43 | matchSEG | 99 | 86292 | 898709-985000 | 86320 | 939248-1025567 | - |
| KA44 | divSEG | - | 5520 | 985001-990520 | 6159 | 1025568-1031726 | Mic1c10: strain-specific; KA1: strain-specific |
| KA45 | matchSEG | 96 | 2314 | 990521-992834 | 2314 | 1031727-1034040 | - |
| KA46 | crisprSEG | - | 5188 | 992835-998022 | - | - | CRISPR repeat: CTAAAAGAGTAAAATGCAATAGAACAAGCATTGAAAC |
| KA47 | crisprSEG | - | 2581 | 998023-1000603 | 5878 | 1034041-1039918 | CRISPR repeat: CTAAAAGAATAACTTGCAAAATAACAAGCATTGAAAC |
| KA48 | matchSEG | 99 | 25423 | 1000604-1026026 | 25423 | 1039919-1065341 | - |
| KA49 | divSEG | - | - | - | 1462 | 1065342-1066803 | KA1: shows 92% seq_id to matchSEG_KA50 over 1410 bp |
| KA50 | matchSEG | 99 | 40393 | 1026027-1066419 | 40384 | 1066804-1107187 | - |
| KA51 | divSEG | - | 2503 | 1066420-1068922 | 2502 | 1107188-1109689 | Mic1c10+KA1: there is 84% seq_id over most of this region |
| KA52 | matchSEG | 98 | 1412 | 1068923-1070334 | 1412 | 1109690-1111101 | - |
| KA53 | divSEG | - | 4203 | 1070335-1074537 | 4212 | 1111102-1115313 | Mic1c10+KA1: there is 80% seq_id over most of this region |
| KA54 | matchSEG | 99 | 59695 | 1074538-1134232 | 59691 | 1115314-1175004 | - |
| KA55 | divSEG | - | 119 | 1134233-1134351 | 3294 | 1175005-1178298 | Mic1c10: strain-specific; KA1: strain-specific |
| KA56 | matchSEG | 99 | 40246 | 1134352-1174597 | 40279 | 1178299-1218577 |  |
| KA57 | divSEG | - | 2936 | 1174598-1177533 | 5 | 1218578-1218562 | Mic1c10: strain-specific (except for a 528 bp region with high similarity) |
| KA58 | matchSEG | 96 | 11674 | 1177534-1189207 | 11665 | 1218563-1230227 | 15 bp duplication with matchSEG_KA58 |
| KA59 | divSEG | - | 6186 | 1189208-1195393 | 2184 | 1230228-1232411 | Mic1c10: strain-specific; KA1: strain-specific |
| KA60 | matchSEG | 97 | 97835 | 1195394-1293228 | 97843 | 1232412-1330254 | - |
| KA61 | repeatSEG | 100 | 345 | 1293229-1293573,  1293574-1293918,  1293919-1294299 | 345 | 1330255-1330599, 1330600-1330944 | Mic1c10: 3 copies, 98%/93% seq_id, no overlap; KA1: 2 copies, 98% seq_id, no overlap |
| KA62 | matchSEG | 98 | 34815 | 1294300-1329114 | 34813 | 1330945-1365757 | - |
| KA63 | repeatSEG | 99 | 11940 | 1329115-1341054 | 11905 | 1365758-1377662, 1377663-1389567, 1389568-1401472, 1401473-1413412 | Mic1c10: 1 copy; KA1: 4 copies, 99-100% seq_id, no overlap |
| KA64 | matchSEG | 99 | 99875 | 1341055-1440929 | 99874 | 1413413-1513286 | - |
| KA65 | repeatSEG | 100 | 79 | 1440930-1441008 | 79 | 1513287-1513365, 1513366-1513444, 1513445-1513523 | Mic1c10: 1 copy; KA1: 3 copies, 1 point mutation, no overlap |
| KA66 | matchSEG | 99 | 145658 | 1441009-1586666 | 145800 | 1513524-1659323 | - |
| KA67 | divSEG | - | 46 | 1586667-1586712 | 1528 | 1659324-1660851 | Mic1c10: strain-specific; KA1: strain-specific |
| KA68 | matchSEG | 99 | 118837 | 1586713-1705549 | 118845 | 1660852-1779696 | - |
| KA69 | divSEG | - | - | - | 861 | 1779697-1780557 | KA1: strain-specific |
| KA70 | matchSEG | 97 | 14571 | 1705550-1720120 | 14569 | 1780558-1795126 | - |
| KA71 | divSEG | - | 1790 | 1720121-1721910 | 1718 | 1795127-1796844 | Mic1c10+KA1: strain-specific in both strains except for 500 bp (2 sections) with 90% seq_id |
| KA72 | matchSEG | 98 | 49552 | 1721911-1771462 | 49486 | 1796845-1846330 | - |

**Supplementary Table S10: Comparison of the chromosomes from *M. maripaludis* strains Mic1c10 and OS7.**

Due to extremely high similarity, only 67 segments are required to traverse all of the chromosome. These are serially numbered with an OS prefix (**serial**). Segments are classified into four categories (matchSEG, divSEG, repeatSEG, and crisprSEG) (**category**). A matchSEG is a contiguous region of very high sequence similarity (see Methods and Suppl. Text S2 for further details). Nucleotide sequence identity values (**seq_id**), the length (in bp) for each strain (**length**), and the genome position (**pos**) are provided. MatchSEGs range in size from 423 bp to 197 kb. A repeatSEG is a variant of a matching sequence, as this segment occurs as a tandem duplication in at least one of the strains. The number of copies and some further details are provided in the comment (**comment**). One repeatSEG shows no difference between Mic1c10 and OS7 (OS39) but there is only a single copy in strain KA1. RepeatSEGs range in size from 201 bp to 31 kb. The latter, repeatSEG_OS06 occurs as two contiguous 31 kb sequences in strain Mic1c10 but as a single interrupted sequence in strain OS7. A divSEG is a genome region which is strain-specific. In many cases, there are unrelated sequences in the two strains, intercalated at an equivalent chromosome position. For each strain ("Mic1c10:";"OS7:"), the sequence is characterized by a term ("strain-specific";" short";"fewBases") or by a short description of key features. In few cases, divSEG sequences are highly similar (comment tagged Mic1c10+OS7; 80% or 83% seqid). The level of sequence identity is well below a matchSEG (typically >97% seqid). A divSEG may also be an indel which occurs in only one strain (a hyphen is given for length and position in the other strain). In some cases, the strain-specific sequence is bounded by a direct repeat ("<length> bp duplication with matchSEG <serial>"), one copy of which is absent in the other strain, together with the intervening sequence. The longest divSEG has 66 kb. Each strain has a CRISPR array, which is of hybrid nature. While the second part shares its CRISPR repeat with strain KA1 (CTAAAAGAATAACTTGCAAAATAACAAGCATTGAAAC), the first (and longer) part has a distinct but related CRISPR repeat (CTAAAAGAGTAAAATGCAATAGAACAAGCATTGAAAC), shared between Mic1c10 and OS7. The repeats differ at 5 positions (see Suppl. Fig. S7B).

| **serial** | **category** | **Seq_id** | **length (Mic1c10)**  **[bp]** | **pos (Mic1c10)** | **length (OS7)**  **[bp]** | **pos (OS7)** | **comment** |
| --- | --- | --- | --- | --- | --- | --- | --- |
| OS01 | matchSEG | 99 | 27870 | 1-27870 | 27863 | 1-27863 | 11 bp duplication with matchSEG_OS03 |
| OS02 | divSEG | - | - | - | 18435 | 27864-46298 | OS7: strain-specific |
| OS03 | matchSEG | 99 | 11921 | 27860-39780 | 11921 | 46299-58219 | 11 bp duplication with matchSEG_OS01 |
| OS04 | divSEG | - | 158 | 39781-39938 | 515 | 58220-58734 | Mic1c10: strain-specific; OS7: strain-specific |
| OS05 | matchSEG | 98 | 128119 | 39939-168057 | 128117 | 58735-186851 | - |
| OS06 | repeatSEG | 98 | 27214  +4066 | 168058-195271, 195276-199341, 199333-230616 | 27209  +4066 | 186852-214060, 215481-219546 | Mic1c10: 2 copies, 31284 bp, 1 point mutation, 9 bp overlap; OS7: 1 copy with an insert (divSEG_OS07) at 27 kb |
| OS07 | divSEG | - | 4 | 195272-195275 | 1420 | 214061-215480 | Mic1c10: fewBases; OS7: strain-specific, inserted into repeatSEG_OS06 |
| OS08 | matchSEG | 98 | 116994 | 230617-347610 | 117010 | 219547-336556 | - |
| OS09 | divSEG | - | - | - | 932 | 336557-337488 | OS7: strain-specific |
| OS10 | matchSEG | 98 | 27836 | 347611-375446 | 27839 | 337489-365327 | - |
| OS11 | divSEG | - | 27310 | 375447-402756 | 180 | 365328-365507 | Mic1c10: strain-specific; OS7: strain-specific |
| OS12 | matchSEG | 96 | 13520 | 402757-416276 | 13509 | 365508-379016 | - |
| OS13 | divSEG | - | 1057 | 416277-417333 | 1111 | 379017-380127 | Mic1c10: strain-specific; OS7: strain-specific |
| OS14 | matchSEG | 98 | 423 | 417334-417756 | 423 | 380128-380550 | - |
| OS15 | divSEG | - | 117 | 417757-417873 | 620 | 380551-381170 | Mic1c10: strain-specific; OS7: strain-specific |
| OS16 | matchSEG | 98 | 41278 | 417874-459151 | 41079 | 381171-422249 | - |
| OS17 | divSEG | - | - | - | 2400 | 422250-424649 | OS7: strain-specific |
| OS18 | matchSEG | 98 | 5087 | 459152-464238 | 5086 | 424650-429735 | - |
| OS19 | divSEG | - | 4310 | 464239-468548 | - | - | Mic1c10: strain-specific |
| OS20 | matchSEG | 98 | 22412 | 468549-490960 | 22444 | 429736-452179 | 54 bp duplication with matchSEG_OS22 (att site of pT26-2) |
| OS21 | divSEG | - | - | - | 23645 | 452180-475824 | OS7: strain-specific (pT26-2) |
| OS22 | matchSEG | 96 | 19429 | 490907-510335 | 19422 | 475825-495246 | 54 bp duplication with matchSEG_OS20 (att site of pT26-2); 486 bp duplication with matchSEG_OS24 |
| OS23 | divSEG | - | 914 | 510336-511249 | - | - | Mic1c10: strain-specific |
| OS24 | matchSEG | 98 | 118600 | 511250-629849 | 118593 | 494761-613353 | 486 bp duplication with matchSEG_OS22 |
| OS25 | divSEG | - | 459 | 629850-630308 | 479 | 613354-613832 | Mic1c10: strain-specific; OS7: strain-specific |
| OS26 | matchSEG | 98 | 39589 | 630309-669897 | 39606 | 613833-653438 | - |
| OS27 | repeatSEG | 99 | 644 | 669898-670541, 670542-671185 | 640 | 653439-654078 | Mic1c10: 2 copies, identical, no overlap; OS7: 1 copy, last 4 bp lacking |
| OS28 | matchSEG | 99 | 7764 | 671186-678949 | 7764 | 654079-661842 | 20 bp duplication with matchSEG_OS30 |
| OS29 | divSEG | - | 29224 | 678950-708173 | - | - | Mic1c10: strain-specific (pT26-like int. element) |
| OS30 | matchSEG | 99 | 20758 | 708174-728931 | 20758 | 661823-682580 | 20 bp duplication with matchSEG_OS28 |
| OS31 | divSEG | - | 569 | 728932-729500 | 113 | 682581-682693 | Mic1c10: strain-specific; OS7: strain-specific |
| OS32 | matchSEG | 99 | 9000 | 729501-738500 | 8996 | 682694-691689 | - |
| OS33 | divSEG | - | 3000 | 738501-741500 | 18 | 691690-691707 | Mic1c10: strain-specific; OS7: short |
| OS34 | matchSEG | 98 | 55851 | 741501-797351 | 55905 | 691708-747612 | 53 bp duplication with matchSEG_OS36 |
| OS35 | divSEG | - | - | - | 66407 | 747613-814019 | OS7: strain-specific |
| OS36 | matchSEG | 99 | 71889 | 797299-869187 | 72013 | 814020-886032 | 53 bp duplication with matchSEG_OS34 |
| OS37 | divSEG | - | 1091 | 869188-870278 | 896 | 886033-886928 | Mic1c10: strain-specific; OS7: strain-specific |
| OS38 | matchSEG | 99 | 28028 | 870279-898306 | 28031 | 886929-914959 | - |
| OS39 | repeatSEG | 100 | 201 | 898307-898507, 898508-898708 | 201 | 914960-915160, 915161-915361 | Mic1c10: 2 copies, identical, no overlap; OS7: 2 copies, identical, no overlap; categorized as repeatSEG as strain KA1 contains a single copy. |
| OS40 | matchSEG | 98 | 33533 | 898709-932241 | 33551 | 915362-948912 | - |
| OS41 | divSEG | - | - | - | 325 | 948913-949237 | OS7: strain-specific |
| OS42 | matchSEG | 99 | 60814 | 932021-992834 | 60804 | 949238-1010041 | - |
| OS43 | crisprSEG | - | 5188 | 992835-998022 | 5787 | 1010042-1015828 | CRISPR repeat: CTAAAAGAGTAAAATGCAATAGAACAAGCATTGAAAC |
| OS44 | crisprSEG | - | 2581 | 998023-1000603 | 2654 | 1015829-1018482 | CRISPR repeat: CTAAAAGAATAACTTGCAAAATAACAAGCATTGAAAC |
| OS45 | matchSEG | 99 | 12657 | 1000604-1013260 | 12702 | 1018483-1031184 | 126 bp duplication with matchSEG_OS47 |
| OS46 | divSEG | - | 41361 | 1013261-1054621 | - | - | Mic1c10: strain-specific |
| OS47 | matchSEG | 96 | 11798 | 1054622-1066419 | 11791 | 1031059-1127426 | 126 bp duplication with matchSEG_OS45 |
| OS48 | divSEG | - | 2503 | 1066420-1068922 | 2502 |  | Mic1c10+OS7: there is 83% seq_id over most of this region |
| OS49 | matchSEG | 97 | 1412 | 1068923-1070334 | 1412 |  |  |
| OS50 | divSEG | - | 4203 | 1070335-1074537 | 4212 |  | Mic1c10+OS7: there is 80% seq_id over most of this region |
| OS51 | matchSEG | 98 | 158655 | 1074538-1233192 | 158620 | 1050976-1209595 |  |
| OS52 | divSEG | - | 5553 | 1233193-1238745 | 8 | 1209596-1209603 | Mic1c10: strain-specific; OS7: fewBases |
| OS53 | matchSEG | 96 | 15083 | 1238746-1253828 | 15095 | 1209604-1224698 | - |
| OS54 | divSEG | - | 34 | 1253829-1253862 | 567 | 1224699-1225265 | Mic1c10: short; OS7: strain-specific |
| OS55 | matchSEG | 97 | 23786 | 1253863-1277648 | 23789 | 1225266-1249054 | 68 bp duplication with matchSEG_OS55 |
| OS56 | divSEG | - | - | - | 4036 | 1249055-1253090 | OS7: strain-specific |
| OS57 | matchSEG | 99 | 15648 | 1277581-1293228 | 15649 | 1253091-1268739 | 68 bp duplication with matchSEG_OS53 |
| OS58 | repeatSEG | 97 | 345 | 1293229-1293573,  1293574-1293918,  1293919-1294299 | 345 | 1268740-1269084, 1269085-1269465 | Mic1c10: 3 copies, 98%/93% seq_id, no overlap; OS7: 2 copies, 93% seq_id, no overlap |
| OS59 | matchSEG | 98 | 197700 | 1294300-1491999 | 197872 | 1269466-1467337 | - |
| OS60 | repeatSEG | 99 | 753 | 1492000-1492752 | 753 | 1467338-1468090, 1468091-1468843 | Mic1c10: 1 copy; OS7: 2 copies, identical, no overlap |
| OS61 | matchSEG | 98 | 93927 | 1492753-1586679 | 93903 | 1468844-1562746 | - |
| OS62 | divSEG | - | 33 | 1586680-1586712 | 1515 | 1562747-1564261 | Mic1c10: short; OS7: strain-specific |
| OS63 | matchSEG | 98 | 118837 | 1586713-1705549 | 118727 | 1564262-1682988 | - |
| OS64 | divSEG | - | - | - | 861 | 1682989-1683849 | OS7: strain-specific |
| OS65 | matchSEG | 98 | 14571 | 1705550-1720120 | 14564 | 1683850-1698413 | - |
| OS66 | divSEG | - | 1057 | 1720121-1721177 | 1060 | 1698414-1699473 | Mic1c10+OS7: strain-specific in both strains except for 300 bp with 86% seq_id |
| OS67 | matchSEG | 98 | 50285 | 1721178-1771462 | 50276 | 1699474-1749749 | - |

**Supplementary Table S11: The regions from *M. maripaludis* Mic1c10 in an integrated comparison to those from strains KA1 and OS7.**

**A**: Legend for position, serial and category: The table lists the position (“**position**”) of all junctions encountered in the strain Mic1c10 genome when compared to the genomes of strains KA1 and OS7. The serial of the corresponding region is given (“**serial**”) as listed in Table S9 (KA1) and Table S10 (OS7). Also, the category is indicated (“**category**”) ([1] “M”: matchSEG; [2] “D”: divSEG if there are assigned bases in strain Mic1c10; [3] “d”: divSEG in case of an insert in strains KA1 or OS7, with a dummy (“-“, hyphen) in the position column; [4] “R”: repeatSEG if the region is repeated in strain Mic1c10 (in which case the individual copies are tagged by serial letters A, B, C, or, in case of a split duplication Aa, Ab); [5] “r”: repeatSEG if there is only a single copy in strain Mic1c10; [6] “C”: crisprSEG). Positions and serials are highlighted by a color-code ([1] matchSEG: serials not highlighted; positions yellow, light yellow for start position, dark yellow for end position; [2] divSEG which occur in strain Mic1c10: serial in blue for regions which deviate in only one of the comparison strains, and in green for regions which deviate in both comparison strains. Start positions in light blue or light green, end positions in dark blue or dark green; [3] divSEG which in strain Mic1c10 is absent or has only a short remnant (tagged fewBases or short): serials in red, positions in grey; [4] repeatSEG: for the first or best-matching copy, the same coloring as for matchSEG is used (see above), copies that are missing in the comparison strain are colored pink, position in light pink at start and dark pink at end). Typically, breakpoints which occur in both comparison strains match to an identical position but in few cases the alignment termini are shifted due to sequence differences. Such differing positions are highlighted in bold. Typically, a segment starts at the next base after the preceding segments. This may not be the case if deletions are flanked by a duplication, leading to position jump-back (highlighted by underscore).

**B**: Legend for proteins: Locus tags are used to reference proteins (tags: strain Mic1c10: “MMic1c10_”, strain KA1: “MMKA1_”, strain OS7: “MMOS7_”). The step size for serial numbers of adjacent genes is 5 for strain Mic1c10 (00005, 00010, 00015…) and 10 for strains KA1 and OS7 (00010, 00020, 00030…). Locus tags, if given for strains KA1 and OS7, refer to orthologs. The absence of an ortholog is indicated by “NP” (not present). The term notAnno indicates that the gene exists (as revealed by tBLASTn analysis) but has not been annotated in the genome. The extension “(nf)” indicates that the gene is disrupted (nonfunctional; this may be unrelated to the event leading to the junction). The term notAnno(nf) indicates a remnant of a gene, which is not annotated. For strain Mic1c10, the first protein at the start of a segment is given (light grey highlighting) and the last protein at the end of a segment (dark grey highlighting). For short segments, or those representing a single gene, only one protein is given with a dummy (dot character) at the other segment end. In case of a divSEG or repeatSEG, genes which are specific for strains KA1 and/or OS7 are indicated by the term “extra” (with blue-grey shading).

For each protein, it is specified if the coding region traverses the junction ([1]: hyphen (“-“): CDS does not traverse the junction; [2]: “+” indicates that extended parts of the CDS are encoded on both sides of the junction; in this case, its code is given twice (highlighted by underlining); also, the part coding for the N-terminal (“Nt”), C-terminal (“Ct”), and eventually central (“ctr”) region is indicated; [3] “r” indicates a CDS traversing a junction between a matchSEG and a repeatSEG; protein sequences are typically unaffected by this type of junction; [4] Nt: in one case, the gene is disrupted by truncation at the junction, thus lacking a few N-terminal codons; the gene is flagged “(nf)” while the complete genes from the other strains are flagged “(ok)”; [5] Ct: a few C-terminal codons are encoded on the adjacent segment; the orthologs thus differ in the extreme C-terminal region). Proteins encoded across a matchSEG to divSEG junction may be very closely related in the region encoded on the matchSEG but may be only distantly related (or even unrelated) in the region encoded on the divSEG. In the latter case, the region indicator (e.g. Ct) is highlighted by light-green shading for the protein from strain Mic1c10 and grey-blue shading by the deviating homolog; if all three strains have a copy of the gene, the highly similar pair has the same color. If the protein from a strain lacks a region that is present in the other strains, this is indicated by “NP” (**n**ot **p**resent). Special cases: (a) A special case is MMic1c10_01925 where an ortholog exists only in strain KA1; the gene is affected by a frameshift, leading to annotation of two independent genes (indicated as 03610/03600). (b) A special case is a translocated gene pair which is encoded on the 750 bp divSEG_KA22 in strain Mic1c10 and on the 65.8 kb divSEG_KA36 in strain KA1 (the KA1 locus tags are highlighted bold with red font; this is a toxin-antitoxin pair). (c) A special case is a gene which occurs as a long form in strain KA1 (parts are flagged Nt and Ct), while strains Mic1c10 and OS7 have a short form (flagged shrt), for which it is uncertain if it is atypically short or disrupted. (d) As a special case, strain Mic1c10 has an adjacent pair of close paralogs (MMic1c10_02555, MMic1c10_02560; 70% protein sequence identity). The N-terminal regions are encoded on part of a highly conserved 486 bp duplication (90% nucleotide sequence identity). Strains KA1 and OS7 have an ortholog of MMic1c10_02560 (MMKA1_05260, MMOS7_05210; encoded on the beginning of matchSEG_KA25/OS24). These strains lack an ortholog to MMic1c10_02555, indicated by “NP” for divSEG_KA24/OS23 and as “NP(dup)” at the end of matchSEG_KA23/OS22, which carries the 486 bp duplication. (e) As a special case, MMic1c10_05910 lacks a start codon (starting with Lys-3 of a close homolog) but this is unrelated to its position close to the beginning of divSEG_KA57. Strain KA1 does not have an ortholog (“NP”). Strain OS7 also lacks the start codon and has an additional frameshift; the N-terminal fragment is MMOS7_11780 (translated from an internal ATG), the C-terminal fragment is MMOS7_11870. This is specified as “11780/70”, highlighted in red.

| **category** | **serial for KA1** | **position (Mic1c10)**  **at KA1 junction** | **position (Mic1c10)**  **at OS7 junction** | **serial for OS7** | **category** | **locus tag serial Mic1c10**  **(MMic1c10_)** | **junction** | **locus tag serial KA1**  **(MMKA1_)** | **locus tag serial OS7**  **(MMOS7_)** |
| --- | --- | --- | --- | --- | --- | --- | --- | --- | --- |
| M | KA01 | 1 | 1 | OS01 | M | 00005 | - | 00010 | 00010 |
| M | KA01 |  | 27870 | OS01 | M | 00125 | - | notAnno | 00240 |
| - | - |  | - | OS02 | d | - |  | - | extra |
| M | KA01 |  | **27860** | OS03 | M | 00130 | - | notAnno | 00500 |
| M | KA01 |  | 39780 | OS03 | M | 00145 | - | 00300 | 00530 |
| M | KA01 |  | 39781 | OS04 | D | - |  | - | extra |
| M | KA01 |  | 39938 | OS04 | D | 00150(Ct) | + | 00310(Ct) | 00550(Ct) |
| M | KA01 |  | 39939 | OS05 | M | 00150(Nt) | + | 00310(Nt) | 00550(Nt) |
| M | KA01 | 168057 | 168057 | OS05 | M | 00815(Ct) | r | 01650(Ct) | 01910(Ct) |
| R | KA02A | 168058 | 168058 | OS06Aa | R | 00815(Nt) | r | 01650(Nt) | 01910(Nt) |
| R | KA02A |  | 195271 | OS06Aa | R | 00955(nf)(Nt) | + | 01930(Nt) | 02190(Nt) |
| - | - |  | 195272 | OS07 | d | 00955(nf)(Ct) | + | 01930(Ct) | 02190(Ct) |
| - | - |  | 195275 | OS07 | d | NP |  | NP | extra |
| R | KA02A |  | 195276 | OS06Ab | R | 00960 | - | 01940 | 02210 |
| R | KA02A | 199341 | 199341 | OS06Ab | R | 00975(nf)(Ct) | + | NP | NP |
| R | KA02B | **199333** | **199333** | OS06B | R | 00975(nf)(Nt) | + | NP | NP |
| R | KA02B | 230616 | 230616 | OS06B | R | 01135(nf)(Ct) | r | 01970(Ct) | 02240(Ct) |
| M | KA03 | 230617 | 230617 | OS08 | M | 01135(nf)(Nt) | r | 01970(Nt) | 02240(Nt) |
| M | KA03 | 347613 | 347610 | OS08 | M | 01770 | - | 03250 | 03520 |
| d | KA04 | 347614 | - | OS09 | d | - |  | extra | extra |
| d | KA04 | 347618 | - | OS09 | d | . |  | . | . |
| M | KA05 | 347619 | 347611 | OS10 | M | 01775 | - | 03270 | 03540 |
| M | KA05 | 360364 |  | OS10 | M | 01835 | - | 03390 | 03660 |
| r | KA06 | 360365 |  | OS10 | M | - |  | extra | - |
| r | KA06 | 361122 |  | OS10 | M | 01840 | r | 03410 | 03670 |
| M | KA07 | 361123 |  | OS10 | M | 01845 | - | 03420 | 03680 |
| M | KA07 |  | 375446 | OS10 | M | 01920 | - | 03590 | 03830 |
| M | KA07 |  | 375447 | OS11 | D | 01925 | - | 03610/03600 | NP |
| M | KA07 | 383934 |  | OS11 | D | 01965(Nt) | r | 03690(Nt) | NP |
| r | KA08 | 383935 |  | OS11 | D | 01965(Ct) | r | 03690(Ct) | NP |
| r | KA08 | 384422 |  | OS11 | D | 01970(Nt) | r | 03710(Nt) | NP |
| M | KA09 | 384423 |  | OS11 | D | . |  | . | . |
| M | KA09 | 385008 |  | OS11 | D | 01970(ctr) | r | 03710(ctr) | NP |
| D | KA10 | 385009 |  | OS11 | D | 01970(Ct) | + | 03710(Ct) | NP |
| D | KA10 | 386068 |  | OS11 | D | . |  | . | . |
| M | KA11 | 386069 |  | OS11 | D | 01975 | - | 03720 | NP |
| M | KA11 | 387558 |  | OS11 | D | . |  | . | . |
| D | KA12 | 387559 |  | OS11 | D | 01980 | - | extra | NP |
| D | KA12 | 402583 |  | OS11 | D | 02045 | Nt | notAnno | NP |
| M | KA13 | 402584 |  | OS11 | D | . |  | . | . |
| M | KA13 |  | 402756 | OS11 | D | . |  | . | . |
| M | KA13 |  | 402757 | OS12 | M | 02050 | - | 03900 | 03840 |
| M | KA13 |  | 416276 | OS12 | M | 02110(Nt) | + | 04020(Nt) | 03960(Nt) |
| M | KA13 |  | 416277 | OS13 | D | . |  | . | . |
| M | KA13 | 416310 |  | OS13 | D | 02110(ctr) | + | 04020(ctr) | 03960(ctr) |
| D | KA14 | 416311 |  | OS13 | D | 02110(ctr) | + | 04020(ctr) | 03960(ctr) |
| D | KA14 | 417219 |  | OS13 | D | . |  | . | . |
| M | KA15 | 417220 |  | OS13 | D | 02110(ctr) | + | 04020(ctr) | 03960(ctr) |
| M | KA15 |  | 417333 | OS13 | D | . |  | . | . |
| M | KA15 |  | 417334 | OS14 | M | 02110(Ct) | + | 04020(Ct) | 03960(Ct) |
| M | KA15 |  | 417756 | OS14 | M | . |  | . | . |
| M | KA15 |  | 417757 | OS15 | D | . |  | . | extra |
| M | KA15 |  | 417873 | OS15 | D | . |  | . | . |
| M | KA15 |  | 417874 | OS16 | M | 02115 | - | 04030 | 03990 |
| M | KA15 | 459151 | 459151 | OS16 | M | 02310 | - | 04410 | 04370 |
| d | KA16 | 459152 | - | OS17 | d | - |  | extra | extra |
| d | KA16 | 459153 | - | OS17 | d | notAnno | Ct | 04430 | 04390 |
| M | KA17 | 459154 | 459152 | OS18 | M | 02315(nf) | - | 04440(nf) | 04400(nf) |
| M | KA17 |  | 464238 | OS18 | M | 02340 | - | 04500 | 04460 |
| M | KA17 |  | 464239 | OS19 | D | . |  | . | . |
| M | KA17 | 464411 |  | OS19 | D | 02345(Nt) | + | 04510(Nt) | NP |
| D | KA18 | 464412 |  | OS19 | D | 02345(ctr) |  | 04510(ctr) | NP |
| D | KA18 | 466621 |  | OS19 | D | . |  | . | . |
| M | KA19 | 466622 |  | OS19 | D | 02345(Ct) | + | 04510(Ct) | NP |
| M | KA19 |  | 468548 | OS19 | D | . |  | . | . |
| M | KA19 |  | 468549 | OS20 | M | 02350 | - | 04520 | 04470 |
| M | KA19 | 490960 | 490960 | OS20 | M | 02490 | - | 04810 | 04760 |
| d | KA20 | - | - | OS21 | d |  |  | extra | extra |
| M | KA21 | **490907** | **490907** | OS22 | M | . |  | . | . |
| M | KA21 | 491513 |  | OS22 | M | . |  | . | . |
| D | KA22 | 491514 |  | OS22 | M | 02495 | - | **08240** | 05090 |
| D | KA22 | 492272 |  | OS22 | M | 02500 | - | **08250** | 05100 |
| M | KA23 | **492264** |  | OS22 | M | 02505 | - | 05160 | 05110 |
| M | KA23 | 510335 | 510335 | OS22 | M | 02555(Nt) | + | NP(dup) | NP(dup) |
| D | KA24 | 510336 | 510336 | OS23 | D | 02555(Ct) | + | NP | NP |
| D | KA24 | 511249 | 511249 | OS23 | D | . |  | . | . |
| M | KA25 | 511250 | 511250 | OS24 | M | 02560 | - | 05260 | 05210 |
| M | KA25 | 521857 |  | OS24 | M | 02620(Ct) | + | notAnno(nf) | 05340(Ct) |
| D | KA26 | 521858 |  | OS24 | M | 02620(Nt) | + | extra | 05340(Nt) |
| D | KA26 | 526145 |  | OS24 | M | . |  | . | . |
| M | KA27 | 526146 |  | OS24 | M | 02625 | - | 05400 | 05350 |
| M | KA27 | 629849 | 629849 | OS24 | M | 03175 | - | 06490 | 06440 |
| D | KA28 | 629850 | 629850 | OS25 | D | . |  | . | . |
| D | KA28 | 630308 | 630308 | OS25 | D | 03180(Ct) | + | 06500(Ct) | 06450(Ct) |
| M | KA29 | 630309 | 630309 | OS26 | M | 03180(Nt) | + | 06500(Nt) | 06450(Nt) |
| M | KA29 | 669897 | 669897 | OS26 | M | 03410(Ct) | r | 06960(Ct) | O6910(Ct) |
| R | KA30A | 669898 | 669898 | OS27A | R | 03410(Nt) | r | 06960(Nt) | O6910(Nt) |
| R | KA30A | 670541 | 670541 | OS27A | R | . |  | . | . |
| R | KA30B | 670542 | 670542 | OS27B | R | 03415(nf) | + | - | - |
| R | KA30B | 671185 | 671185 | OS27B | R | . |  | . | . |
| M | KA31 | 671186 | 671186 | OS28 | M | 03420 | - | 06970 | 06920 |
| M | KA31 | 678949 | 678949 | OS28 | M | 03455 | - | 07040 | 06990 |
| D | KA32 | 678950 | 678950 | OS29 | D | 03460 | - | - | - |
| D | KA32 | 708173 | 708173 | OS29 | D | 03630 | - | - | - |
| M | KA33 | 708174 | 708174 | OS30 | M | 03635 | - | 07050 | 07000 |
| M | KA33 |  | 728931 | OS30 | M | 03755(Nt) | Ct | 07290(Nt) | 07240(Nt) |
| M | KA33 |  | 728932 | OS31 | D | 03755(Ct) | Ct | 07290(Ct) | 07240(Ct) |
| M | KA33 |  | 729500 | OS31 | D | 03765(Ct) | + | 07310(Ct) | stop codon |
| M | KA33 |  | 729501 | OS32 | M | 03765(Nt) | Ct | 07310(Nt) | 07250(Nt) |
| M | KA33 | 738486 | 738500 | OS32 | M | 03805 | - | 07390 | 07330 |
| D | KA34 | 738487 | 738501 | OS33 | D | 03810 | - | NP | NP |
| D | KA34 | **741508** | **741500** | OS33 | D | 03820 | - | NP | NP |
| M | KA35 | 741509 | 741501 | OS34 | M | 03825 | - | 07400 | 07340 |
| M | KA35 | 797351 | 797351 | OS34 | M | 04080 | - | 07930 | 07840 |
| d | KA36 | - | - | OS35 | d |  |  | extra | extra |
| M | KA37 | **797296** | **797299** | OS36 | M | 04085 | - | 08490 | 08390 |
| M | KA37 | 840164 |  | OS36 | M | 04280 | - | 08880 | 08770 |
| d | KA38 | - |  | - | - |  |  | extra |  |
| M | KA39 | **839701** |  | OS36 | M | 04285(nf) | - | 09000 | 08780 |
| M | KA39 |  | 869187 | OS36 | M | 04440(Ct) | + | 09320(Ct) | 09110(Ct) |
| M | KA39 |  | 869188 | OS37 | D | 04440(ctr) | + | 09320(ctr) | 09110(ctr) |
| M | KA39 |  | 870278 | OS37 | D | . |  | . | . |
| M | KA39 |  | 870279 | OS38 | M | 04440(Nt) | + | 09320(Nt) | 09110(Nt) |
| M | KA39 | 890561 |  | OS38 | M | 04560(shrt) | + | 09550(Nt) | 09340(shrt) |
| d | KA40 | - |  | - | - | NP |  | 09550(Ct) | NP |
| M | KA41 | 890562 |  | OS38 | M | 04565 | - | 09560 | 09350 |
| M | KA41 | 898306 | 898306 | OS38 | M | 04595(Nt) | + | 09620(Nt) | 09410(Nt) |
| R | KA42A | 898307 | 898307 | OS39A | R | 04595(ctr) | + | 09620(ctr) | 09410(ctr) |
| R | KA42A | 898507 | 898507 | OS39A | R | . |  | . | . |
| R | KA42B | 898508 | 898508 | OS39B | R | 04595(ctr) | + | NP | 09410(ctr) |
| R | KA42B | 898708 | 898708 | OS39B | R | . |  | . | . |
| M | KA43 | 898709 | 898709 | OS40 | M | 04595(Ct) | + | 09620(Ct) | 09410(Ct) |
| M | KA43 |  | 932241 | OS40 | M | 04750(Ct) | + | 09940(Ct) | 09720(Ct) |
| - | - |  | - | OS41 | d | NP |  | NP | 09720(ctr) |
| M | KA43 |  | **932021** | OS42 | M | 04750(Nt) | + | 09940(Nt) | 09720(Nt) |
| M | KA43 | 985000 |  | OS42 | M | 05070(Ct) | Ct | 10600(Ct) | 10370(Ct) |
| D | KA44 | 985001 |  | OS42 | M | 05070(Nt) | Ct | 10600(Nt) | 10370(Nt) |
| D | KA44 | 990520 |  | OS42 | M | 05085(Ct) | Nt | 10630(Ct) | 10400(Ct) |
| M | KA45 | 990521 |  | OS42 | M | 05085(Nt) | Nt | 10630(Nt) | 10400(Nt) |
| M | KA45 | 992834 | 992834 | OS42 | M | 05100 |  | 10660 | 10430 |
| C | KA46 | 992835 | 992835 | OS43 | C | . |  | . | . |
| C | KA46 | 998022 | 998022 | OS43 | C | . |  | . | . |
| C | KA47 | 998023 | 998023 | OS44 | C | . |  | . | . |
| C | KA47 | 1000603 | 1000603 | OS44 | C | . |  | . | . |
| M | KA48 | 1000604 | 1000604 | OS45 | M | 05105 |  | 10670 | 10440 |
| M | KA48 |  | 1013260 | OS45 | M | 05170(Ct) | + | 10800(Ct) | 10570(Ct) |
| M | KA48 |  | 1013261 | OS46 | D | 05170(Nt) | + | 10800(Nt) | NP |
| M | KA48 | 1026026 |  | OS46 | D | 05240 |  | notAnno | NP |
| d | KA49 | - |  | - | - | - |  | extra | - |
| M | KA50 | 1026027 |  | OS46 | D | 05245 |  | 10950 | NP |
| M | KA50 |  | 1054621 | OS46 | D | 05375(Ct) | + | 11210(Ct) | NP |
| M | KA50 |  | 1054622 | OS47 | M | 05375(Nt) | + | 11210(Nt) | 10570(Nt) |
| M | KA50 | 1066419 | 1066419 | OS47 | M | 05445(Ct) | + | 11350(Ct) | 10710(Ct) |
| D | KA51 | 1066420 | 1066420 | OS48 | D | 05445(Nt) | + | 11350(Nt) | 10710(Nt) |
| D | KA51 | 1068922 | 1068922 | OS48 | D | 05455(Ct) | + | 11370(Ct) | 10730(Ct) |
| M | KA52 | 1068923 | 1068923 | OS49 | M | 05455(Nt) | + | 11370(Nt) | 10730(Nt) |
| M | KA52 | 1070334 | 1070334 | OS49 | M | 05465(Ct) | + | 11390(Ct) | 10750(Ct) |
| D | KA53 | 1070335 | 1070335 | OS50 | D | 05465(Nt) | + | 11390(Nt) | 10750(Nt) |
| D | KA53 | 1074537 | 1074537 | OS50 | D | 05480(Ct) | + | 11420(Ct) | 10780(Ct) |
| M | KA54 | 1074538 | 1074538 | OS51 | M | 05480(Nt) | + | 11420(Nt) | 10780(Nt) |
| M | KA54 | 1134232 |  | OS51 | M | 05755(nf) | Ct | 11980/90 | 11320 |
| D | KA55 | 1134233 |  | OS51 | M | - |  | extra | - |
| D | KA55 | 1134351 |  | OS51 | M | . |  | . | . |
| M | KA56 | 1134352 |  | OS51 | M | 05760 | - | 12020 | 11330 |
| M | KA56 | 1174597 |  | OS51 | M | 05980 | - | 12460 | 11760 |
| D | KA57 | 1174598 |  | OS51 | M | 05985(nf) | - | NP | 11780/70 |
| D | KA57 | 1177533 |  | OS51 | M | . |  | . | . |
| M | KA58 | 1177534 |  | OS51 | M | 05990 | - | 12470 | 11790 |
| M | KA58 | 1189207 |  | OS51 | M | 06050 | - | 12590 | 11910 |
| D | KA59 | 1189208 |  | OS51 | M | 06055 | Nt | NP | 11920 |
| D | KA59 | 1195393 |  | OS51 | M | 06075 | - | NP | 11960 |
| M | KA60 | 1195394 |  | OS51 | M | 06080 | - | 12620 | 11970 |
| M | KA60 |  | 1233192 | OS51 | M | 06275 | - | 13030 | 12380 |
| M | KA60 |  | 1233193 | OS52 | D | 06280 | - | 13040 | NP |
| M | KA60 |  | 1238745 | OS52 | D | 06300 | - | 13080 | NP |
| M | KA60 |  | 1238746 | OS53 | M | 06305 | - | 13090 | 12390 |
| M | KA60 |  | 1253828 | OS53 | M | 06380 | - | 13250 | 12540 |
| M | KA60 |  | 1253829 | OS54 | d | . |  | . | extra |
| M | KA60 |  | 1253862 | OS54 | d | . |  | . | . |
| M | KA60 |  | 1253863 | OS55 | M | 06385 | - | 13260 | 12560 |
| M | KA60 |  | 1277648 | OS55 | M | 06530(Nt) | + | 13560(Nt) | 12860(Nt) |
| - | - |  | - | OS56 | d | NP |  | NP | 12860(Ct) |
| - | - |  | - | OS56 | d | NP |  | NP | 12890(Nt) |
| M | KA60 |  | 1277581 | OS57 | M | 06530(Ct) | + | 13560(Nt) | 12890(Ct) |
| M | KA60 | 1293228 | 1293228 | OS57 | M | 06595(Nt) | + | 13690(Nt) | 13020(Nt) |
| R | KA61A | 1293229 | 1293229 | OS58A | R | 06595(ctr) | + | 13690(ctr) | 13020(ctr) |
| R | KA61A | 1293573 | 1293573 | OS58A | R | . |  |  |  |
| R | KA61B | 1293574 | 1293574 | OS58B | R | 06595(ctr) | + | 13690(ctr) | NP |
| R | KA61B | 1293918 | 1293918 | OS58B | R | . |  |  |  |
| R | KA61C | 1293919 | 1293919 | OS58C | R | 06595(ctr) | + | NP | 13020(ctr) |
| R | KA61C | 1294299 | 1294299 | OS58C | R | . |  |  |  |
| M | KA62 | 1294300 | 1294300 | OS59 | M | 06595(Ct) | + | 13690(Ct) | 13020(Ct) |
| M | KA62 | 1329114 |  | OS59 | M | 06760(Nt) | r | 14020(Nt) | 13350(Nt) |
| r | KA63 | 1329115 |  | OS59 | M | 06760(Ct) | r | 14020(Ct) | 13350(Ct) |
| r | KA63 | 1341054 |  | OS59 | M | 06840 | - | 14690 | 13510 |
| M | KA64 | 1341055 |  | OS59 | M | 06845 | - | 14700 | 13520 |
| M | KA64 | 1440929 |  | OS59 | M | 07365 | - | 15750 | 14560 |
| r | KA65 | 1440930 |  | OS59 | M |  |  |  |  |
| r | KA65 | 1441008 |  | OS59 | M | 07370(Ct) | r | 15760(Ct) | 14570(Ct) |
| M | KA66 | 1441009 |  | OS59 | M | 07370(Nt) | r | 15760(Nt) | 14570(Nt) |
| M | KA66 |  | 1491999 | OS59 | M | 07605(Nt) | r | 16230(Nt) | 15040(Nt) |
| M | KA66 |  | 1492000 | OS60 | r | 07605(Ct) | r | 16230(Ct) | 15040(Ct) |
| M | KA66 |  | 1492752 | OS60 | r | 07620(Nt) | r | 16260(Nt) | 15090(Nt) |
| M | KA66 |  | 1492753 | OS61 | M | 07620(Ct) | r | 16260(Ct) | 15090(Ct) |
| M | KA66 | 1586666 | 1586679 | OS61 | M | 08195 | - | 17410 | 16230 |
| D | KA67 | 1586667 | 1586680 | OS62 | D | NP |  | extra | extra |
| D | KA67 | 1586712 | 1586712 | OS62 | D | . |  | . | . |
| M | KA68 | 1586713 | 1586713 | OS63 | M | 08200 | - | 17440 | 16260 |
| M | KA68 | 1705549 | 1705549 | OS63 | M | 08895(Nt) | + | 18860(Nt) | 17670(Nt) |
| d | KA69 | - | - | OS64 | d | NP |  | 18860(ctr) | 17670(ctr) |
| M | KA70 | 1705550 | 1705550 | OS65 | M | 08895(Ct) | + | 18860(Ct) | 17670 (Ct) |
| M | KA70 | 1720120 | 1720120 | OS65 | M | 08955(Nt) | + | 18980(Nt) | 17790(Nt) |
| D | KA71 | 1720121 | 1720121 | OS66 | D | 08955(Ct) | + | 18980(Ct) | 17790(Ct) |
| D | KA71 |  | 1721177 | OS66 | D | 08960(Nt) | + | 18990(Nt) | 17800(Nt) |
| D | KA71 |  | 1721178 | OS67 | M | 08960(Ct) | + | 18990(Ct) | 17800(Ct) |
| D | KA71 | 1721910 |  | OS67 | M | 08965(Nt) | + | 19000(Nt) | 17810(Nt) |
| M | KA72 | 1721911 |  | OS67 | M | 08965(Ct) | + | 19000(Ct) | 17810(Ct) |
| M | KA72 | 1771462 | 1771462 | OS67 | M | 09245 | - | 19560 | 18370 |

**Supplementary References**

Al-Karadaghi S, Franco R, Hansson M, Shelnutt JA, Isaya G & Ferreira GC (2006) Chelatases: distort to select? *Trends Biochem Sci* **31**: 135–142.

Badel C, Erauso G, Gomez AL, Catchpole R, Gonnet M, Oberto J, Forterre P & Da Cunha V (2019) The global distribution and evolutionary history of the pT26-2 archaeal plasmid family. *Environ Microbiol* **21**: 4685–4705.

Beek Jt, Guskov A & Slotboom DJ (2014) Structural diversity of ABC transporters. *J Gen Physiol* **143**: 419–435.

Belogurov AA, Delver EP, Agafonova OV, Belogurova NG, Lee L-Y & Kado CI (2000) Antirestriction protein Ard (Type C) encoded by IncW plasmid pSa has a high similarity to the “protein transport” domain of TraC1 primase of promiscuous plasmid RP4. *J Mol Biol* **296**: 969–977.

Beltran LC, Cvirkaite-Krupovic V, Miller J*, et al.* (2023) Archaeal DNA-import apparatus is homologous to bacterial conjugation machinery. *Nat Commun* **14**: 666.

Chin C-S, Alexander DH, Marks P, Klammer AA, Drake J, Heiner C, Clum A, Copeland A, Huddleston J & Eichler EE (2013) Nonhybrid, finished microbial genome assemblies from long-read SMRT sequencing data. *Nat Methods* **10**: 563–569.

Deutzmann J, Sahin M & Spormann A (2015) Extracellular enzymes facilitate electron uptake in biocorrosion and bioelectrosynthesis. *mBio* **6**: 10–1128.

Goris J, Konstantinidis KT, Klappenbach JA, Coenye T, Vandamme P & Tiedje JM (2007) DNA–DNA hybridization values and their relationship to whole-genome sequence similarities. *Int J Syst Evol Microbiol* **57**: 81–91.

Goyal N, Zhou Z & Karimi IA (2016) Metabolic processes of *Methanococcus maripaludis* and potential applications. *Microb Cell Fact* **15**: 107.

Goyal N, Widiastuti H, Karimi I & Zhou Z (2014) A genome-scale metabolic model of *Methanococcus maripaludis* S2 for CO 2 capture and conversion to methane. *Mol BioSystems* **10**: 1043–1054.

Gupta R & Brunak S (2002) Prediction of glycosylation across the human proteome and the correlation to protein function. *Pac Symp Biocomput* **2001**: 310–322.

Hendrickson E, Kaul R, Zhou Y, Bovee D, Chapman P, Chung J, Conway de Macario E, Dodsworth J, Gillett W & Graham D (2004) Complete genome sequence of the genetically tractable hydrogenotrophic methanogen *Methanococcus maripaludis*. *J Bacteriol* **186**: 6956–6969.

Hendrickson EL, Kaul R, Zhou Y*, et al.* (2004) Complete genome sequence of the genetically tractable hydrogenotrophic methanogen *Methanococcus maripaludis*. *J Bacteriol* **186**: 6956–6969.

Herrou J & Crosson S (2013) Molecular structure of the metalloprotein RicA, a Rab2-binding virulence effector. *Biochemistry* **52**: 9020–9028.

Iverson TM, Alber BE, Kisker C, Ferry JG & Rees DC (2000) A closer look at the active site of γ-class carbonic anhydrases: high-resolution crystallographic studies of the carbonic anhydrase from *Methanosarcina thermophila*. *Biochemistry* **39**: 9222–9231.

Jenkins J, Mayans O & Pickersgill R (1998) Structure and evolution of parallel β-helix proteins. *J Struct Biol* **122**: 236–246.

Jeyakanthan J, Rangarajan S, Mridula P, Kanaujia SP, Shiro Y, Kuramitsu S, Yokoyama S & Sekar K (2008) Observation of a calcium-binding site in the γ-class carbonic anhydrase from *Pyrococcus horikoshii*. *Acta Crystallogr D* **64**: 1012–1019.

Jiao M, He W, Ouyang Z*, et al.* (2023) Mechanistic and structural insights into the bifunctional enzyme PaaY from *Acinetobacter baumannii*. *Structure* **31**: 935–947.

Kalvari I, Argasinska J, Quinones-Olvera N, Nawrocki E, Rivas E, Eddy S, Bateman A, Finn R & Petrov A (2018) Rfam 13.0: shifting to a genome-centric resource for non-coding RNA families. *Nucleic Acids Res* **46**: D335–D342.

Kanehisa M, Sato Y, Furumichi M, Morishima K & Tanabe M (2019) New approach for understanding genome variations in KEGG. *Nucleic Acids Res* **47**: D590–D595.

Katoh K & Standley DM (2013) MAFFT multiple sequence alignment software version 7: improvements in performance and usability. *Mol Biol Evol* **30**: 772–780.

Kawaichi S, Kotoky R, Fiutowski J & Rotaru AE (2024) Adaptation of a methanogen to Fe0 corrosion via direct contact. *NPJ Biofilms Microbiomes* **10**: 100.

Keswani J, Orkand S, Premachandran U, Mandelco L, Franklin M & Whitman W (1996) Phylogeny and taxonomy of mesophilic *Methanococcus* spp. and comparison of rRNA, DNA hybridization, and phenotypic methods. *Int J Syst Evol Microbiol* **46**: 727–735.

Köhrer C, Srinivasan G, Mandal D, Mallick B, Ghosh Z, Chakrabarti J & Rajbhandary UL (2008) Identification and characterization of a tRNA decoding the rare AUA codon in. *Rna* **14**: 117–126.

Lefort V, Desper R & Gascuel O (2015) FastME 2.0: a comprehensive, accurate, and fast distance-based phylogeny inference program. *Mol Biol Evol* **32**: 2798–2800.

Loenen WA, Dryden DT, Raleigh EA & Wilson GG (2014) Type I restriction enzymes and their relatives. *Nucleic Acids Res* **42**: 20–44.

Lohner ST, Deutzmann JS, Logan BE, Leigh J & Spormann AM (2014) Hydrogenase-independent uptake and metabolism of electrons by the archaeon *Methanococcus maripaludis*. *ISME J* **8**: 1673–1681.

Lyu Z, Jain R, Smith P, Fetchko T, Yan Y & Whitman WB (2016) Engineering the autotroph *Methanococcus maripaludis* for geraniol production. *ACS Synth Biol* **5**: 577–581.

Makarova KS & Koonin EV (2013) Archaeology of eukaryotic DNA replication. *Cold Spring Harbor Perspect Biol* **5**: a012963.

Mandal D, Kohrer C, Su D, Russell SP, Krivos K, Castleberry CM, Blum P, Limbach PA, Soll D & RajBhandary UL (2010) Agmatidine, a modified cytidine in the anticodon of archaeal tRNA(Ile), base pairs with adenosine but not with guanosine. *Proc Natl Acad Sci U S A* **107**: 2872–2877.

Meier-Kolthoff JP, Carbasse JS, Peinado-Olarte RL & Göker M (2022) TYGS and LPSN: a database tandem for fast and reliable genome-based classification and nomenclature of prokaryotes. *Nucleic Acids Res* **50**: D801–D807.

Mori K, Tsurumaru H & Harayama S (2010) Iron corrosion activity of anaerobic hydrogen-consuming microorganisms isolated from oil facilities. *J Biosci Bioeng* **110**: 426–430.

Overbeek R, Olson R, Pusch GD, Olsen GJ, Davis JJ, Disz T, Edwards RA, Gerdes S, Parrello B & Shukla M (2014) The SEED and the rapid annotation of microbial genomes using subsystems technology (RAST). *Nucleic Acids Res* **42**: D206–D214.

Park HM, Park JH, Choi JW, Lee J, Kim BY, Jung CH & Kim JS (2012) Structures of the γ-class carbonic anhydrase homologue YrdA suggest a possible allosteric switch. *Acta Crystallogr D* **68**: 920–926.

Pfeiffer F & Oesterhelt D (2015) A manual curation strategy to improve genome annotation: application to a set of haloarchael genomes. *Life* **5**: 1427–1444.

Pfeiffer F, Losensky G, Marchfelder A, Habermann B & Dyall‐Smith M (2020) Whole‐genome comparison between the type strain of *Halobacterium salinarum* (DSM 3754T) and the laboratory strains R1 and NRC‐1. *Microbiologyopen* **9**: e974.

Poehlein A, Heym D, Quitzke V, Fersch J, Daniel R & Rother M (2018) Complete genome sequence of the *Methanococcus maripaludis* type strain JJ (DSM 2067), a model for selenoprotein synthesis in archaea. *Genome Announce* **6**: 10–1128.

Prabha A & Balaji PV (2021) Characterization of left-handed beta helix-domains, and identification and functional annotation of proteins containing such domains. *Proteins* **89**: 6–20.

Soler N, Marguet E, Cortez D, Desnoues N, Keller J, van Tilbeurgh H, Sezonov G & Forterre P (2010) Two novel families of plasmids from hyperthermophilic archaea encoding new families of replication proteins. *Nucleic Acids Res* **38**: 5088–5104.

Staudenmaier H, Van Hove B, Yaraghi Z & Braun V (1989) Nucleotide sequences of the fecBCDE genes and locations of the proteins suggest a periplasmic-binding-protein-dependent transport mechanism for iron (III) dicitrate in *Escherichia coli*. *J Bacteriol* **171**: 2626–2633.

Tsurumaru H, Ito N, Mori K, Wakai S, Uchiyama T, Iino T, Hosoyama A, Ataku H, Nishijima K & Mise M (2018) An extracellular [NiFe] hydrogenase mediating iron corrosion is encoded in a genetically unstable genomic island in *Methanococcus maripaludis*. *Sci Rep* **8**: 15149.

Uchiyama T, Ito K, Mori K, Tsurumaru H & Harayama S (2010) Iron-corroding methanogen isolated from a crude-oil storage tank. *Appl Environ Microbiol* **76**: 1783–1788.

Val-Calvo J, Luque-Ortega JR, Crespo I, Miguel-Arribas A, Abia D, Sánchez-Hevia DL, Serrano E, Gago-Córdoba C, Ares S & Alfonso C (2018) Novel regulatory mechanism of establishment genes of conjugative plasmids. *Nucleic Acids Res* **46**: 11910–11926.

Wang X, Greenfield P, Li D, Hendry P, Volk H & Sutherland TD (2011) Complete genome sequence of a nonculturable *Methanococcus maripaludis* strain extracted in a metagenomic survey of petroleum reservoir fluids. *J Bacteriol* **193**: 5595.

Whitman WB, Shieh J, Sohn S, Caras DS & Premachandran U (1986) Isolation and characterization of 22 mesophilic methanococci. *Syst Appl Microbiol* **7**: 235–240.
