# Supplementary material for: From genes to Black Rust: genomic insights into corrosive methanogens
Source: FEMS Microbes. 2025 Nov 17;6:xtaf018. doi: 10.1093/femsmc/xtaf018 (PMC12667266; doi:10.1093/femsmc/xtaf018)
Supplement: xtaf018_Supplemental_Files [file xtaf018_supplemental_files.zip › FEMSMC-2025-029.R1 supplementary data - reviews and author response.docx]

**Reviews of manuscript FEMSMC-2025-029**

**Reviewer 1**

**Recommendation: Minor Revision**

Dear Authors,

I thoroughly enjoyed reading and reviewing your manuscript on the genomics of corrosive methanogenic archaea. This is a timely and highly relevant study, and I was impressed by its depth and the collaborative effort involved.

The MIC island in certain methanogens has received increasing attention from both the scientific community and industry practitioners, and your work provides a robust framework for understanding the genes implicated in corrosion as well as the transfer of this trait between species. I am confident that the manuscript will attract significant interest and visibility.

Below, I provide several general comments and suggestions for improvement, followed by specific line-by-line edits. I hope you find these helpful in further strengthening your manuscript.

Kind regards,

Your reviewer

________________________________________

General Comments and Suggestions

1. Clarification of terminology

The term 'MIC island', originally introduced by Tsurumaru and colleagues to describe a 14-gene (12 kb) region in M. maripaludis OS7, has since been widely adopted. You make a useful distinction between the 'MIC island' and the 'MIC core' (6–7 genes essential for corrosion), but I recommend emphasizing this distinction more consistently. For example, clearly defining the MIC core (perhaps in the text and in Fig. 2) would help readers follow the terminology throughout.

2. Conciseness and focus

The manuscript presents an impressive breadth of information — from the MIC core and the proposed new role of FohE, to neighboring genes, cell wall synthesis, and glycosylation. While these insights are valuable, at times the density of information felt - quite frankly - overwhelming. Trimming or condensing certain sections could make the manuscript more accessible without diminishing its impact.

3. Mechanistic emphasis

To date, synthesis of the [NiFe]-hydrogenase (FohAB) remains the only validated corrosion mechanism in methanogens. To my knowledge, no evidence shows that methanogens lacking this enzyme can corrode steel. Do the authors agree? Highlighting this point would further emphasize the significance of your comprehensive analysis of the MIC core.

4. Practical relevance for industry

The qPCR assay developed by Lahme et al. (2021) for detecting the [NiFe]-hydrogenase large subunit is now employed by multiple commercial entities in North America and Europe to assess MIC for their international customers in oil- or water-bearing systems. Readers — especially industry practitioners — may be interested to know whether this assay covers the corrosive strains analyzed in your study. Including a short discussion, or even a brief speculation, could enhance the manuscript’s practical relevance (optional suggestion).

5. Interesting observations

I was intrigued by your finding that the MIC island can be lost so rapidly once selection pressure is removed. Equally interesting is the discovery that a hydrophilic C-terminus of FohB with potential N-glycosylation sites appears to be a defining feature of the MIC hydrogenase family, with certain mutations leading to a loss in the ability to get glycosylated. These observations raise exciting new questions about how such genetic features may influence strain corrosiveness, which I hope you (or others) will address in the future.

Specific Comments and Suggested Edits

Line 29: Add hyphen → “Methanobacterium-affiliated.”

Line 67: Please tone down this sentence. Microorganisms other than SRB have been implicated in MIC for decades; even von Wolzogen Kühr himself speculated on cathodic depolarization of iron by methanogens (C. A. H. von Wolzogen Kuhr, Corrosion 1961, 17(6), 119–125. https://doi.org/10.5006/0010-9312-17.6.119). Referencing only recent studies could give the misleading impression that this understanding is new.

Line 68: Use “SRB” rather than “SRBs.” The plural is “sulfate-reducing bacteria.”

Line 79: Use “electron withdrawal.”

Line 87: Suggested phrasing: “Providing H₂ as the sole electron donor in laboratory incubations led to …”

Line 90: Consider citing Lahme et al. (2025, IBB Journal), if appropriate, as a more recent reference.

Line 108: Improve phrasing → “… the genomes of which …”

Line 196: Correct to “Strain OS7.”

Line 252: Capitalize → “Sanger sequencing.”

Line 487: Insert missing word → “non-corrosive ones.”

Line 609: Please clarify how climate change may influence microbial corrosion, if this is the intended point. Otherwise, consider omitting the reference to climate change and the work of Tiedje et al. (2022), as the mentioned study does not explicitly address a connection between climate and corrosion.

Line 611: Reframe the description of the landmark Tsurumaru study to emphasize its unique mechanistic contribution (demonstrating for the first rime how methanogens may cause MIC), rather than suggesting it first proved methanogens could corrode iron.

Line 1095: Please check the formatting of bicarbonate; correct form is HCO₃⁻.

**Reviewer 2: Florin Musat, Aarhus University**

**Recommendation: Minor Revision**

In this manuscript, Kleinbub et al. investigate the genomic basis of microbially influenced corrosion (MIC) in methanogenic archaea, with a particular focus on the corrosive strain Methanococcus maripaludis Mic1c10. Through genome sequencing and detailed comparative genomics, the authors identify and analyze the MIC island and the associated hydrogenase genes, placing them within a conserved MIC core found in both Methanococci and Methanobacteria. Their analyses reveal multiple mechanisms for mobilization of the MIC core, including horizontal gene transfer and a newly described MIC transposon. Comparative studies with other corrosive methanogens, including the highly corrosive strain IM1, highlight shared and divergent genetic neighborhoods around MIC genes. The work integrates bioinformatic analyses of cell wall glycosylation pathways and microscopy-based observations, leading to updated models of methanogen-induced corrosion that links cell-surface properties with corrosion activity.

This is a careful and well-structured study, providing in-depth genetic insights into corrosive methanogens. The combination of rigorous comparative genomics with thoughtful interpretation makes this work a valuable contribution to the field. Figures are of exceptional quality. I congratulate the authors for an excellent work.

I have but a handful of minor comments.

1. Lines 71 – forward: I think a clearer distinction can be made around these lines between direct MIC (through hydrogenases or direct electron uptake) and indirect MIC (through the production of corrosive agents like H2S), for example as done by Enning et al., Environ Microbiol 2012, 14:1772-1787.

2. Line 85: ‘… in turn accelerating corrosion …’

3. Lines 144-145: this section has the subtitle ‘(isolation of Mic1c10mut1)’, but it only becomes clear later on that growth on H2 led to loss of the MIC island, creating a bit of confusion. I would add at the beginning of this section a sentence like:

Cultivation of Mic1c10 on H2 led to loss of the MIC island, as revealed by subsequent genome sequencing (see below). The new mutant strain was named Mic1c10mut1

Or something to that effect.

4. Line 591: could it be that cells became encased in their own corrosion products, and this is the reason cells were not found associated with the surface?

5. Line 608: replace ‘ensure that’ with ‘are forecasting that’. Next line: ‘and may be even accelerated by changing climate’

6. Line 657 and forward: I would use model(s) instead of mode(s)

7. Figure 7, scale bars caption: use μm for micrometer, instead of μM (micromolar).

**Associate Editor Recommendation: Minor Revision**

**EIC Decision: Minor Revision**

**Author's Response to Decision Letter for (FEMSMC-2025-029)**

Dear Editor,

Thank you very much for the opportunity to resubmit our article. Please find below our detailed response to the reviewer’s comments.

We will update all relevant sections during the resubmission process and provide both a clean version of the revised manuscript and a tracked version highlighting the changes made.

I look forward to your response and wish you a great week ahead.

Best regards,

Andrea Koerdt

Response to Reviewers

Dear Prof. Jass,

Thank you for the opportunity to revise our manuscript entitled “From Genes to Black Rust: Genomic Insights into Corrosive Methanogens” (Manuscript ID: FEMSMC-2025-029). We appreciate the thoughtful comments provided by you and the reviewers. We have carefully considered each point and revised the manuscript accordingly. Below, we provide a detailed point-by-point response to the reviewers’ comments. Our revised manuscript is accompanied by a second version that has all changes made to the manuscript marked with ‘Track Changes’. For reference, line pages referenced in our responses refer to the revised manuscript.

Reviewer #1

A. General Comments and Suggestions

Comment 1: Clarification of terminology

The term 'MIC island', originally introduced by Tsurumaru and colleagues to describe a 14-gene (12 kb) region in M. maripaludis OS7, has since been widely adopted. You make a useful distinction between the 'MIC island' and the 'MIC core' (6–7 genes essential for corrosion), but I recommend emphasizing this distinction more consistently. For example, clearly defining the MIC core (perhaps in the text and in Fig. 2) would help readers follow the terminology throughout.

Response:

We are very thankful for the feedback from the reviewer regarding the ‘MIC island’ and the ‘MIC core’. This was an important distinction for us to get across in the manuscript and we are happy to make it more clear. To address this we have:

- defined explicitly the term ‘MIC core’ in the main text (see line 356 and following), clarifying that it refers to the 6 – 7 genes most directly implicated in corrosion activity by methanogens.

“These six or seven conserved genes likely represent a core set of genes required for FohAB to function, hereafter referred to as the MIC core. The MIC core (Fig. 2A, orange-colored genes) is distinguished from the broader MIC island previously described by Tsurumaru and colleagues (Tsurumaru et al., 2018).”

- updated the legend of Figure 2 (line 975) to define the ‘MIC core’ more clearly.

“The MIC core (orange, genes labelled by the respective encoded proteins) refers to a conserved set of 6 – 7 genes, including fohAB and associated downstream genes that are hypothesized to be essential for corrosion.”

- Added clear language in the discussion on line 589 “ … in the newly defined MIC core …”

- reviewed the manuscript to ensure that the terms ‘MIC island’ and ‘MIC core’ are used consistently.

Comment 2: Conciseness and focus

The manuscript presents an impressive breadth of information – from the MIC core and the proposed new role of FohE, to neighboring genes, cell wall synthesis, and glycosylation. While these insights are valuable, at times the density of information felt - quite frankly - overwhelming. Trimming or condensing certain sections could make the manuscript more accessible without diminishing its impact.

Response:

In order to make the manuscript more digestible, as Reviewer 1 has commented on, we have moved the section “pT26-2 related integrative elements and metal chelate gene clusters in M. maripaludis” into the supplemental information as Supplemental Text S9. In its place, we have included a short paragraph pointing out the relevance of this material with respect to Figure 1 entitled “A mosaic of integrative gene clusters” on line 412. We believe this edit will help in keeping the focus on the central theme of biocorrosion and place information not directly related to the corrosion process in the supplemental. As all genetic differences between the M. maripaludis strains may in some way influence corrosion, we find it still important to include the information of pT26-2 plasmids, metal chelate clusters, etc. in the supplemental.

As noted below, we have also removed the reference regarding climate change to keep the manuscript focused on the biocorrosion topic.

Comment 3: Mechanistic emphasis

To date, synthesis of the [NiFe]-hydrogenase (FohAB) remains the only validated corrosion mechanism in methanogens. To my knowledge, no evidence shows that methanogens lacking this enzyme can corrode steel. Do the authors agree? Highlighting this point would further emphasize the significance of your comprehensive analysis of the MIC core.

Response:

We agree that the [NiFe] hydrogenase (FohAB) remains the only molecular species known in methanogens, which can putatively accelerate the corrosion process. It is important to note, that further biochemical work is required to verify the corrosion mechanism. Already in 1987, methanogens had been recognized as having the ability to carry out methanogenesis with elemental iron as a sole electron donor (Daniels et al 1987). For example, Methanosarcina barkeri used in that study does not contain FohAB, yet produced methane above background levels. More recently, Holmes et al have observed (electrobio)corrosion in methanogens without FohAB (and without outer-surface cytochromes) suggesting that there are other undefined molecular species that can promote corrosion (PMID: 38827509). Therefore, other mechanisms may play a role in different methanogens but have not yet been elucidated. We agree though with the reviewer that more emphasis could be placed on the presence of fohAB genes being the only experimental data in methanogens that can mechanistically link microbial growth to corrosion.

We have revised the manuscript to emphasize this point. Specifically, we have added the following sentence to the Discussion section on line 584 and following:

“The presence of genes encoding the MIC hydrogenase remains the only validated experimental evidence mechanistically linking methanogens to the corrosion of metal (Tsurumaru et al., 2018, Holten et al., 2021, Lahme et al., 2021, Kawaichi et al., 2024, Lahme & Aguas, 2025).”

Comment 4: Practical relevance for industry

The qPCR assay developed by Lahme et al. (2021) for detecting the [NiFe]-hydrogenase large subunit is now employed by multiple commercial entities in North America and Europe to assess MIC for their international customers in oil- or water-bearing systems. Readers – especially industry practitioners – may be interested to know whether this assay covers the corrosive strains analyzed in your study. Including a short discussion, or even a brief speculation, could enhance the manuscript’s practical relevance (optional suggestion).

Response:

We thank the reviewer for highlighting the practical relevance of the micH qPCR assay developed by Lahme et al. (2021) and the encouragement to provide speculation on this topic. As the reviewer has also mentioned conciseness, we wish not to add content to the manuscript. Therefore, we respectfully decline commenting on the industrial application in this manuscript but will surely keep it in mind for future manuscripts. For this manuscript, we wish to only further promote the use of the assay as stated at the end of the manuscript (starting on line 708).

Comment 5: Interesting observations

I was intrigued by your finding that the MIC island can be lost so rapidly once selection pressure is removed. Equally interesting is the discovery that a hydrophilic C-terminus of FohB with potential N-glycosylation sites appears to be a defining feature of the MIC hydrogenase family, with certain mutations leading to a loss in the ability to get glycosylated. These observations raise exciting new questions about how such genetic features may influence strain corrosiveness, which I hope you (or others) will address in the future.

Response:

We thank the reviewer for the thoughtful and encouraging comments regarding our findings. The rapid loss of the MIC island upon removal of the selection pressure was a notable observation which highlights the dynamic nature of these genetic elements. Similarly, the N-glycosylation of the C-terminus of FohB as a characteristic feature of the MIC hydrogenase family provides promising approaches for clarifying the molecular mechanisms of methanogen-induced MIC. We agree that these results raise relevant questions about the relationship between genetic traits and corrosiveness of certain strains. Although a comprehensive investigation of these questions is beyond the scope of the current study, we plan to conduct further research to better understand how these genetic features may influence the corrosiveness of methanogenic strains.

B. Specific Comments and Suggested Edits

We appreciate the reviewer’s attention to detail. We have carefully addressed all specific comments and suggested edits in the order listed in the review. Specifically, we have:

- Added a hyphen to “Methanobacterium-affiliated” on lines 39 and 108.

- Reworded the sentence starting on line 67 as suggested and now cite von Wolozogen Kuhr.

“It is now more widely recognized that in addition to SRB and other bacteria, many different microbes can participate in MIC including archaea, fungi, algae, and lichen, building on a hypothesis of corrosion influenced by microbes posited nearly a century ago (von Wolzogen Kühr & van der Vlugt, 1934, Puentes-Cala et al., 2022, Khan et al., 2023)”

- Changed “SRBs” to “SRB” on lines 63 and 68.

- Used “electron withdrawal” instead of “electron withdraw” on line 80.

- Rephrased the sentence on line 88 according to the reviewer’s suggestion:

“Providing H2 as the sole electron donor in laboratory incubations led to the loss of this genetic element in those strains.”

- Referenced Lahme et al. (2025) as suggested and agree that it is a relevant and very recent reference. We have now cited this work on lines 91-2 to provide an updated context regarding the c-type cytochromes and their key functions in MIC.

- Improved phrasing on lines 110-1

“… (the genomes of which were not sequenced at the time) …”.

- Corrected “strain O7” to “strain OS7” on line 202.

- Capitalized “Sanger sequencing” on line 258.

- Inserted the missing word “corrosive” to the sentence on line 460-1:

“Overall, the differences in both glycan biosynthesis gene clusters indicates that the glycan composition differs across corrosive M. maripaludis strains as well as non-corrosive ones.”

- Removed the statement referencing climate change and citation of Tiedje et al. (2022) from the manuscript to maintain clarity and focus, as we agree with the reviewer’s observation that the reference does not directly support the discussion on microbial corrosion.

- Revised the manuscript text to clearly highlight the contribution of Tsurumaru et al. (2018) in the finding how methanogens contribute to corrosion starting on line 582:

“With the identification of the fohAB genes and their correlation to the oxidation of iron, Tsurumaru et al. in 2018 provided the first direct genetic evidence for describing how methanogens contribute to MIC (Tsurumaru et al., 2018).”

- Corrected the formatting of bicarbonate to HCO3- on line 1059 and in Figure 6.

Reviewer #2

Comment 1: Lines 71 – forward: I think a clearer distinction can be made around these lines between direct MIC (through hydrogenases or direct electron uptake) and indirect MIC (through the production of corrosive agents like H2S), for example as done by Enning et al., Environ Microbiol 2012, 14:1772-1787.

Response:

We have revised the relevant paragraph to clearly distinguish between direct MIC and indirect MIC following the example by Enning et al. (2012). To enhance this clarity, we have also added the term “indirect MIC” on line 67 and “direct MIC” on line 76.

Comment 2: Line 85: ‘… in turn accelerating corrosion …’

Response:

The reviewer makes a valid point, in that corrosion under anaerobic conditions is technically described by equation 2 (line 59) and not “caused” by the action of the hydrogenase. Since the MIC hydrogenase merely catalyzes the reaction in equation 2, the phrasing “greatly accelerating” has been used in the revised manuscript. The updated sentence starting on line 86 now reads:

“Around the same time, the discover of an unstable genetic island (MIC island) encoding an extracellular [NiFe]-hydrogenase (MIC hydrogenase) in the closely related methanogenic archaeal strains Methanococcus maripaludis OS7 and KA1 provided direct genetic evidence that microbes have evolved specific mechanisms to catalyze H2 production on elemental iron, in turn greatly accelerating corrosion (Tsurumaru et al., 2018).”

Comment 3: Lines 144-145: this section has the subtitle ‘(isolation of Mic1c10mut1)’, but it only becomes clear later on that growth on H2 led to loss of the MIC island, creating a bit of confusion. I would add at the beginning of this section a sentence like:

Cultivation of Mic1c10 on H2 led to loss of the MIC island, as revealed by subsequent genome sequencing (see below). The new mutant strain was named Mic1c10mut1

Or something to that effect.

Response:

Thank you for this very helpful comment. To improve clarity, we have adopted the reviewer’s proposal and revised the beginning of the section by adding the following sentence starting on line 147:

“Cultivation of M. maripaludis Mic1c10 on H2 resulted in the loss of the MIC island, as revealed by subsequent genome sequencing (see below). The resulting mutant strain was named Mic1c10mut1, consistent with the spontaneous mutant obtained in strain OS7 (M. maripaludis OS7mut1) described by Tsurumaru et al. (2018).”

Comment 4: Line 591: could it be that cells became encased in their own corrosion products, and this is the reason cells were not found associated with the surface?

Response:

It is a feasible idea that M. maripaludis cells become embedded within their own corrosion products and were therefore not detected to be associated with the corroded surface. As described in the manuscript, we observed coccoid cells on the walls and edges of crevices in the corrosion layer. These observations may indeed suggest that cells and/or biofilms were encased by corrosion products and that the few cells observed stem from the release of cells due to cracking in the corrosion surface. We have now briefly addressed this speculative idea in the discussion section of the manuscript (starting on line 666 and following).

“Cells of Mic1c10, however, could only be identified in crevices of the corrosion layer and not on exposed surfaces. These observations suggest that Mic1c10 cells may become embedded within corrosion products over time (An et al., 2021), in contrast to IM1 where vast amounts of cells and EPS were visible on corrosion surfaces. Therefore, the accumulation of iron granules in Mic1c10 static cultures may alternatively be explained by an encapsulating corrosion layer.”

Comment 5: Line 608: replace ‘ensure that’ with ‘are forecasting that’. Next line: ‘and may be even accelerated by changing climate’.

Response:

Thank you for the suggestion. We have decided to rephrase the sentence starting on line 580:

“The continued dependence on metallic infrastructure in addition to the transition to a H2 economy utilizing existing and new steel pipelines forebode that this threat will remain relevant into the future (Jemblie et al., 2025).”

In line with the comment from Reviewer #1, we have removed the statement “[…] and may be influenced by a changing climate (Tiedje et al., 2022).”

Comment 6: Line 657 and forward: I would use model(s) instead of mode(s)

Response:

The suggestion is well taken, yet we find that the use of the word “mode” is most appropriate, and we have left the text in its original form. This is an important distinction for us. The MIC hydrogenase is at the center of the Mi-MIC model of biocorrosion. The model predicts that the hydrogenase, irrespective of whether it is cell-surface attached or freely diffusible, attaches to metal surfaces and abstracts electrons for driving the catalytic reduction of protons to form hydrogen. Therefore, there are two modes of action within the model of Mi-MIC involving the MIC hydrogenase.

Comment 7: Figure 7, scale bars caption: use μm for micrometer, instead of μM (micromolar).

Response:

Thank you for catching this small error. Figure 7 has now been corrected in the revised manuscript.

We hope that our revisions and detailed responses have satisfactorily addressed the reviewers’ comments. We thank you again for the opportunity to revise our manuscript and look forward to your feedback.

Sincerely,

The Corresponding Authors

Gabriela Alfaro Espinosa and Andrea Koerdt

**Reviews of manuscript FEMSMC-2025-029.R1**

**Reviewer 1**

**Recommendation: Accept**

Dear Mrs. Kleinbub, dear Mr. Braymer an colleagues,

I was happy to read that you have found my suggested edits useful. Again, congratulations on a work done very well.

The manuscript is still - after the slight shortening - pretty hefty, but I understand that you prefer to keep the presentation of your work rather detailed. Maybe this just has to be, given the nature of this 'all-encompassing' study.

I feel that my comments have overall been adequately addressed and wish you interesting and fruitful discussions with your readers.

Kind regards,

Your reviewer

**Reviewer 2: Florin Musat, Aarhus University**

**Recommendation: Accept**

The authors addressed all my comments, I'd recommend this manuscript to be accepted

**Associate Editor Recommendation: Accept**

**EIC Decision: Accept**
